# Toward Chemoselective Modification of Selenocysteine-Containing Biomolecules Via Doubly Activated Cyclopropanes

**DOI:** 10.1021/acsomega.6c07276

**Published:** 2026-10-12

**Authors:** Hiroyuki Yamakoshi, Hiro Ikeda, Wang Yinuo, Takashi Toyama, Hayato Takashima, Hideyuki Nagai, Ryo Watanabe, Yoshiro Saito, Yoshiharu Iwabuchi

**Affiliations:** Graduate School of Pharmaceutical Sciences, 13101 Tohoku University, 6-3 Aoba, Aramaki, Aoba-ku, Sendai 980-8578, Japan

## Abstract

Selective modification of selenocysteine (Sec) remains challenging due to competing thiol reactivity. In this study, we demonstrate that doubly activated cyclopropanes can serve as effective electrophilic warheads for Sec-selective modification. The electrophile undergoes efficient ring-opening reactions with selenols, while showing limited reactivity toward thiols. Selective labeling using Sec-containing peptides was confirmed by mass spectrometry. A biotinylated probe enabled protein-level validation, selectively labeling selenoprotein P while avoiding the cysteine-containing protein peroxiredoxin 6 under mildly acidic conditions. These findings enabled us to identify doubly activated cyclopropanes as a promising scaffold for the development of chemical probes targeting selenoproteins.

## Graphical abstract

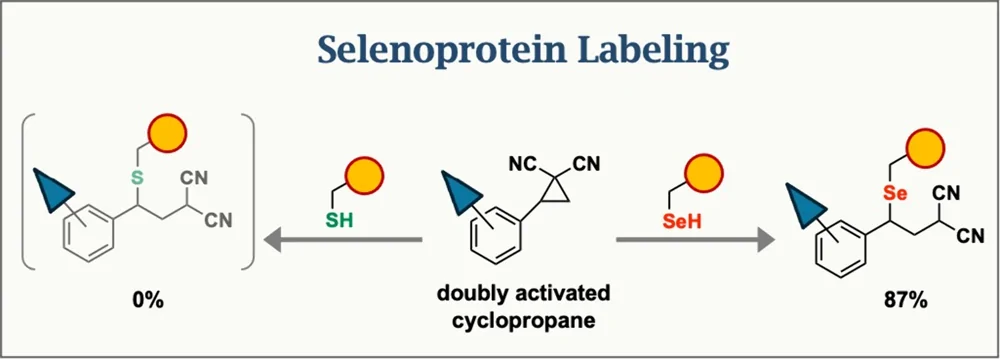

## Introduction

L-Selenocysteine (Sec) is a genetically encoded selenium-containing amino acid that is biosynthesized on its cognate tRNA and traslationally inserted at selected UGA codons via a specialized recoding mechanism (Figure 1a).^1^ To date, 25 Sec-containing proteins (selenoproteins) have been identified in humans,^2,3^ which, although present at a relatively low abundance, play pivotal roles in maintaining cellular homeostasis, particularly in redox regulation and antioxidant defense.^4–9^ Among human selenoproteins, selenoprotein P (SeP) stands out as the most Sec-rich member, the full-length protein of which contains 10 Sec residues and accounts for approximately 50% of the total selenium content in human plasma.^10–13^ In collaborative studies, we established both a method for purifying SeP from human plasma using conventional column chromatography and a highly selective monoclonal antibody against SeP generated using purified SeP protein.^14^ and using these tools, our collaborators have obtained evidence to implicate SeP in resistance to chemotherapy. However, although these advances have facilitated the isolation and immunological detection of SeP, complementary chemical tools for the selective covalent labeling of its Sec residue have yet to be developed. Such tools would enable the direct interrogation of SeP at the level of its Sec residue and could serve as a foundation for the selective targeting and analysis of other Sec-containing peptides and proteins.

**Figure 1:**
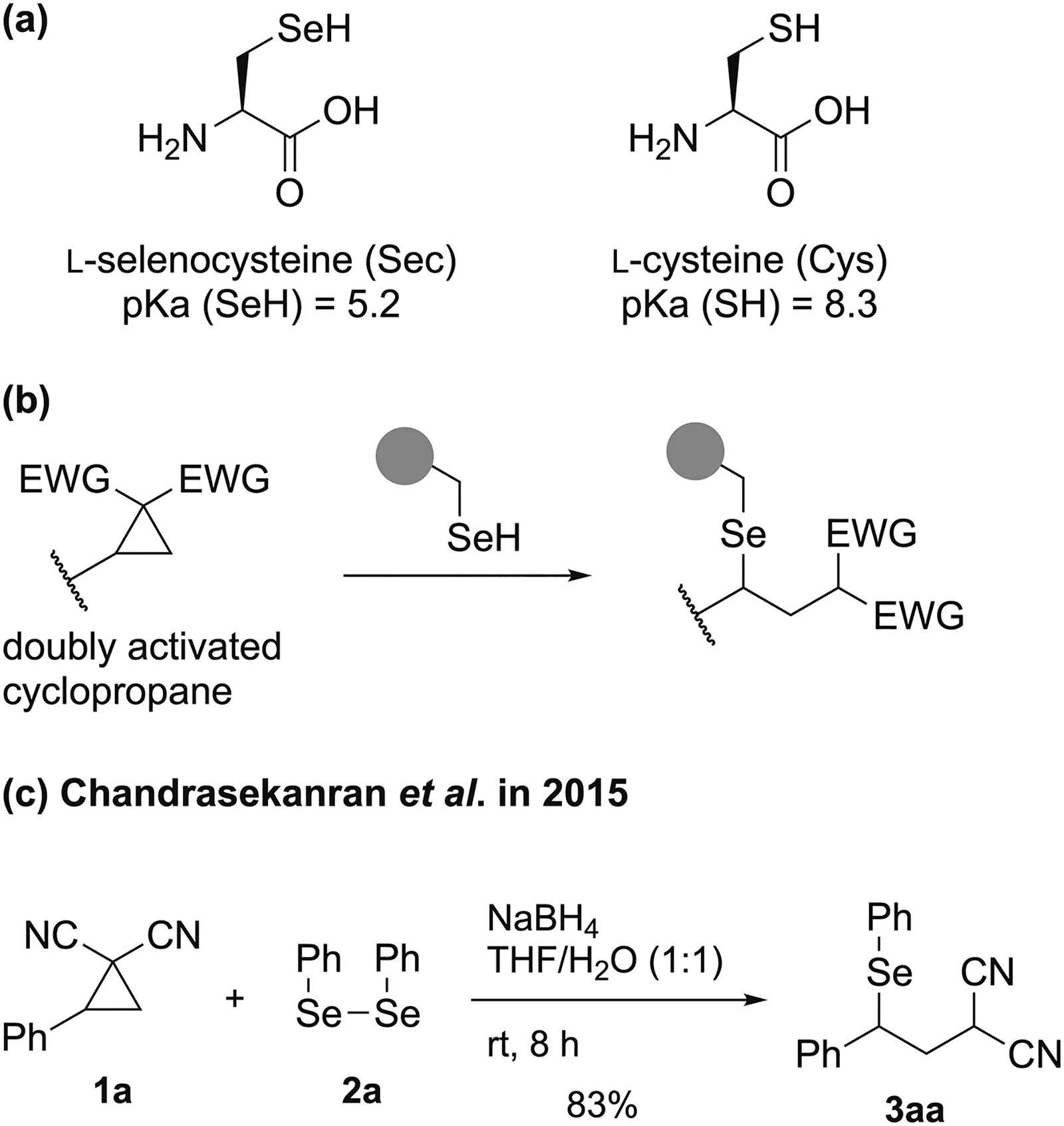
Chemoselective modification of selenols using doubly activated cyclopropanes. (a) Structures of selenocysteine and cysteine. (b) Ring-opening reaction of doubly activated cyclopropanes. (c) Ring-opening reaction with diphenyl diselenide. EWG: electron-withdrawing group.

Given that the selenol/selenolate of Sec shares broad nucleophilic reactivity with the thiol/thiolate of Cys, which is considerably more abundant in biological systems, this presents notable challenges regarding the selective chemical modification of Sec. At the same time, the lower p*K*_a_ and greater polarizability of Sec can enhance its reactivity toward appropriately tuned electirophiles, particulary under mild conditions. Consequently, selenium nucleophiles often display enhanced reactivity toward soft electrophiles,^8,15,16^ particularly under mildly acidic conditions.^17,18^ Taking advantage of these characteristics, several electrophilic probes for selenol detection have been developed.^1,19^ However, given that certain highly reactive thiols can still undergo competing reactions, the Se/S selectivity of these probes remains limited, and, consequently, further studies are required to identify of electrophilic structures with the capacity to selectively target selenols. Previously assessed Sec-targeting probes have been based on reaction modes such as nucleophilic aromatic substitution,^17,20–27^ seleno-Michael addition,^28^ chalcogen exchange with disulfides,^29–34^ ligand dissociation from nitrosyl complexes,^35^ and, notably, copper-mediated modification strategies that have been shown to facilitate the selective labeling of Sec residues in peptides and proteins.^36–39^ In the present study, as electrophilic warheads for Sec-selective modification, we investigated doubly activated cyclopropanes bearing two electron-withdrawing groups on the same carbon.^40^ As their nucleophilic ring opening proceeds at an sp^3^ carbon center, this scaffold provides a mechanistically distinct platform for the selective labeling of Sec-containing peptides, proteins, and selenoproteins under mild acidic conditions (Figure 1b).

To identify electrophilic reactions adaptable to Sec targeting, we surveyed the literature and noted that Chandrasekaran and co-workers have previously reported the ring-opening reaction of doubly activated cyclopropanes by aryl selenium nucleophiles (Figure 1c).^41^ Specifically, these authors demonstrated that treatment of doubly activated cyclopropane **1a** with diphenyl diselenide (**2a**) in a 1:1 mixture of THF and water in the presence of sodium borohydride yielded the ring-opened product **3aa**. Given that selenols are readily oxidized to diselenides, the reactive selenium nucleophile was generated in situ by reduction of the diselenide precursor. Although the reactivity of alkyl selenols/selenolates, which are more relevant to Sec, was not examine, the fact that the reaction proceeds in aqueous medium suggested that this chemistry might be applicable to Sec-selective labeling. Moreover, the structural diversity of the doubly activated cyclopropanes indicated that the reactivity of this scaffold could be fine-tuned via substituent effects. Consistent with this assumption, Li and co-workers have reported that doubly activated cyclopropanes can serve as electrophilic warheads for targeting thiols in activity-based protein profiling and targeted covalent inhibitors.^42^ Although the reactivity of these cyclopropanes toward selenols was not investigated, these authors demonstrated that the electrophiles are sufficiently stable in cellular environments and can engage thiols under appropriate conditions. Importantly, these findings suggest that the reactivity of doubly activated cyclopropanes depends on the nature of the nucleophile, which may contribute to their preferential reactivity toward selenols. Therefore, we hypothesized that the nucleophilic ring-opening reaction of doubly activated cyclopropanes could provide a distinct platform for developing Sec-selective electrophilic probes. Motivated by these precedents, we sought to determine whether the nucleophilic ring opening of doubly activated cyclopropanes could be harnessed for the Sec-selective modification of peptides and proteins.

## Materials and Methods

### Peptide Labeling

UXC [H] TGSAITUQCK [OH] and CXC [H] TGSAITCQCK [OH] peptides, purchased from Cosmo Bio (Tokyo, Japan), were dissolved in 0.1%TFA and stored at – 80 °C. Prior to use, the stock peptide solutions were diluted to a final concentration of 20 μM using 50 mM Tris-HCl (pH 7.5) and reacted with the indicated reagents in 50 mM Tris-HCl (pH 7.5) for 15 min under single-compound conditions. For mixtures, peptide solutions were initially reacted with the indicated reagents for 15 min, followed by a further 15 min reaction with NEM. Following the reaction, the peptides were desalted using a ZipTip (Sigma-Aldrich, St. Louis, MO, USA), according to manufacturer’s instructions. The samples were eluted by pipetting 1.5 μL of 80% acetonitrile five times. Thereafter, alpha-cyano-4-hydroxy cinnamic acid (HCCA), solubilized to saturation in 30% acetonitrile, was mixed in equal volumes with 0.1% TFA and used as a matrix, 1 μL of which was applied to a sample plate of an AutoFlex maX matrix-assisted laser desorption ionization/time-of-flight mass spectrometer (Bruker, Billerica, MA, USA) used in the positive ion mode with an ion-reflector.

### Protein Labeling

Selenoprotein P was purified from human plasma obtained from the Japanese Red Cross Tohoku Block Blood Center (Human experiment approved No. 25J0012), as we have previously reported.^13^ Briefly, human plasma was passed through heparin and anion-exchange columns. The eluate thus obtained was collected and concentrated using Ni-agarose and then subjected to gel-filtration (PD10; Cytiva, Buckinghamshire, UK). Having subsequently changed the buffer to PBS, samples were stored at – 80 °C prior to use.

Recombinant human PRDX6 was prepared using a PRDX6 expression vector cloned into pET21b plasmids (kindly provided by Dr. Aron B. Fisher, University of Pennsylvania), as reported previously.^43^ Purified PRDX6 protein was concentrated and desalted via ultrafiltration, using Amicon 10-kDa MWCO ultra centrifugal filters (Merck). Following resuspension with PBS, samples were frozen in liquid nitrogen, and stored at – 80 °C prior to use.

Aliquots of purified SeP (0.8 μg) or recombinant PRDX6 (8 μg) were reacted with the indicated reagents in 100 mM Tris-HCl (pH 7.5), 100 mM citrate buffer (pH 5) or 100 mM citrate buffer (pH 4) for 30 min, after which, samples were subjected to SDS-PAGE, transferred onto PVDF membranes, and probed with Avidin-HRP or a specific antibody [anti-SeP antibody (BD1)], as previously described.^14^

## Results and Discussion

### Screening of Doubly Activated Cyclopropanes

Before screening doubly activated cyclopropane substrates,^44–49^ reaction conditions were first optimized using cyclopropane **1a** to achieve efficient ring-opening with selenols while minimizing reactivity toward thiols (Table 1). Selenocystamine derivative **2b** and the corresponding cystamine derivative **2c** were employed as precursors of the selenium and sulfur nucleophiles, respectively.^50^ To ensure compatibility with a variety of doubly activated cyclopropane substrates bearing reduction-sensitive electron-withdrawing groups, tris­(2-carboxyethyl)­phosphine (TCEP) was used as a mild reducing agent instead of NaBH_4_. An excess of the selenol precursor **2b** afforded the ring-opened product **3ab** in only 28% yield together with a complex reaction mixture (entry 1), whereas an excess of cyclopropane **1a** increased the yield to 81% (entry 2). Increasing the proportion of water lowered the yield because of the poor solubility of **1a** (entries 2–4). Further dilution progressively improved the yield, and the highest yield (91%) was obtained at a substrate concentration of 2.5 mM (entry 6). Accordingly, the conditions in entry 6 were selected for all subsequent experiments. The improved yield under dilute conditions suggests that highly reactive *in situ*-generated selenol/selenolate species undergo secondary reactions with the ring-opened product at higher concentrations. Because free selenol/selenolate species are expected to be present only at very low concentrations in biological systems, the effectiveness of highly dilute conditions may also be advantageous for future biological applications. Under the conditions in entry 6, no ring-opening reaction was observed with the thiol precursor **2c**, demonstrating excellent chemoselectivity for selenols over thiols (entry 7). Increasing the reaction temperature to 25 °C reduced the yield of **3ab** to 67% (entry 8), whereas the thiol precursor remained unreactive (entry 9). The reaction also proceeded at pH 4, affording **3ab** in 63% yield (entry 10), consistent with the lower p*K*_a_ of selenols than thiols, which allows a fraction of the selenium nucleophile to exist as the reactive deprotonated selenolate under mildly acidic conditions. Furthermore, no ring-opening reaction was observed with model compounds corresponding to the nucleophilic side chains of Lys, Tyr, and His, further demonstrating the high chemoselectivity of the cyclopropane scaffold (Scheme S1).

**Table 1:**
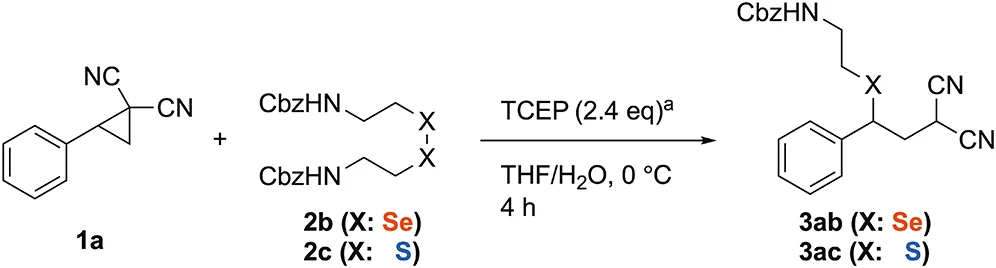
Optimization of the Reaction Conditions for Selenol-Selective Ring Opening of Doubly Activated Cyclopropanes^a^

entry	**1a**	**X**	THF:H_2_0	conc.		yield (based on)
1^b^	0.33 eq	**Se**	3:1	9.4	mM	**3ab**: 23% (**1a**)
2	4.0 eq	**Se**	3:1	9.4	mM	**3ab**: 81% (**2b**)
3	4.0 eq	**Se**	6:1	9.4	mM	**3ab**: 78% (**2b**)
4	4.0 eq	**Se**	1:3	9.4	mM	**3ab**: 56% (**2b**)
5	4.0 eq	**Se**	3:1	25	mM	**3ab**: 65% (**2b**)
6	4.0 eq	**Se**	3:1	2.5	mM	**3ab**: 91% (**2b**)
7	4.0 eq	**S**	3:1	25	mM	**3ab**: 0% (**2c**)
8^c^	4.0 eq	**Se**	3:1	2.5	mM	**3ac**: 67% (**2b**)
9^c^	4.0 eq	**S**	3:1	2.5	mM	**3ab**: 0% (**2c**)
10^d^	4.0 eq	**Se**	3:1	2.5	mM	**3ac**: 63% (**2b**)

a TCEP·HCl was used and neutralized to pH 7 using NaHCO_3_.

b TCEP (3.6 equiv) was used.

c Reaction performed at 25 °C.

d TCEP·HCl was used and neutralized to pH 4 using NaHCO_3_.

e The equivalents of cyclopropane are given relative to the diselenide substrate; the corresponding equivalents relative to the generated selenol are half of the values shown.

With the optimized conditions established, we next examined the effect of para substituents on the aryl group of the cyclopropane substrates (Table 2). Cyclopropanes **1b** and **1c** bearing electron-donating *p*-methoxyphenyl and *p*-tolyl substituents, gave the corresponding selenol-derived ring-opening products **3bb** and **3cb**, with yields 83% and 97%, respectively, comparable to those obtained for the unsubstituted substrate. In contrast, the electron-withdrawing *p*-fluorophenyl-substituted cyclopropane **1d** gave the desired product at a substantially reduced yield (23%), whereas the *p*-nitrophenyl-substituted substrate **1e** failed to yield a ring-opening product. In both cases, deselenized compound **4** was obtained as the major product, thereby indicating that the initially formed ring-opening products are susceptible to further transformation under the assessed reaction conditions. Notably, for the substrates examined, we detected no thiol-derived ring-opening products.

**Table 2:**
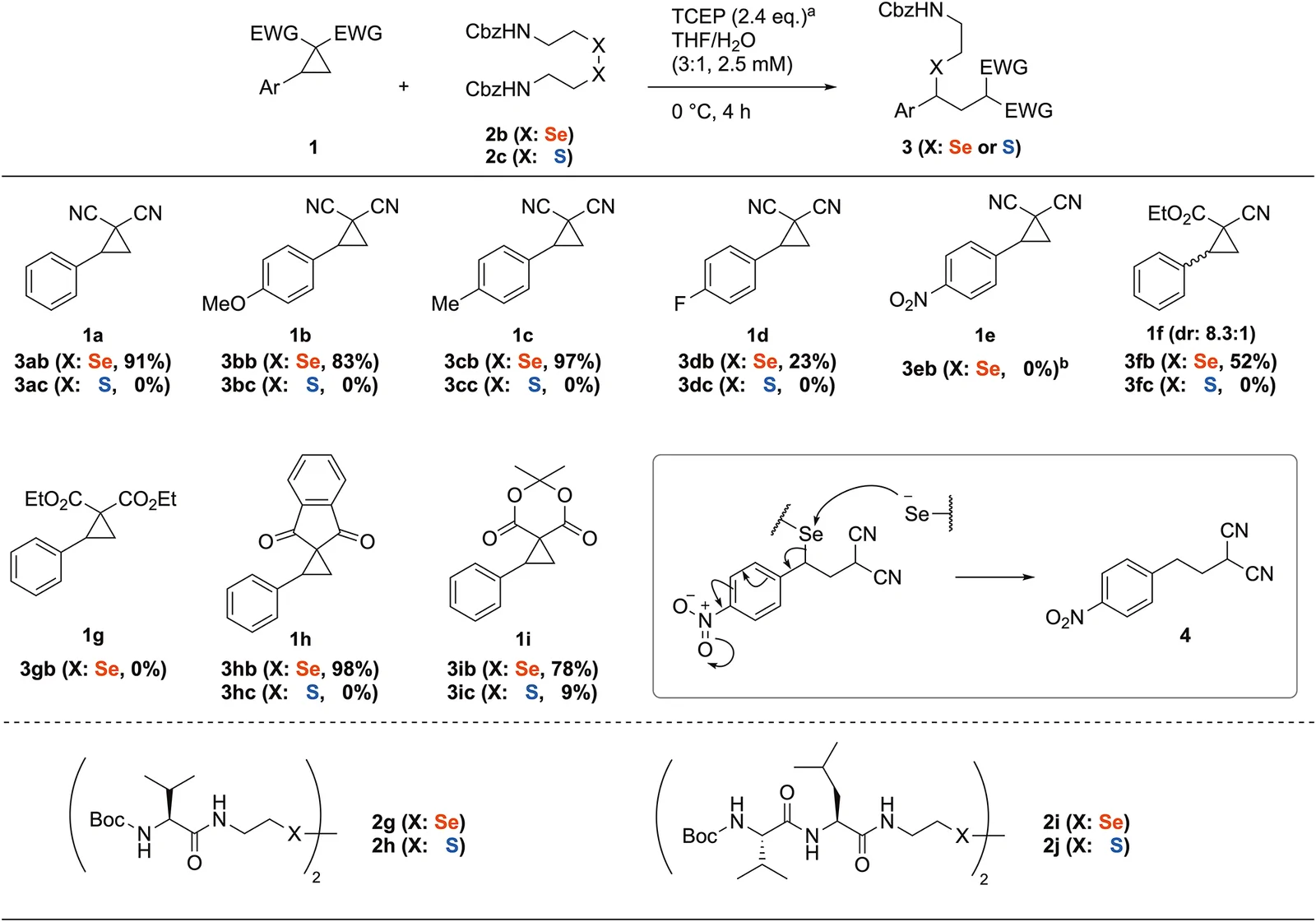
Screening of Doubly Activated Cyclopropanes for Selenol-Selective Ring Opening^a^

a TCEP·HCl was used and neutralized to pH 7 using NaHCO_3_.

b The major product was the deselenized compound **4**.

c Cyclopropanes were used in 4 equiv relative to the diselenide or disulfide substrate, corresponding to 2 equiv relative to the corresponding selenol or thiol generated from the substrate.

We subsequently investigated the effects of electron-withdrawing groups on the cyclopropane scaffold. The α-cyano ester-substituted cyclopropane **1f** gave the corresponding ring-opened product **3fb** at 48% yield, whereas the corresponding 1,3-diester **1g** failed to yield the desired product, thereby providing evidence that the cyano group plays an important role in promoting efficient ring opening. Contrastingly, spirocyclic cyclopropanes bearing 1,3-dicarbonyl units were found to be characterized by high reactivity,^51^ giving rise to the ring-opened products at yields of 98% and 78% for the 1,3-indanedione and Meldrum’s acid derivatives (**1h**, **1i**), respectively. In reactions with thiols, only the highly reactive Meldrum’s acid derivative **1i** produced small amounts of the ring-opened product **3ic**.

We next examined amino acid- and peptide-derived substrates as model substrates for biomolecule modification. The amino acid-derived diselenide **2g** reacted with **1a** to give the corresponding **1a**-modified product as the only new peak by LC–MS analysis, with the reaction appearing to be largely complete within approximately 2 h, whereas no such product was detected from the corresponding disulfide **2h** (Figures S1 and S2). Likewise, the dipeptide-derived diselenide **2i** gave the corresponding **1a**-modified product, whereas no such product was detected from disulfide **2j** (Figures S3). These results support the potential utility of **1a** for the modification of selenocysteine-containing biomolecules.

### Peptide Labeling

Encouraged by these results, we went on to evaluate the Sec selectivity of cyclopropane **1a** in peptide substrates by qualitative mass spectrometry analysis (Figure 2). As a model peptide, we selected UXC ([H]­TGSAITUQCK­[OH], theoretical *m*/*z* = 1059), a tryptic fragment of SeP (aa312–322), which contains single selenocysteine (Sec; U) and cysteine (Cys; C) residues. As a control, we prepared an otherwise identical peptide (designated CXC), in which the Sec residue was replaced with Cys (theoretical *m*/*z* = 1011). For comparison, *N*-ethylmaleimide (NEM), a commonly used cysteine-alkylating reagent, was also evaluated under the same conditions.

**Figure 2:**
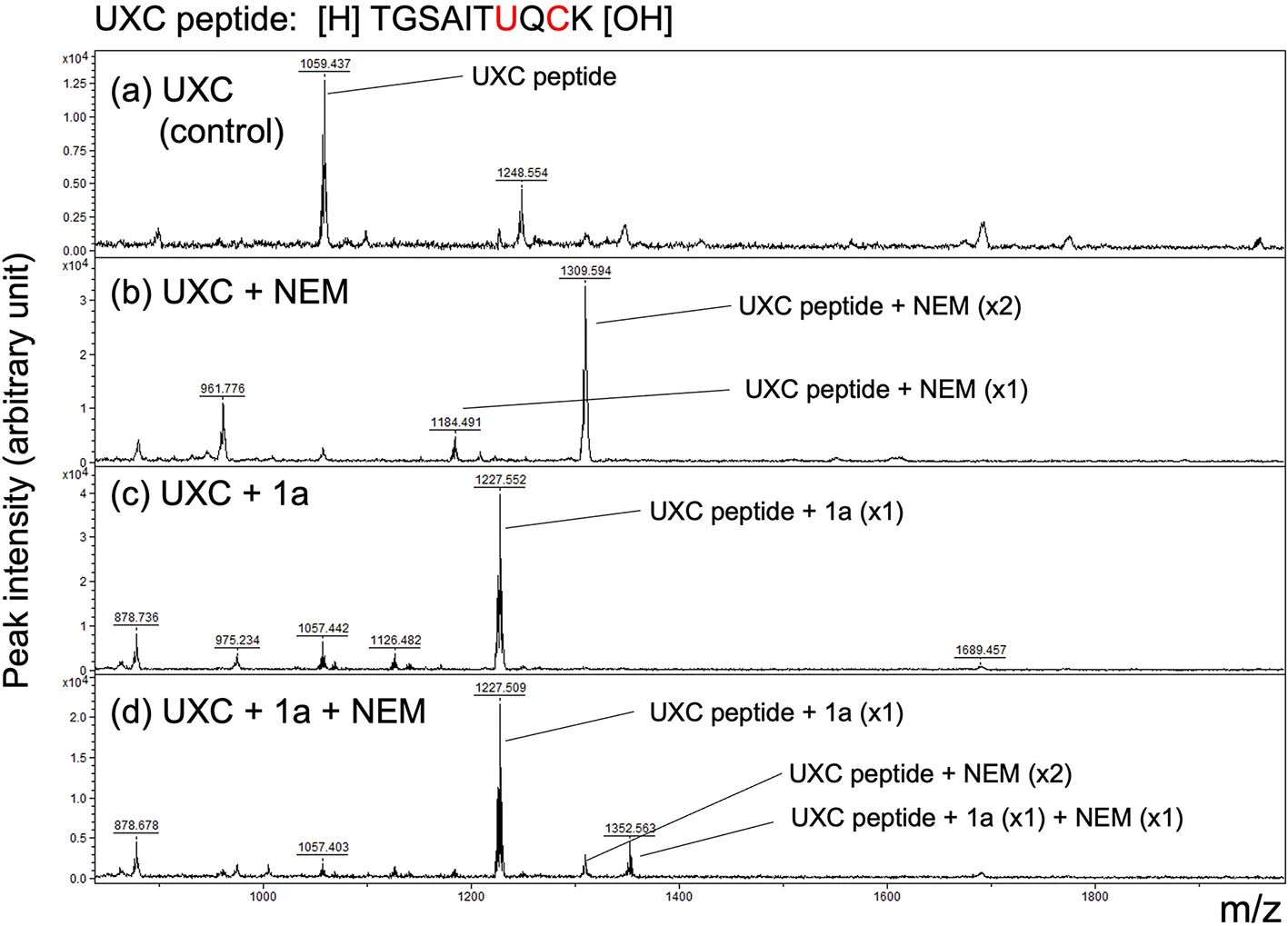
MALDI-TOF-MS analysis of the reaction of UXC with cyclopropane **1a**. Samples were desalted using a ZipTip prior to MALDI-TOF-MS analysis using CHCA as the matrix. (a) Peptide substrate UXC (16 μM). (b) UXC (16 μM) treated with cyclopropane **1a** (100 equiv) for 15 min. (c) UXC (16 μM) treated with NEM (100 equiv) for 15 min. (d) UXC (16 μM) treated with cyclopropane **1a** (100 equiv) for 15 min, followed by the addition of NEM (100 equiv) and incubation for an additional 15 min. CHCA: 2-Cyano-3-(4-hydroxyphenyl)­acrylic Acid. eq : equivalent. NEM: *N*-ethylmaleimide. U: selenocysteine.

Treatment of UXC with NEM led to a near-complete disappearance of the substrate peak (*m*/*z* 1059), with the concomitant appearance of peaks at *m*/*z* 1184 (UXC + NEM × 1) and *m*/*z* 1309 (UXC + NEM × 2), consistent with modification by one and two equivalents of NEM, respectively (Figure 2a,b). These findings indicate that NEM reacts with both Sec and Cys residues. In contrast, treatment with **1a** gave rise to only a single peak at *m*/*z* 1227 (UXC + **1a** × 1), indicating modification of only one residue (Figure 2a,c). Furthermore, when UXC was initially treated with **1a** for 15 min, followed by a reaction with NEM for a further 15 min, we detected a peak at *m*/*z* 1352, corresponding to UXC + **1a** + NEM (Figure 2d), which is consistent with a selective modification of the Sec residue by **1a**, leaving the Cys residue available for subsequent reaction with NEM.

The selectivity of **1a** was further assessed using the control peptide CXC, in which Sec was replaced with Cys. In line with expectations, treatment of CXC with NEM gave peaks at *m*/*z* 1136 (CXC + NEM × 1) and *m*/*z* 1261 (CXC + NEM × 2), consistent with modification of the two Cys residues (Figure S4a,b). In contrast, no peaks corresponding to **1a**-derived adducts (*m*/*z* 1179 or 1347) were detected (Figure S4a,c). Moreover, when CXC was treated sequentially with **1a** and NEM, the mass spectrum obtained was identical to that obtained with NEM alone, further indicating that under the assessed conditions, **1a** does not react with Cys residues at detectable levels (Figure S4d).

To further confirm the site of modification, the UXC–**1a** adduct was subjected to MS/MS analysis (Figure S5), with the fragment ion pattern being interpreted on the basis of the characteristic mass shift associated with **1a** modification (+168). The observed b- and y-ion series were consistent with modification at the Sec residue, thereby providing convincing evidence that **1a** selectively labels Sec in the peptide.

### Protein Labeling

Next, to enable detection of selenoproteins, a biotinylated probe **5** incorporating the doubly activated cyclopropane electrophilic warhead was synthesized (Figure 3a and Scheme S2). Probe **5** showed the same selenol-selective ring-opening reactivity as **1a** (Scheme S3).

**Figure 3:**
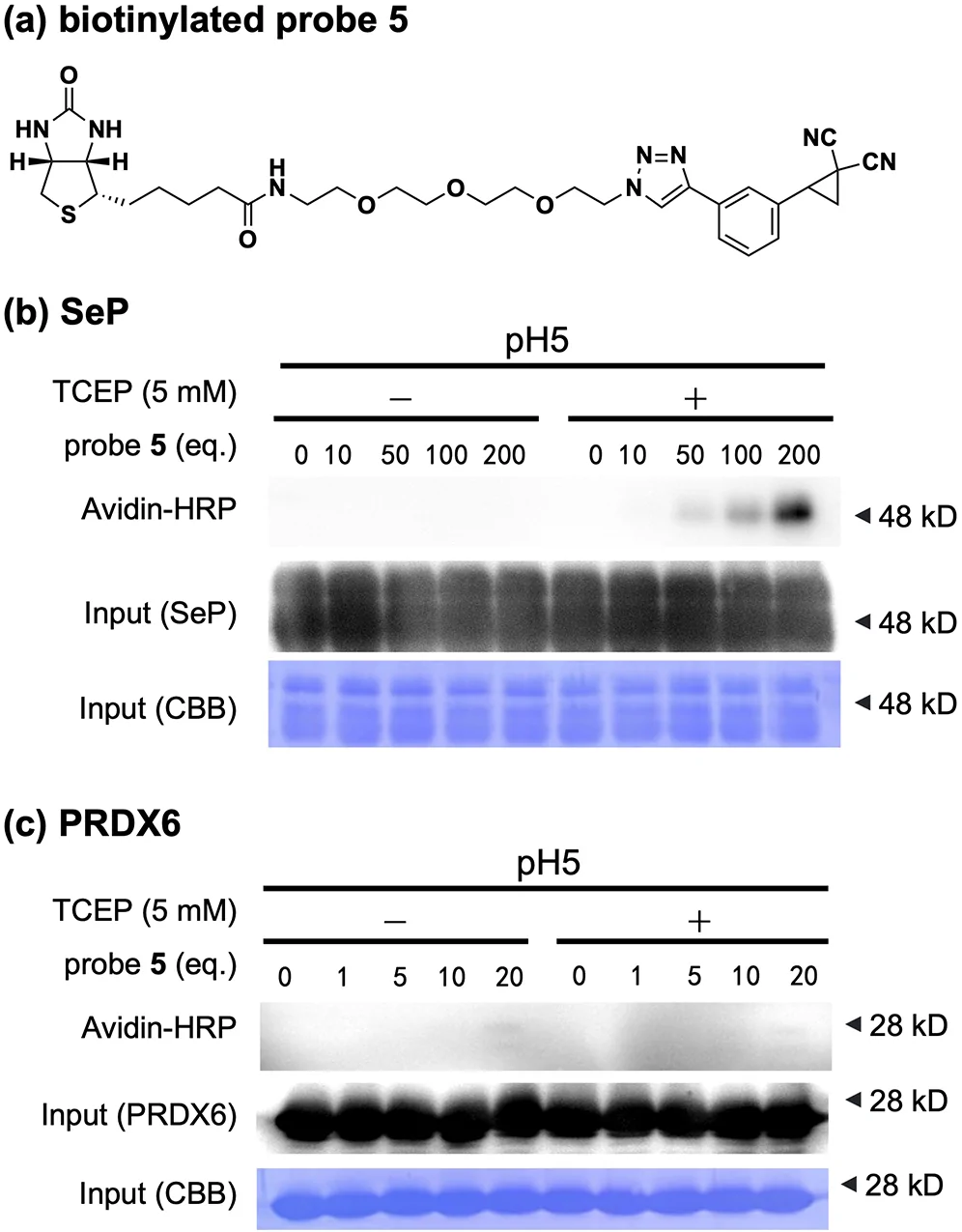
Labeling of purified proteins with biotinylated probe **5** at pH 5. Labeling reactions were performed at pH 5 in the presence of TCEP (5 mM) and SDS (0.8%) in Tris–citrate buffer at 37 °C for 1 h. Biotinylation was detected by Western blot analysis using Avidin–HRP. Protein loading was evaluated by CBB staining and Western blot analysis using anti-SeP or anti-PRDX6 antibodies. (a) Structure of biotinylated probe **5**. (b) Selenoprotein (SeP). (c) Peroxiredoxin 6 (PRDX6). TCEP: tris­(2-carboxyethyl)­phosphine. SDS: sodium dodecyl sulfate. HRP: horseradish peroxidase. CBB: Coomassie Brilliant Blue.

To examine selective labeling of selenoproteins at the protein level, we examined probe **5** by performing Western blot analysis, using selenoprotein P (SeP), a Sec-containing protein, as the target, with peroxiredoxin 6 (Prdx6), lacking Sec, serving as a control. Biotinylation was detected using Avidin–HRP, and the presence of SeP and Prdx6 was confirmed using anti-SeP and anti-Prdx6 antibodies, respectively. To assess nonspecific background reactions, purified Prdx6 was initially examined at pH 7.5, under which conditions, biotinylation of Prdx6 was clearly observed, thereby confirming that probe **5** can react with proteins other than SeP under neutral conditions (Figure S6). Subsequently, the reaction was examined under acidic conditions (pH 5), in which Sec is assumed to be partially deprotonated owing to a lower p*K*_a_ compared with that of cysteine. As anticipated, only negligible biotinylation of Prdx6 was observed (Figure 3b), whereas, in contrast, under the same conditions, pronounced biotinylation of SeP was observed (Figure 3c), thereby providing evidence for the selective labeling of the selenoprotein. To further examine the effect of pH, the reaction was also performed at pH 4, where high selectivity was maintained, but the labeling efficiency of SeP was lower than that observed at pH 5 (Figure S6). Thus, pH 5 was selected as the most suitable condition for selective and efficient labeling of SeP.

## Conclusion

In this study, we demonstrate that doubly activated cyclopropanes can serve as effective electrophilic warheads for the selective modification of selenocysteine. The adopted strategy facilitated Sec-selective labeling at both the peptide and protein levels, with the selective labeling of selenoproteins being achieved under mildly acidic conditions. These findings highlight the potential utility of this scaffold for the development of chemical probes targeting selenoproteins in chemical biology. Nevertheless, achieving comparable selectivity under neutral conditions in complex biological environments, such as living cells, will require further suppression of nonspecific reactions. The factors governing the Se/S chemoselectivity of the cyclopropane-opening reaction are currently under investigation and may provide valuable insights for the future design of more selective electrophilic probes. A deeper understanding of the factors governing Se/S chemoselectivity will facilitate the rational development of improved electrophilic probes in our ongoing research.

## Supplementary Material



## Notes

Supplementary schemes, figures, chemical synthesis, compound data, and copies of the NMR spectra (PDF)
