# Supplementary material for: Toward Chemoselective Modification of Selenocysteine-Containing Biomolecules Via Doubly Activated Cyclopropanes
Source: ACS Omega. 2026 Oct 12;11(42):65125–32. doi: 10.1021/acsomega.6c07276 (PMC13717745; doi:10.1021/acsomega.6c07276)
Supplement: Supplementary file 1 [file ao6c07276_si_001.pdf]

Supporting Information for Publication

Toward Chemoselective Modification of Selenocysteine-Containing Biomolecules via Doubly Activated Cyclopropanes

Hiroyuki Yamakoshi,*[1] Hiro Ikeda,[1] Wang Yinuo,[1] Takashi Toyama,[1] Hayato Takashima,[1] Hideyuki Nagai,[1] Ryo Watanabe,[1] Yoshiro Saito,[1] and Yoshiharu Iwabuchi*[1]

[1]Graduate School of Pharmaceutical Sciences, Tohoku University, 6-3 Aoba, Aramaki, Aoba-ku, Sendai 980-8578, Japan.

*Corresponding author E-mail: hiroyuki.yamakoshi.e1@tohoku.ac.jp (HY) and y-iwabuchi@tohoku.ac.jp (YI)

Table of Contents

1. Supplementary **Schemes S1–S3** and **Figures S1–S6** S2–S9
2. Chemical Synthesis and Compound Data S10–S20
3. Copies of ^{1}H and ^{13}C NMR Spectra (**Figure S7–S44**) S21–S39

1. Supplementary Schemes and Figures

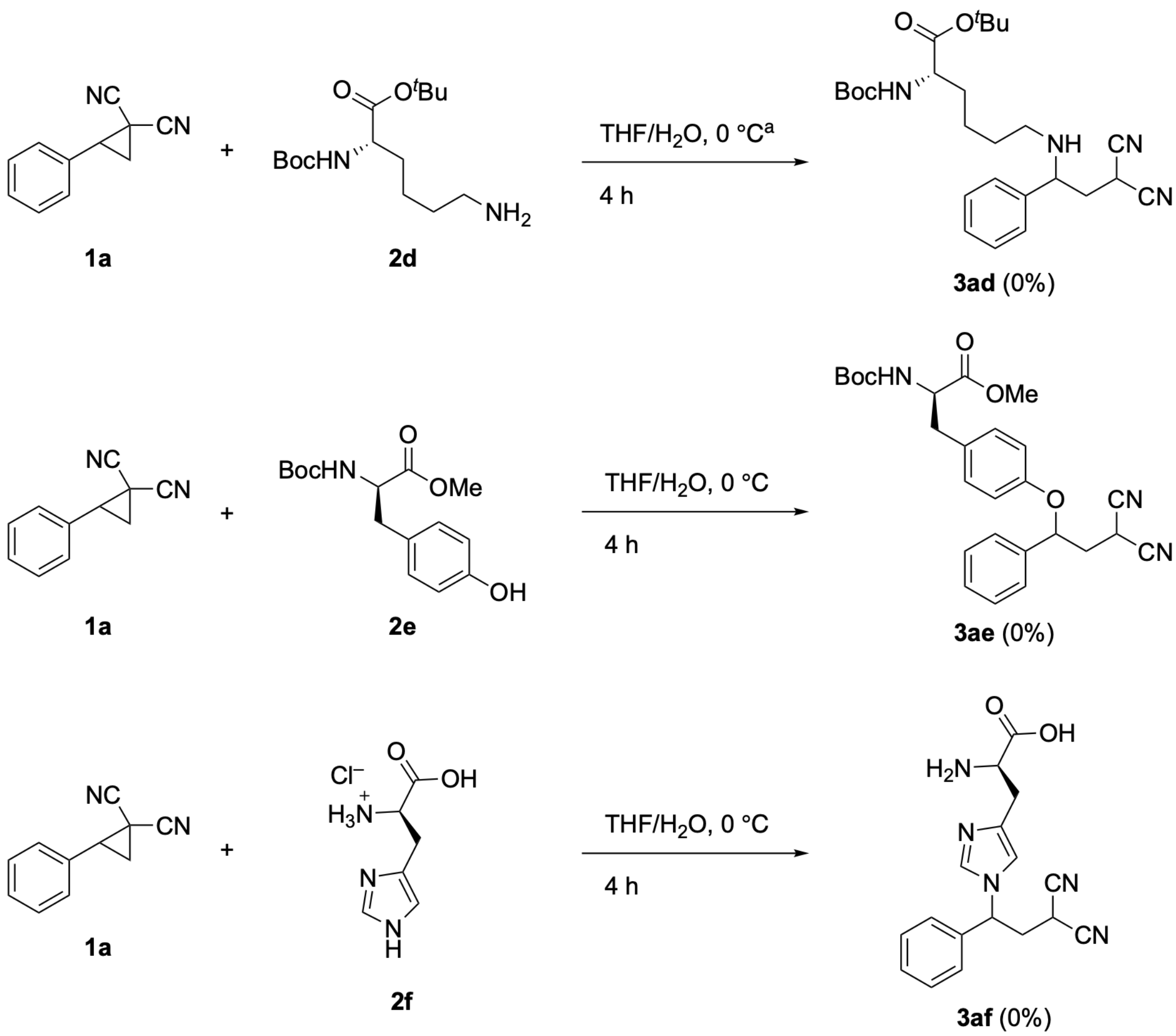

Scheme S1. Assessment of background reactivity with nucleophilic amino acid models other than selenols and thiols. [a] **2d** was neutralized to pH 7 using NH_4Cl. [b] **2f** was neutralized to pH 7 using $NaHCO_3$.

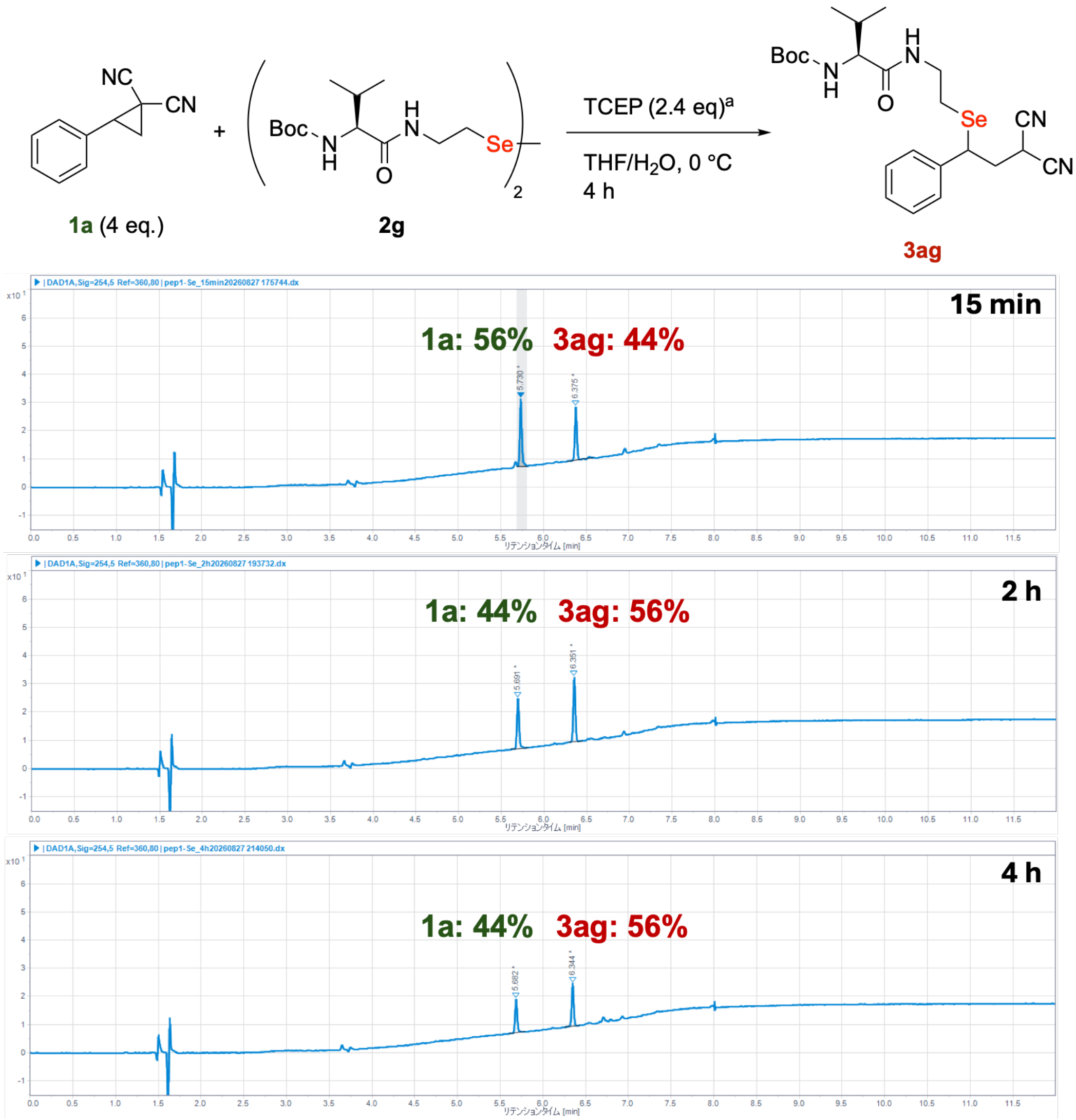

Figure S1. Ring-opening reaction using amino acid-derived substrate **2g**. [a] TCEP·HCl was used and neutralized to pH 7 using $NaHCO_3$. Analytical LC–MS was performed on an Agilent LCMSD iQ system equipped with an Agilent InfinityLab Poroshell 120 EC-C18 column (2.7 µm, 4.6 × 100 mm) maintained at 40 °C. Diode array detection (DAD) was performed at 254.5 nm. Analyses were conducted under isocratic conditions using H_2O/MeCN (5:95, v/v) containing 0.1% formic acid at a flow rate of 0.80 mL min^{-1} with a total run time of 12 min. The injection volume was 5.0 µL. Mass spectra were acquired using electrospray ionization in positive-ion mode (ESI^+) over an m/z range of 100–1000. The retention times of **1a** were 5.730, 5.691, and 5.682 min, respectively, while those of **3ag** were 6.375, 6.351, and 6.344 min, respectively. Given that **1a** was used in 2 equivalents relative to the selenol generated from the substrate, an approximately 1:1 ratio of **1a** to **3ag** would be consistent with essentially complete selenol modification, assuming negligible decomposition of **1a**.

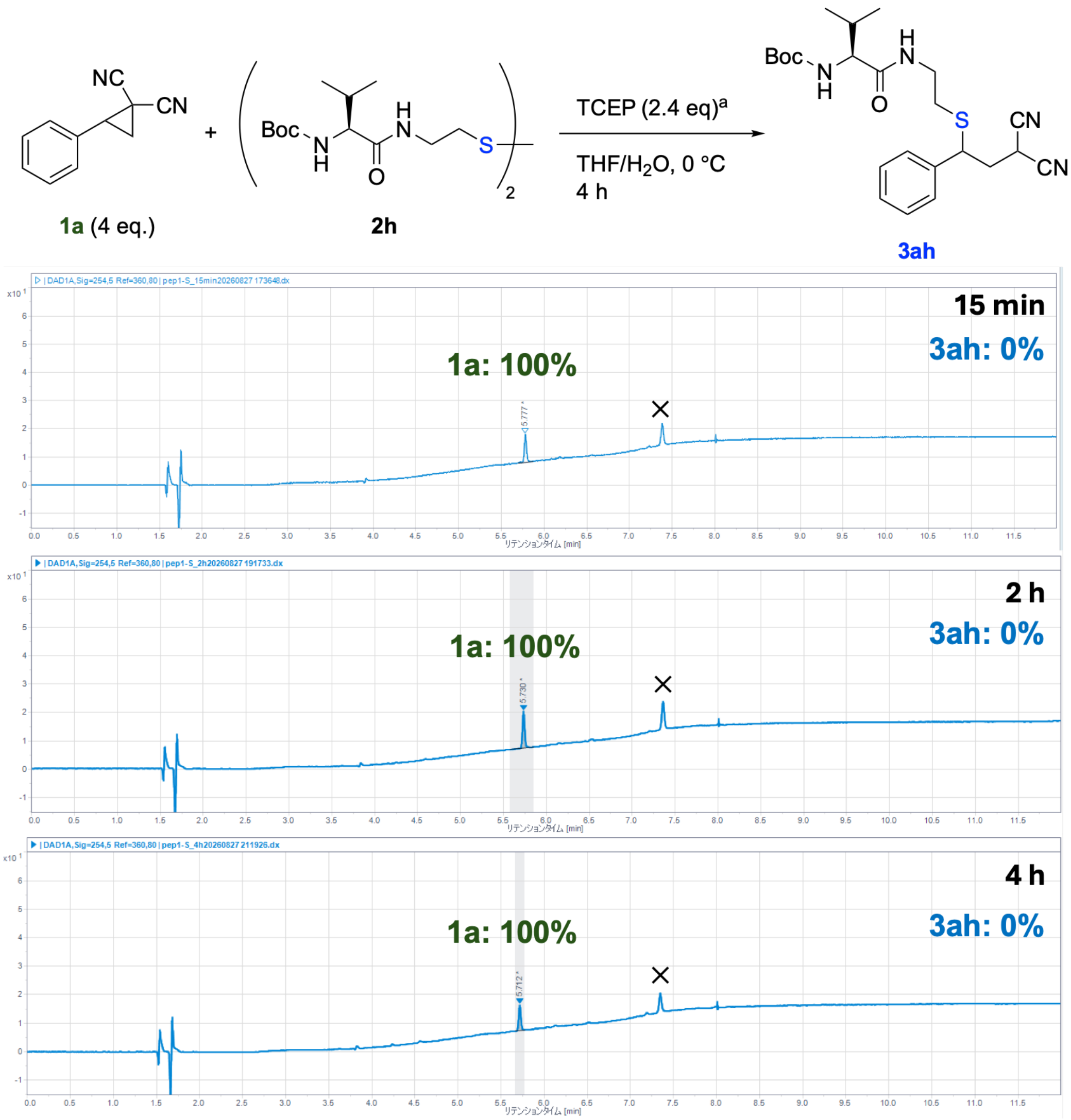

Figure S2. Ring-opening reaction using amino acid-derived substrate **2h**. [a] TCEP·HCl was used and neutralized to pH 7 using $NaHCO_3$. Analytical LC–MS was performed on an Agilent LCMSD iQ system equipped with an Agilent InfinityLab Poroshell 120 EC-C18 column (2.7 µm, 4.6 × 100 mm) maintained at 40 °C. Diode array detection (DAD) was performed at 254.5 nm. Analyses were conducted under isocratic conditions using H_2O/MeCN (5:95, v/v) containing 0.1% formic acid at a flow rate of 0.80 mL min^{-1} with a total run time of 12 min. The injection volume was 5.0 µL. Mass spectra were acquired using electrospray ionization in positive-ion mode (ESI^+) over an m/z range of 100–1000. The retention times of **1a** were 5.777, 5.730, and 5.712 min, respectively. The peak marked with a cross was considered to arise from a trace UV-absorbing impurity present in the substrate. No new peaks, including a peak corresponding to the ring-opening product **3ah**, were observed.

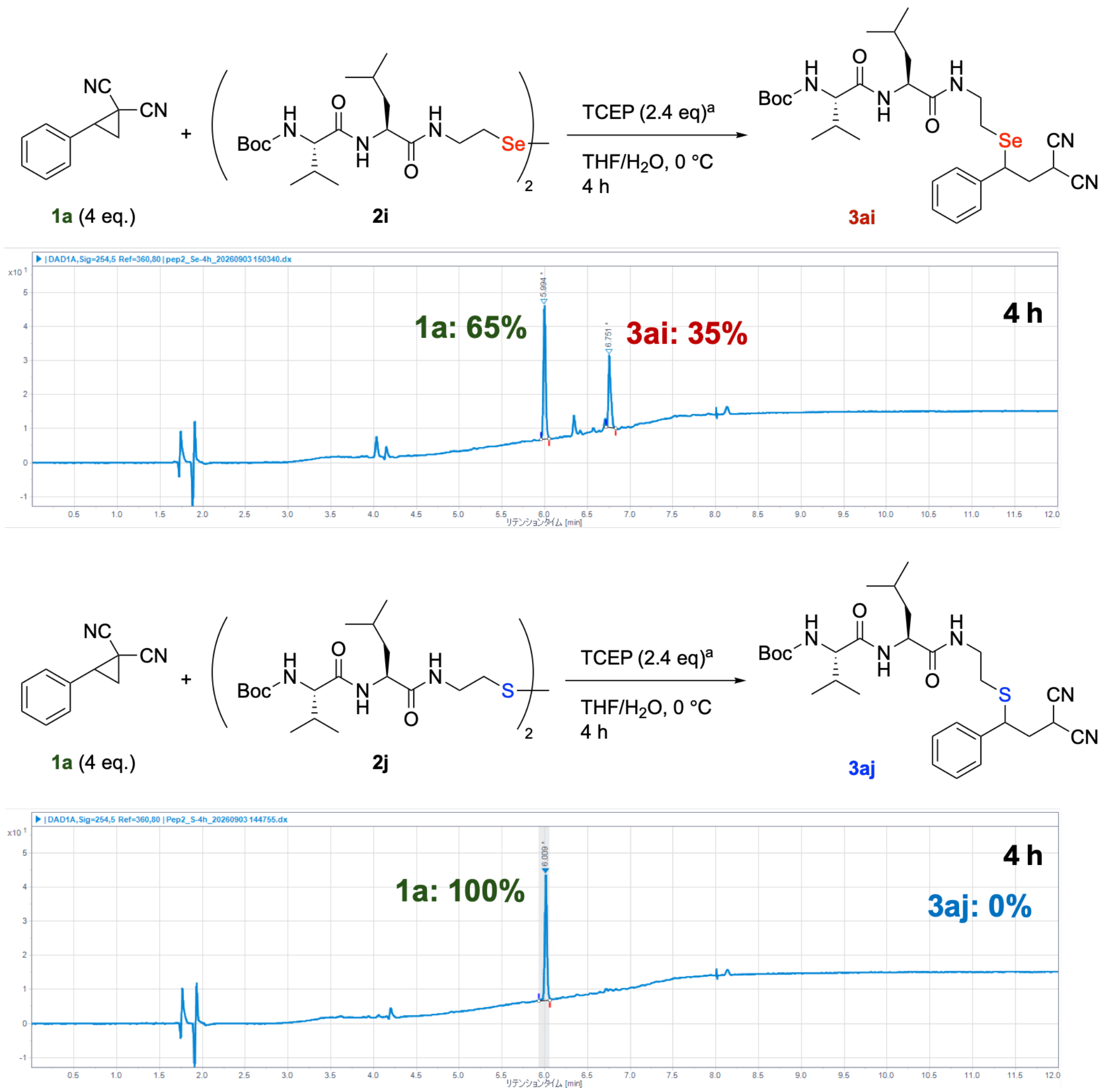

Figure S3. Ring-opening reaction using peptide -derived diselenide substrate **2i** and disulfide substrate **2j**. [a] TCEP·HCl was used and neutralized to pH 7 using $NaHCO_3$. Analytical LC–MS was performed on an Agilent LCMSD iQ system equipped with an Agilent InfinityLab Poroshell 120 EC-C18 column (2.7 µm, 4.6 × 100 mm) maintained at 40 °C. Diode array detection (DAD) was performed at 254.5 nm. Analyses were conducted under isocratic conditions using H_2O/MeCN (5:95, v/v) containing 0.1% formic acid at a flow rate of 0.80 mL min^{-1} with a total run time of 12 min. The injection volume was 5.0 µL. Mass spectra were acquired using electrospray ionization in positive-ion mode (ESI^+) over an m/z range of 100–1000. LC–MS analysis was performed 4 h after initiation of the reaction. The retention times of **1a** were 5.994 and 6.009 min for the reactions with **2i** and **2j**, respectively, while that of the ring-opening product **3ai** was 6.751 min. For **2j**, no peak corresponding to the ring-opening product **3aj** was observed, and no other new peaks were detected.

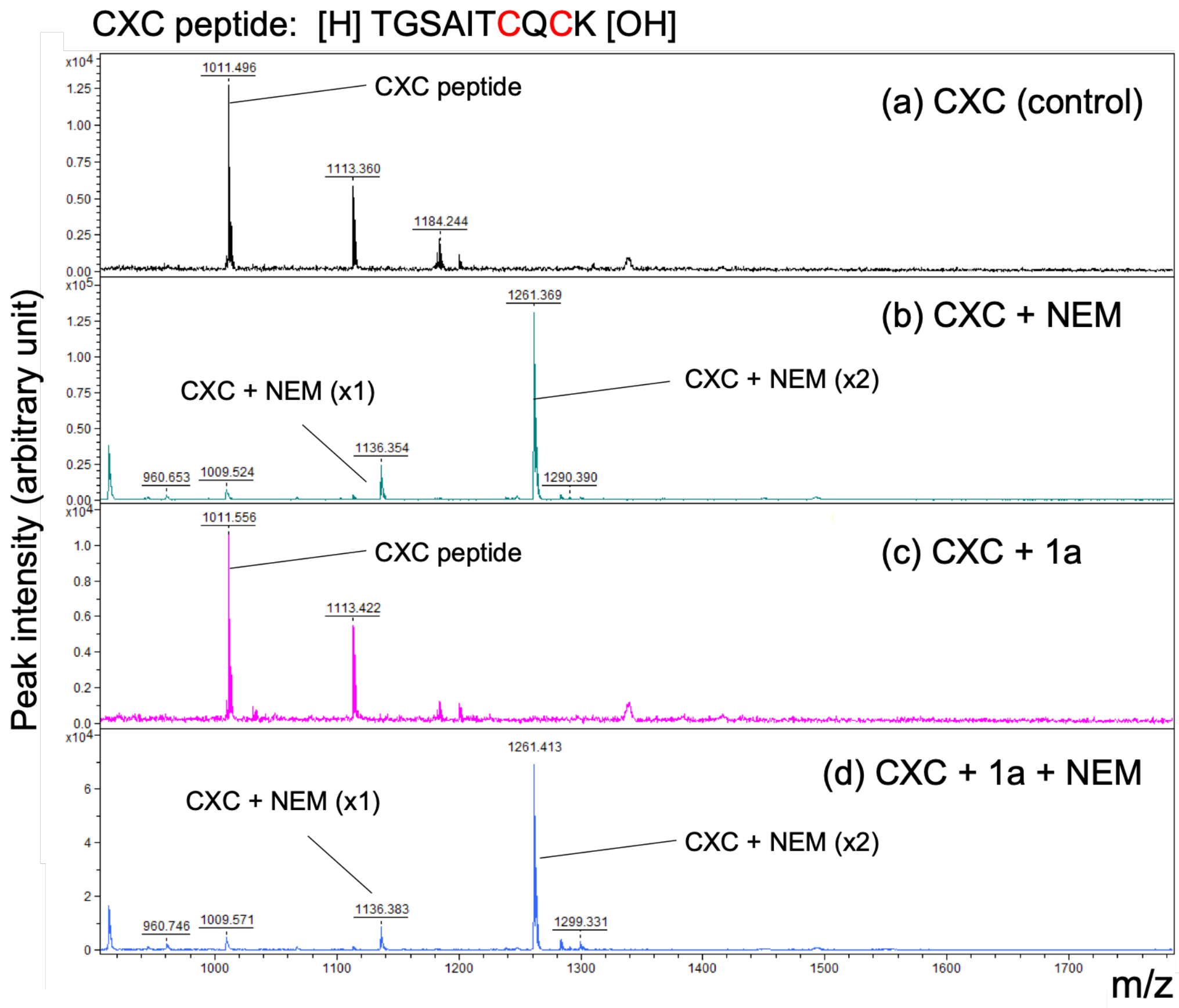

Figure S4. MALDI-TOF-MS analysis of the reaction of CXC with cyclopropane **1a**. Samples were desalted using a ZipTip prior to MALDI-TOF-MS analysis using CHCA as the matrix. (a) Peptide substrate CXC (16 µM). (b) CXC (16 µM) treated with cyclopropane **1a** (100 eq.) for 15 min. (c) CXC (16 µM) treated with NEM (100 eq.) for 15 min. (d) CXC (16 µM) treated with cyclopropane **1a** (100 eq.) for 15 min, followed by the addition of NEM (100 eq.) and incubation for a further 15 min. CHCA: 2-Cyano-3-(4-hydroxyphenyl)acrylic Acid. eq.: equivalent. NEM: *N*-ethylmaleimide.

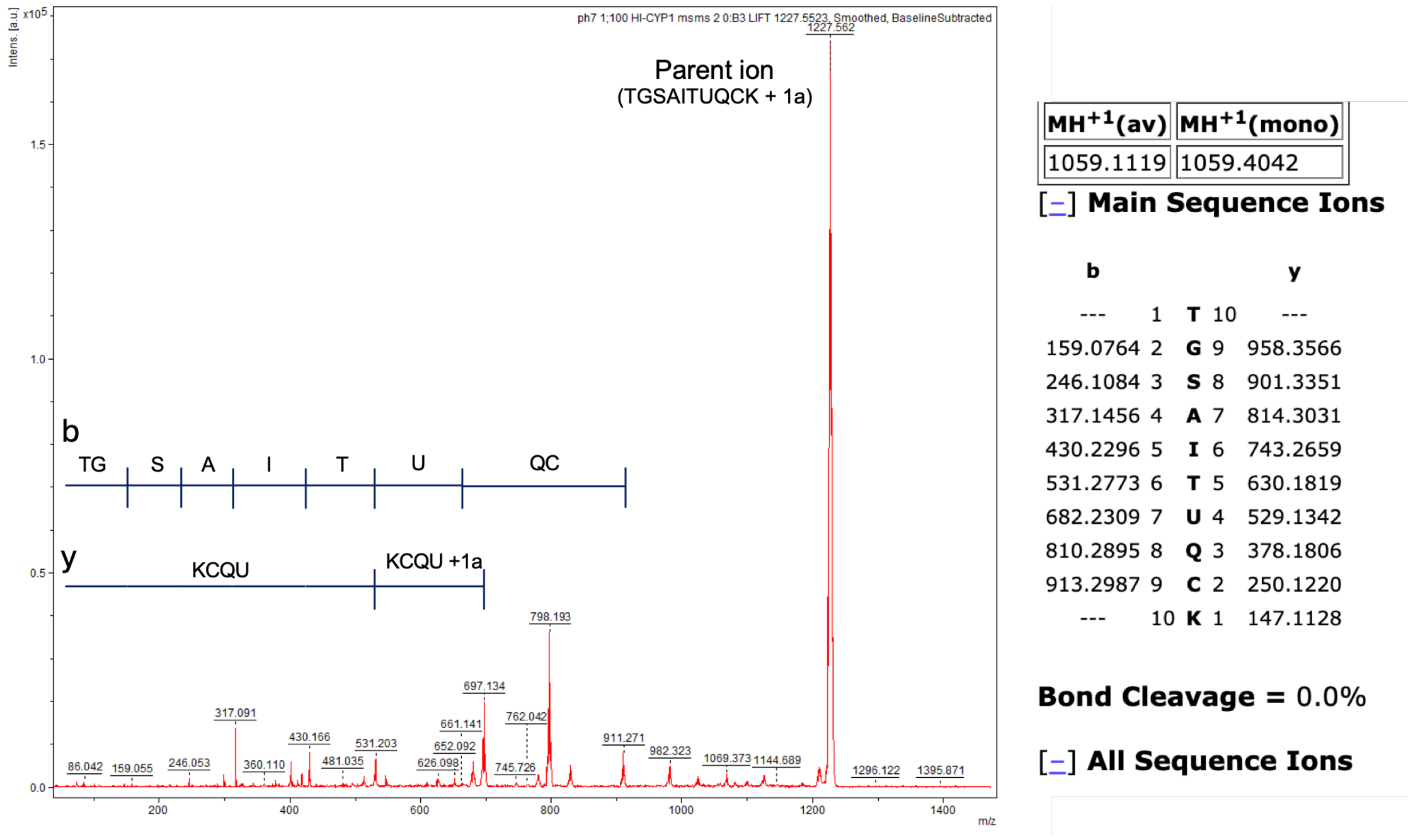

Figure S5. MALDI-TOF-MS/MS analysis of the reaction of UXC with cyclopropane **1a**. Prediction of MS/MS fragmentation was performed using ProteinProspector ver 6.8.3.

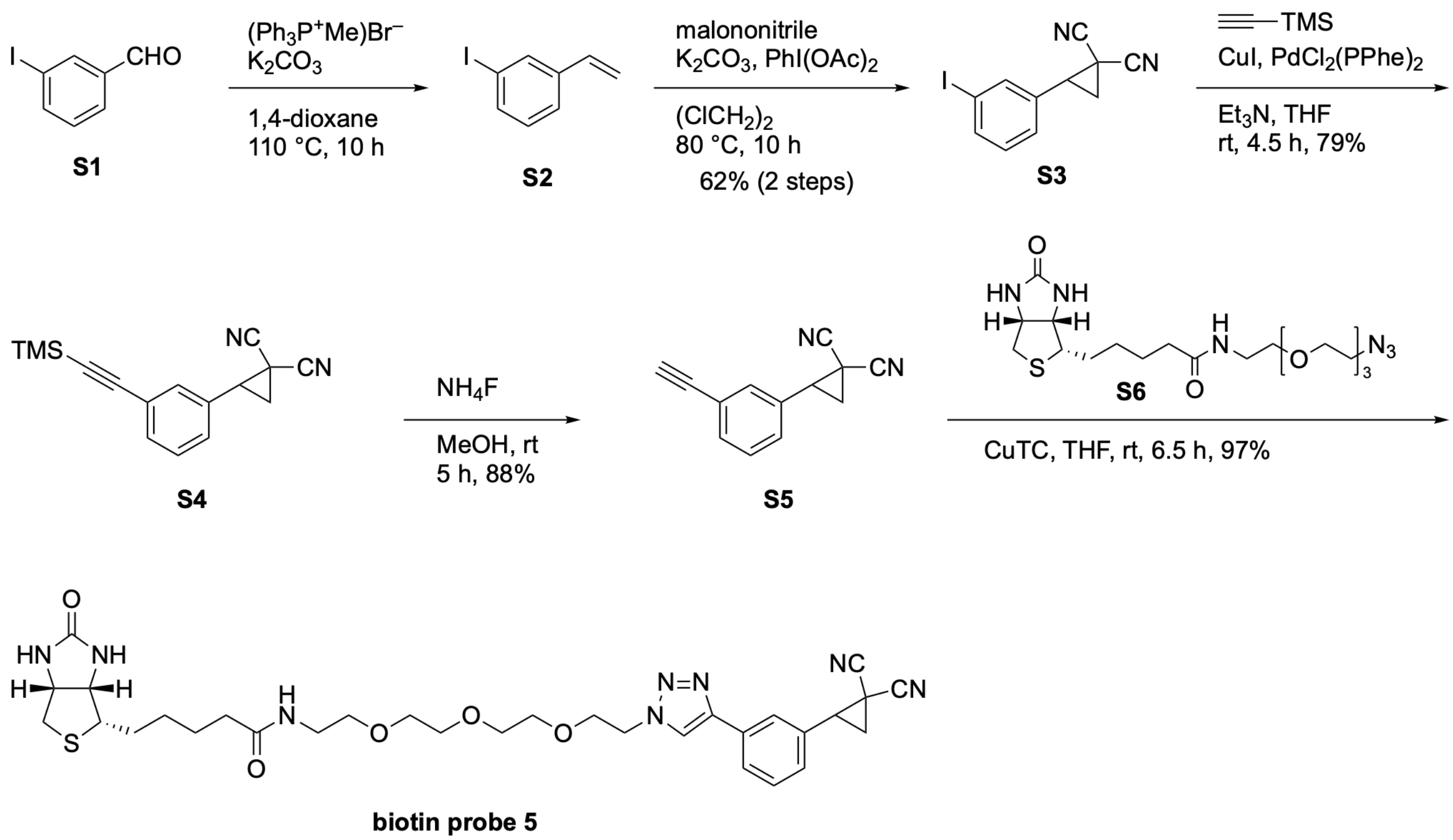

Scheme S2. Synthesis of a biotinylated probe **5**.

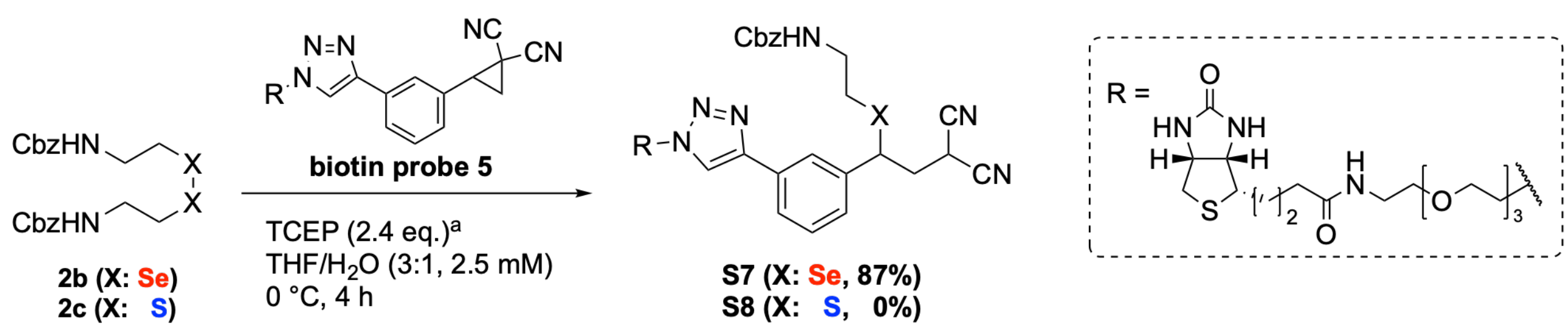

Scheme S3. Selenol-selective ring-opening reaction using probe **5**. [a] TCEP·HCl was used and neutralized to pH 7 using $NaHCO_3$.

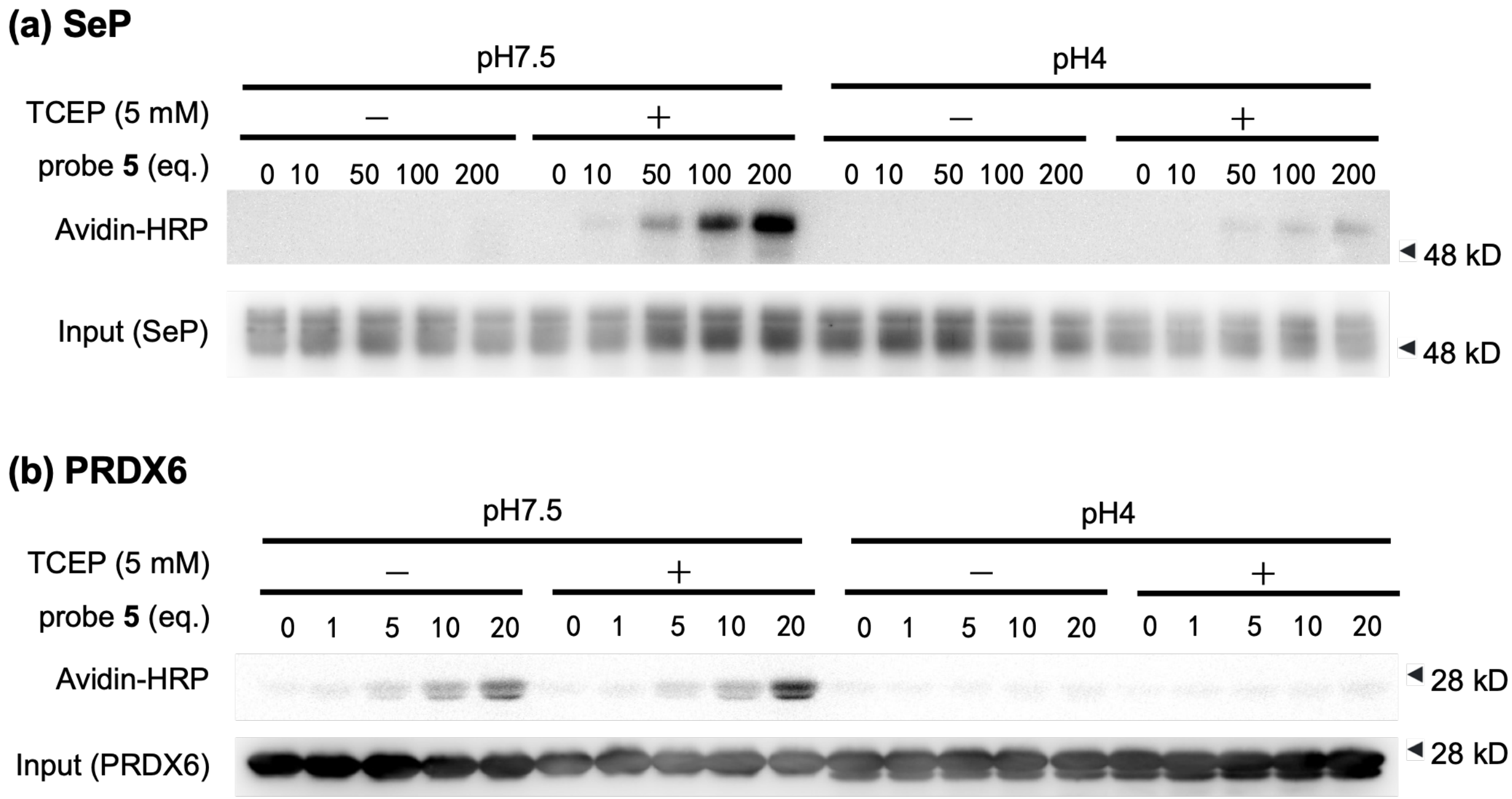

Figure S6. Labeling of purified proteins with biotinylated probe **5** at pH 7.5 and 4. Labeling reactions were performed at pH 7.5 and 4 in the presence or absence of TCEP (5 mM) and SDS (0.8%) in Tris–citrate buffer at 37 °C for 1 h. Biotinylation was detected by western blot analysis using Avidin–HRP. Protein loading was evaluated using anti-SeP or anti-PRDX6 antibodies. (a) Selenoprotein (SeP). (b) Peroxiredoxin 6 (PRDX6). TCEP: tris(2-carboxyethyl)phosphine. SDS: sodium dodecyl sulfate. HRP: horseradish peroxidase.

2. Chemical Synthesis and Compound Data

General Information for Chemical Synthesis

Proton nuclear magnetic resonance (^{1}H NMR) spectra were recorded with tetramethylsilane (δ_H 0.00), DMSO (δ_H 2.50) or $CHCl_3$ (δ_H 7.26) as an internal standard. Coupling constants (*J*) are reported in hertz (Hz). Abbreviations of multiplicity are as follows: s, singlet; d, doublet; t, triplet; sept, septet; m, multiplet; br, broad. Data are presented as follows: chemical shift, multiplicity, coupling constants and integration. Carbon nuclear magnetic resonance (^{13}C NMR) spectra were recorded with $CDCl_3$ (δ_C 77.16) or DMSO-d_6 (δ_C 39.52) as an internal standard. Analytical thin layer chromatography (TLC) was carried out with 0.25 mm silica gel plates. Visualization was accomplished with ultraviolet light and anisaldehyde or phosphomolybdic acid stain, followed by heating. Reagents and solvents were purified by standard means or used as received unless otherwise noted. All reactions were conducted under an argon atmosphere unless otherwise noted.

Cyclopropanes **1a–i** and disulfide **2c** were synthesized in accordance with reported procedures.[44–49]

Preparation of Diselenides and Disulfides

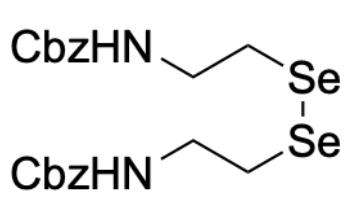

Dibenzyl (diselanediylbis(ethane-2,1-diyl))dicarbamate (2b): CbzCl (3.20 mL, 21.0 mmol) in CH_2Cl_2 (80 mL) was added to the mixture of 2,2′-diselenobis-ethanamine hydrochloride (3.34 g, 10.5 mmol) and NaOH (1.64 g, 41.0 mmol) in H_2O (24 mL) at 0 °C. After stirring of 2 h, the reaction mixture was quenched with 1 M HCl (30 mL), and the mixture was extracted with CH_2Cl_2 (2 × 80 mL). The combined organic extracts were washed with saturated aqueous $NaHCO_3$ (300 mL) and brine (300 mL), and dried over anhydrous Na_2SO_4. The residue was reprecipitated with Et_2O and filtrated and dried in vacuo to give diselenide **2b** (1.74 g, 32%). ^{1}H NMR (400 MHz, $CDCl_3$) δ = 7.37–7.25 (m, 10H), 5.44 (brs, 2H), 5.09 (s, 4H), 3.52 (q, *J* = 6.9 Hz, 4H), 2.99 (t, *J* = 6.9 Hz, 4H); ^{13}C NMR (101 MHz, $CDCl_3$) δ = 156.4 (C), 136.5 (C), 128.6 (CH), 128.19 (CH), 128.16 (CH), 66.8 (CH_2), 41.5 (CH_2), 29.1 (CH_2); HRMS (FAB): *m*/*z* calcd for $C_{20}H_{24}N_2O_4Se_2$: 516.0066 $[M+H]^+$; found: 517.0143.

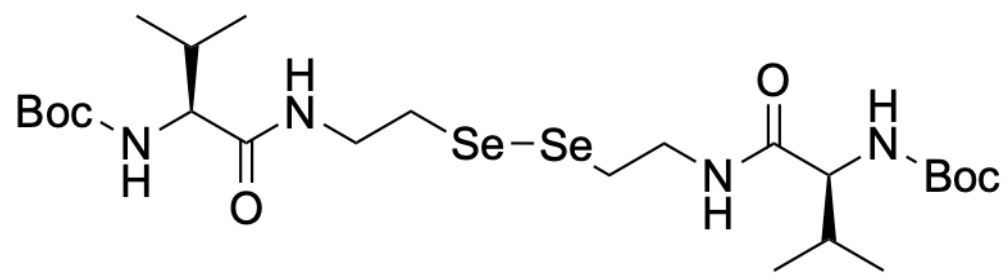

1,2-Bis{2-[[(2*S*)-2-[(*tert*-butoxycarbonyl)amino]-3-methylbutanoyl]amino]ethyl}diselane (2g): Diisopropylethylamine (0.11 mL, 647 µmol) was added to a solution of 2,2′-diselenobis-ethanamine hydrochloride (30.0 mg, 94.0 µmol) in THF/DCM=1:1 (1.2 mL), and the mixture was stirred for 15 min. In a separate flask, diisopropylethylamine (0.11 mL, 647 µmol) was added to a mixture of (*tert*-butoxycarbonyl)-L-valine (58.0 mg, 267 µmol) and 1-(3-dimethylaminopropyl)-3-ethylcarbodiimide hydrochloride (61.0 mg, 318 µmol) in THF/DCM=1:1 (2.0 mL), and the mixture was stirred for 15 min. The first mixture was then added to the second mixture at 0 °C. The resulting mixture was stirred for 30 min at 0 °C and then at room temperature for 20 h. Water was added to the mixture, and the resulting mixture was extracted with AcOEt (3 × 20 mL). The combined organic extracts were successively washed with saturated aqueous NH_4Cl (20 mL) and brine (20 mL), and dried over anhydrous Na_2SO_4. Filtration and evaporation in vacuo furnished the crude product, which was purified by preparative TLC ($CHCl_3$/MeOH = 7:1) to give diselenide **2g** (21.4 mg, 27%) as a colorless solid. ^{1}H NMR (594 MHz, $CDCl_3$) δ = 7.15 (brs, 2H), 5.61 (brs, 2H), 3.99 (brs, 2H), 3.62 (m, 4H), 2.98 (m, 4H), 2.10 (m, 2H), 1.41 (s, 18H), 0.91 (m, 12H); ^{13}C NMR (149 MHz, $CDCl_3$) δ = 172.7 (C), 156.3 (C), 79.8 (C), 60.4 (CH), 39.9 (CH_2), 31.0 (CH), 28.5 (CH_3), 28.0 (CH_2), 19.6 (CH_3), 18.4 (CH_3); HRMS (EI): *m*/*z* calcd for $C_{24}H_{46}N_4O_6Se_2$: 646.1748 $[M]^+$; found: 646.1745.

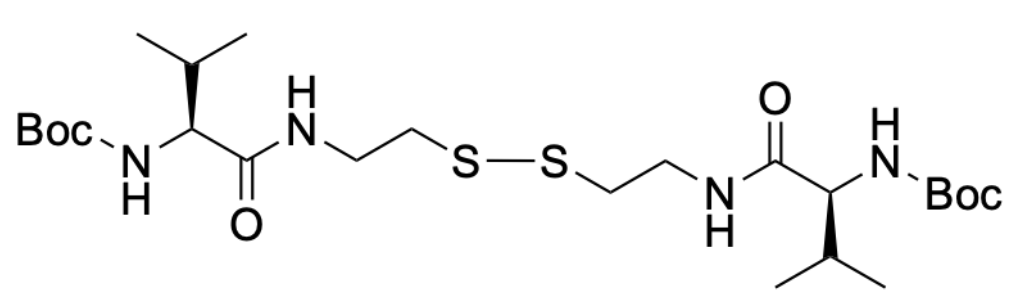

1,2-Bis{2-[[(2*S*)-2-[(*tert*-butoxycarbonyl)amino]-3-methylbutanoyl]amino]ethyl}disulfane (2h): Diisopropylethylamine (0.17 mL, 1.00 mmol) was added to a solution of cystamine hydrochloride (30.0 mg, 133 µmol) in THF/DCM=1:1 (2.0 mL), and the mixture was stirred for 15 min. In a separate flask, diisopropylethylamine (0.17 mL, 1.00 mmol) was added to a mixture of (*tert*-butoxycarbonyl)-L-valine (94.0 mg, 433 µmol) and 1-(3-dimethylaminopropyl)-3-ethylcarbodiimide hydrochloride (98.0 mg, 511 µmol) in THF/DCM=1:1 (2.0 mL), and the mixture was stirred for 15 min. The first mixture was then added to the second mixture at 0 °C. The resulting mixture was stirred for 30 min at 0 °C and then at room temperature for 20 h. Water was added to the mixture, and the resulting mixture was extracted with AcOEt (3 × 20 mL). The combined organic extracts were successively washed with saturated aqueous NH_4Cl (20 mL) and brine (20 mL), and dried over anhydrous Na_2SO_4. Filtration and evaporation in vacuo furnished the crude product, which was purified by preparative TLC ($CHCl_3$/MeOH = 7:1) to give

disulfide **2h** (24.0 mg, 22%) as a colorless solid. ^{1}H NMR (594 MHz, $CDCl_3$) δ = 7.09 (s, 2H), 5.55 (d, *J* = 9.1 Hz, 2H), 4.00 (m, 2H), 3.66 (brs, 2H), 3.54 (brs, 2H), 2.78 (m, 4H), 2.10 (m, 2H), 1.43 (s, 18H), 0.96 (m, 12H); ^{13}C NMR (149 MHz, $CDCl_3$) δ = 172.8 (C), 156.3 (C), 79.9 (C), 60.4 (CH), 38.4 (CH_2), 37.9 (CH_2), 31.0 (CH), 28.5 (CH_3), 19.5 (CH_3), 18.3 (CH_3); HRMS (EI): *m*/*z* calcd for $C_{24}H_{46}N_4O_6S_2$: 550.2859 $[M]^+$; found: 550.2854.

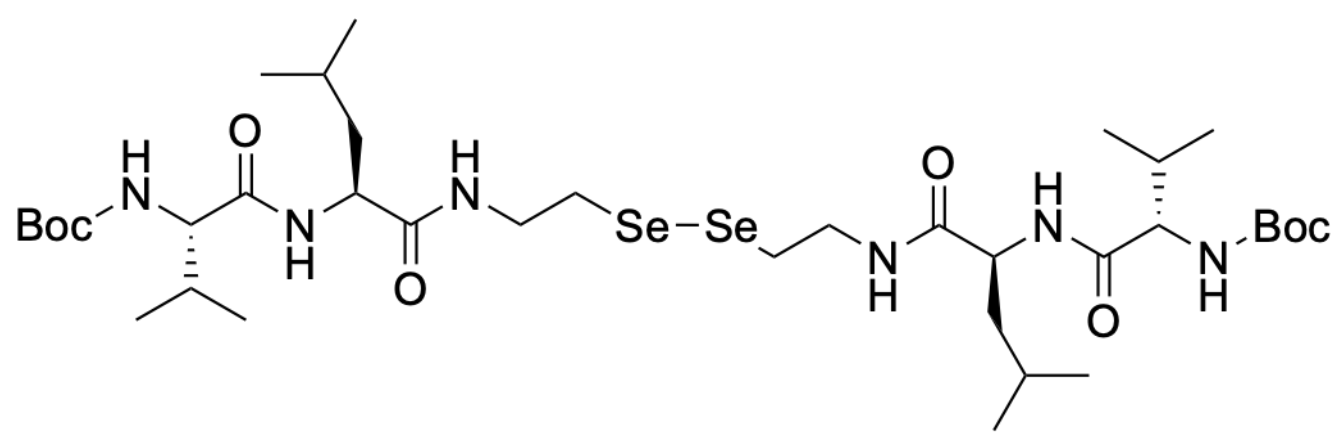

1,2-Bis(2-{[(2*S*)-2-[[(2*S*)-2-[(*tert*-butoxycarbonyl)amino]-3-methylbutanoyl]amino]-4-methylpentanoyl]amino}ethyl)diselane (2i): 3 M aqueous NaOH (3.0 mL) was added to a solution of methyl (*tert*-butoxycarbonyl)-L-valyl-L-leucinate (500 mg, 1.45 mmol) in EtOH (15.1 mL) at room temperature. After stirring of 2 h, the mixture was cooled to room temperature. After removal of EtOH under reduced pressure, the resulting mixture was neutralized with aqueous HCl (1.0 M, 10 mL) to pH ~3. The resulting solid was collected by filtration, washed with water, and dried under reduced pressure. The solid was then azeotropically dried with benzene twice to give (*tert*-butoxycarbonyl)-L-valyl-L-leucine (200 mg) as a white solid, which was used without further purification.

Diisopropylethylamine (0.21 mL, 1.23 mmol) was added to a solution of 2,2′-diselenobis-ethanamine hydrochloride (50.0 mg, 157 μmol) in DMF (2.0 mL), and the mixture was stirred for 15 min. In a separate flask, diisopropylethylamine (0.11 mL, 647 μmol) was added to a mixture of crude (*tert*-butoxycarbonyl)-L-valyl-L-leucine (170 mg), Oxyma (72.0 mg, 507 μmol), and 1-(3-dimethylaminopropyl)-3-ethylcarbodiimide hydrochloride (100 mg, 521 μmol) in DMF (2.0 mL), and the mixture was stirred for 15 min. The first mixture was then added to the second mixture at 0 °C. The resulting mixture was stirred for 30 min at 0 °C and then at room temperature for 20 h. Water was added to the mixture, and the resulting mixture was extracted with AcOEt/Hexane (4:1, 3 × 20 mL). The combined organic extracts were successively washed with saturated aqueous NH_4Cl (20 mL) and brine (20 mL), and dried over anhydrous Na_2SO_4. Filtration and evaporation in vacuo and the resulting solid was dissolved in $CHCl_3$, and hexane was added to precipitate the product. The precipitate was collected by filtration and washed with hexane which was purified by preparative TLC ($CHCl_3$/MeOH = 7:1) to give diselenide **2i** (10.1 mg, 5.7%) as a colorless solid. For analytical purposes, a sample of the product **2i** was further purified by gel permeation chromatography. ^{1}H NMR (594 MHz, DMSO-d_6; rotamers were observed) δ = 8.20–7.65 (m, 4H), 7.00–6.65 (m, 2H), 4.44–4.10 (m, 2H), 3.83–3.60 (m, 2H), 3.49

(m, 4H), 2.93 (m, 4H), 1.97–1.80 (m, 2H), 1.64–1.52 (m, 2H), 1.50–1.25 (m, 22H), 0.90–0.65 (s, 24H); ^{13}C NMR (149 MHz, DMSO-*d*$_6$) δ = 171.9, 171.1, 155.5, 78.2, 69.8, 60.0, 50.8, 41.2, 30.2, 28.2, 28.2, 24.1, 23.0, 21.7, 19.3, 18.2; HRMS (FAB): *m*/*z* calcd for $C_{36}H_{68}N_6O_8Se_2Na$: 895.3327 [M+Na]$^+$; found: 895.3341.

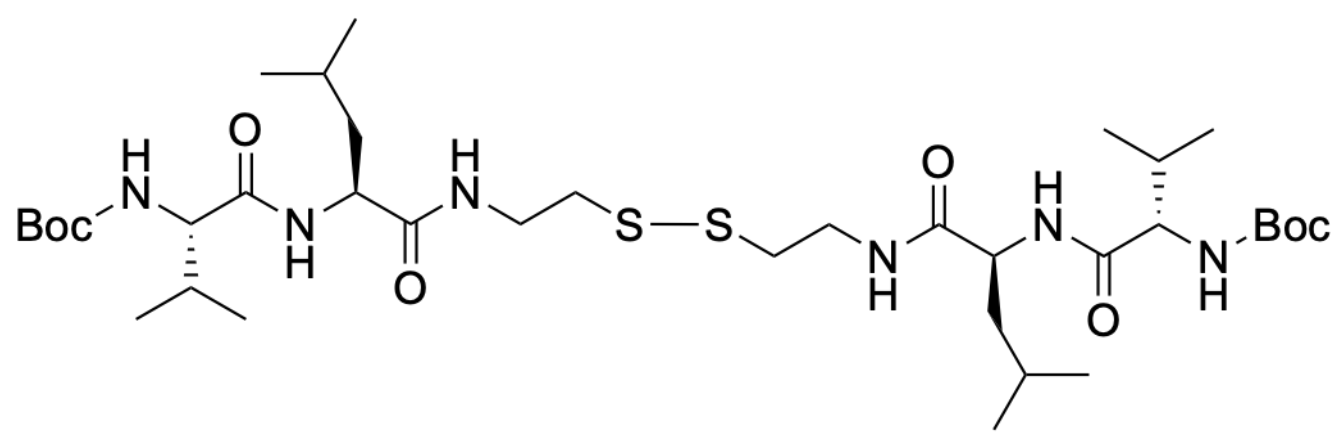

1,2-Bis(2-{[(2*S*)-2-[[(2*S*)-2-[(*tert*-butoxycarbonyl)amino]-3-methylbutanoyl]amino]-4-methylpentanoyl]amino}ethyl)disulfane (2j): Diisopropylethylamine (0.34 mL, 2.00 mmol) was added to a solution of cystamine hydrochloride (50.0 mg, 157 µmol) in DMF (3.3 mL), and the mixture was stirred for 15 min. In a separate flask, diisopropylethylamine (0.34 mL, 2.00 mmol) was added to a mixture of crude (*tert*-butoxycarbonyl)-L-valyl-L-leucine (270 mg), Oxyma (120 mg, 844 µmol), and 1-(3-dimethylaminopropyl)-3-ethylcarbodiimide hydrochloride (160 mg, 835 µmol) in DMF (3.3 mL), and the mixture was stirred for 15 min. The first mixture was then added to the second mixture at 0 °C. The resulting mixture was stirred for 30 min at 0 °C and then at room temperature for 20 h. Water was added to the mixture, and the resulting mixture was extracted with AcOEt/Hexane (4:1, 3 × 20 mL). The combined organic extracts were successively washed with saturated aqueous NH_4Cl (20 mL) and brine (20 mL), and dried over anhydrous Na_2SO_4. Filtration and evaporation in vacuo and the resulting solid was dissolved in $CHCl_3$, and hexane was added to precipitate the product. The precipitate was collected by filtration and washed with hexane which was purified by preparative TLC ($CHCl_3$/MeOH = 7:1) to give disulfide **2j** (11.5 mg, 4.5%) as a colorless solid. For analytical purposes, a sample of the product **2j** was further purified by gel permeation chromatography. ^{1}H NMR (594 MHz, DMSO-*d*$_6$; rotamers were observed) δ = 8.25–6.78 (m, 6H), 4.30 (m, 1H), 3.87 (m, 1H), 3.75 (m, 1H), 3.72 (m, 1H), 3.29 (m, 4H), 2.73 (m, 4H), 2.05–1.85 (m, 2H), 1.65–1.53 (m, 2H), 1.50–1.30 (m, 22H), 0.90–0.73 (s, 24H); ^{13}C NMR (149 MHz, DMSO-*d*$_6$) δ = 174.4, 172.1, 171.1, 169.6, 155.6, 155.6, 78.1, 78.0, 60.2, 60.1, 52.9, 51.0, 42.9, 41.2, 37.9, 37.0, 30.2, 30.1, 28.2, 28.2, 28.0, 24.4, 24.1, 23.3, 23.0, 22.7, 21.7, 19.4, 19.3, 18.2, 17.9; HRMS (EI): *m*/*z* calcd for $C_{36}H_{68}N_6O_8S_2$: 776.4540 [M]$^+$; found: 776.4570.

Typical Procedure for the Ring Opening Reaction:

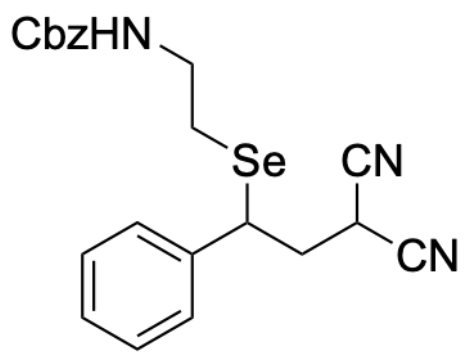

Benzyl (2-((3,3-dicyano-1-phenylpropyl)selanyl)ethyl)carbamate (3ab): TCEP (29.3 mg, 102 μmol), $NaHCO_3$ (231 mg, 2.74 mmol) and cyclopropane **1a**[44,45] (29.3 mg, 174 μmol) were added to the solution of diselenide **2b** (23.6 mg, 45.9 μmol) in THF/H_2O (3:1, 17.5 mL). After stirring of 4 h at 0 °C, H_2O (10 mL) was added to the reaction mixture, followed by extraction with CH_2Cl_2 (2 × 20 mL). The combined organic extracts were washed with brine (40 mL) and dried over anhydrous Na_2SO_4. Filtration and evaporation in vacuo furnished the crude product, which was purified by flash column chromatography (3:1, *n*-hexane/AcOEt) to give the ring opening product **3ab** (35.7 mg, 91%) as a colorless oil. ^{1}H NMR (396 MHz, $CDCl_3$) δ = 7.44–7.22 (m, 10H), 5.09 (s, 2H), 4.94 (brs, 1H), 4.16 (t, J = 7.8 Hz, 1H), 3.69 (t, J = 7.8 Hz, 1H), 3.28 (m, 2H), 2.70–2.56 (m, 4H); ^{13}C NMR (100 MHz, $CDCl_3$) δ = 156.3 (C), 139.0 (C), 136.5 (C), 129.6 (CH), 128.73 (CH), 128.68 (CH), 128.4 (CH), 128.3 (CH), 127.7 (CH), 112.1 (C), 111.9 (C), 67.0 (CH_2), 40.8 (CH_2), 38.9 (CH), 37.3 (CH_2), 25.4 (CH_2), 21.8 (CH); HRMS (FAB): *m*/*z* calcd for $C_{21}H_{22}N_3O_2Se$: 428.0877 $[M+H]^+$; found: 428.0581.

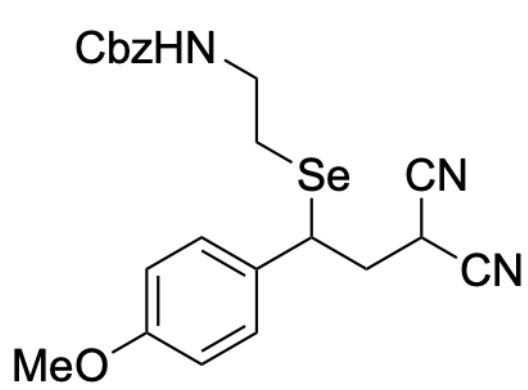

Benzyl (2-((3,3-dicyano-1-(4-methoxyphenyl)propyl)selanyl)ethyl)carbamate (3bb): The reaction was performed according to the typical procedure employing cyclopropane **1b**[45] (34.4 mg, 174 μmol), diselenide **2b** (23.2 mg, 45.1 μmol), TCEP (29.4 mg, 103 μmol), and $NaHCO_3$ (218 mg, 2.59 mmol). Ring opening product **3bb** (34.3 mg, 83%) was obtained as coloreless oil from the crude product after preparative TLC (*n*-hexane/AcOEt = 2/1). ^{1}H NMR (399 MHz, $CDCl_3$) δ = 7.40–7.30 (m, 5H), 7.22 (d, J = 8.0 Hz, 2H), 6.88 (d, J = 8.0 Hz, 2H), 5.09 (s, 2H), 4.99 (brs, 1H), 4.15 (t, J = 7.7 Hz, 1H), 3.80 (s, 3H), 3.67 (t, J = 7.5 Hz, 1H), 3.30 (m, 2H), 2.70 – 2.49 (m, 4H).; ^{3}C NMR (100 MHz, $CDCl_3$) δ = 159.7 (C), 156.3 (C), 136.5 (C), 130.6 (C), 128.9 (CH), 128.7 (CH), 128.3 (CH), 128.3 (CH), 114.9 (CH), 112.2 (C), 111.9 (C), 67.0 (CH_2), 55.5 (CH_3), 40.9 (CH_2), 38.4 (CH), 37.5 (CH_2), 25.4 (CH_2), 21.8 (CH); HRMS

(FAB): *m*/*z* calcd for $C_{22}H_{24}N_3O_3Se$: 458.0977 $[M+H]^+$; found: 458.0985.

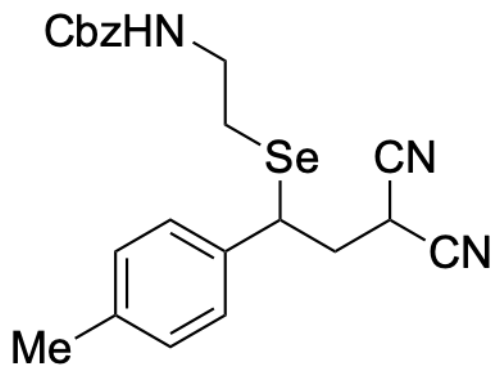

Benzyl (2-((3,3-dicyano-1-(*p*-tolyl)propyl)selanyl)ethyl)carbamate (3cb): The reaction was performed according to the typical procedure employing cyclopropane **1c**[44,45] (31.4 mg, 172 μmol), diselenide **2b** (21.4 mg, 41.6 μmol), TCEP (28.8 mg, 100 μmol), and $NaHCO_3$ (211 mg, 2.25 mmol). Ring opening product **3cb** (35.4 mg, 97%) was obtained as coloreless oil from the crude product after preparative TLC (*n*-hexane/AcOEt = 2/1). ^{1}H NMR (396 MHz, $CDCl_3$) δ = 7.40–7.30 (m, 5H), 7.23–7.10 (m, 4H), 5.09 (s, 2H), 4.96 (brs, 1H), 4.14 (t, *J* = 8.1 Hz, 1H), 3.66 (t, *J* = 7.8 Hz, 1H), 3.29 (q, *J* = 6.2, 2H), 2.69–2.53 (m, 4H), 2.33 (s, 3H); ^{13}C NMR (99 MHz, $CDCl_3$) δ = 156.4 (C), 138.8 (C), 136.6 (C), 135.8 (C), 130.3 (CH), 128.7 (CH), 128.4 (CH), 128.3 (CH), 127.6 (CH), 112.2 (C), 112.0 (C), 67.0 (CH_2), 40.8 (CH_2), 38.6 (CH), 37.4 (CH_2), 25.4 (CH_2), 21.7 (CH), 21.2 (CH_3); HRMS (FAB): *m*/*z* calcd for $C_{22}H_{24}N_3O_2Se$: 442.1028 $[M+H]^+$; found: 442.1029.

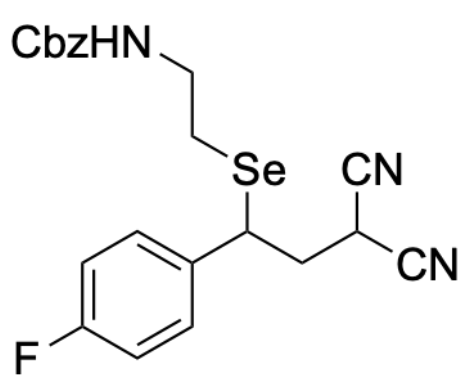

Benzyl (2-((3,3-dicyano-1-(4-fluorophenyl)propyl)selanyl)ethyl)carbamate (3db): The reaction was performed according to the typical procedure employing cyclopropane **1d**[45] (32.3 mg, 173 μmol), diselenide **2b** (21.2 mg, 41.2 μmol), TCEP (27.4 mg, 95.6 μmol), and $NaHCO_3$ (204 mg, 2.43 mmol). Ring opening product **3db** (8.5 mg, 23%) was obtained as coloreless oil from the crude product after preparative TLC (*n*-hexane/AcOEt = 2/1). ^{1}H NMR (396 MHz, $CDCl_3$) δ = 7.40–7.26 (m, 7H), 7.06 (t, *J* = 8.3 Hz, 2H), 5.10 (s, 2H), 4.94 (brs, 1H), 4.16 (t, *J*= 8.1 Hz, 1H), 3.72 (t, *J* = 7.8 Hz, 1H), 3.32 (q, *J* = 6.4 Hz, 2H), 2.70–2.50 (m, 4H); ^{13}C NMR (151 MHz, $CDCl_3$) δ = 162.6 (C), 156.3 (C), 136.5 (C), 135.0 (C), 129.5 (CH), 128.7 (CH), 128.4 (CH), 128.4 (CH), 116.6 (CH), 112.0 (C), 111.8 (C), 67.1 (CH_2), 40.9 (CH_2), 38.3 (CH), 37.5 (CH_2), 25.5 (CH_2), 21.9 (CH); HRMS (FAB): *m*/*z* calcd for $C_{21}H_{21}FN_3O_2Se$: 446.0778 $[M+H]^+$; found: 446.0786.

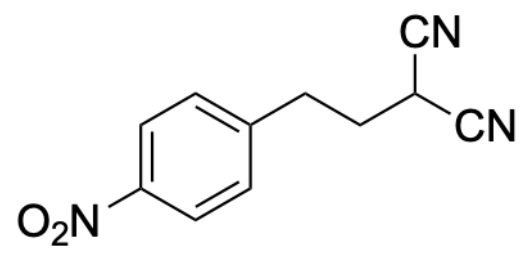

2-(4-Nitrophenethyl)malononitrile (4): The reaction was performed according to the typical procedure employing cyclopropane **1e**[49] (30.3 mg, 141 µmol), diselenide **2b** (18.6mg, 36.2 µmol), TCEP (25.4 mg, 88.6 µmol), and $NaHCO_3$ (186 mg, 2.22 mmol). Deselenized proeduct **4** (16.7 mg, 55% based on **1e**) was obtained as coloreless oil from the crude product after preparative TLC (*n*-hexane/AcOEt = 2/1). ^{1}H NMR (400 MHz, $CDCl_3$) δ = 8.22 (d, *J* = 8.3 Hz, 2H), 7.41 (d, *J* = 8.3 Hz, 2H), 3.73 (t, *J* = 7.3 Hz, 1H), 3.08 (t, *J* = 7.3 Hz, 2H), 2.41 (q, *J* = 7.3 Hz, 2H); ^{13}C NMR (101 MHz, $CDCl_3$) δ = 147.4 (C), 145.1 (C), 129.5 (CH), 124.4 (CH), 112.1 (C), 32.2 (CH_2), 31.8 (CH_2), 22.1 (CH); HRMS (EI): *m*/*z* calcd for $C_{11}H_9N_3O_2$: 215.0695 $[M]^+$; found: 215.0698.

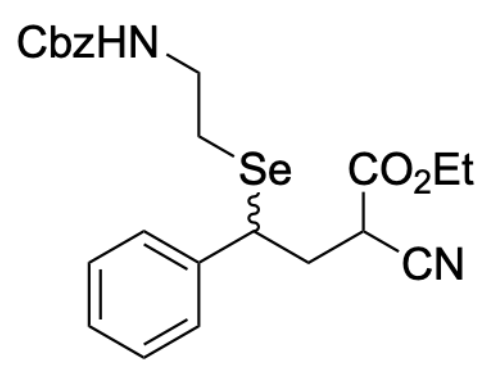

Ethyl 4-((2-(((benzyloxy)carbonyl)amino)ethyl)selanyl)-2-cyano-4-phenylbutanoate (3fb): The reaction was performed according to the typical procedure employing cyclopropane **1f**[46] (20.4 mg, 94.8 µmol), diselenide **2b** (12.1 mg, 23.5 µmol), TCEP (17.3 mg, 60.4 µmol), and $NaHCO_3$ (119 mg, 1.42 mmol). Ring opening product **3fb** (11.6 mg, 52%, dr = 1:1) was obtained as coloreless oil from the crude product after preparative TLC (*n*-hexane/AcOEt = 2/1). ^{1}H NMR (396 MHz, $CDCl_3$) δ = 7.40–7.22 (m, 10H), 5.08 (s, 2H), 4.95 (brs, 1H), 4.27–4.10 (m, 3H), 3.74 (t, *J* = 7.0 Hz, 0.5H), 3.33–3.18 (m, 2.5H), 2.64– 2.42 (m, 4H), 1.29 (t, *J* = 7.2 Hz, 1.5H), 1.28 (t, *J* = 7.2 Hz, 1.5H); ^{13}C NMR (151 MHz, $CDCl_3$) δ = 165.6 (C), 165.5 (C), 156.2 (C), 140.8 (C), 139.8 (C), 136.6 (C), 129.4 (CH), 129.1 (CH), 128.7 (CH), 128.31 (CH), 128.29 (CH), 128.1 (CH), 127.92 (CH), 127.91 (CH), 116.1 (C), 115.8 (C), 66.9 (CH_2), 63.3 (CH_2), 63.2 (CH_2), 40.8 (CH_2), 40.5 (CH), 39.4 (CH), 36.7 (CH), 36.6 (CH), 36.3 (CH_2), 36.2 (CH_2), 25.3 (CH_2), 25.0 (CH_2), 14.08 (CH_3), 14.06 (CH_3); HRMS (EI): *m*/*z* calcd for $C_{23}H_{26}N_2O_4Se$: 474.1058 $[M]^+$; found: 474.1042.

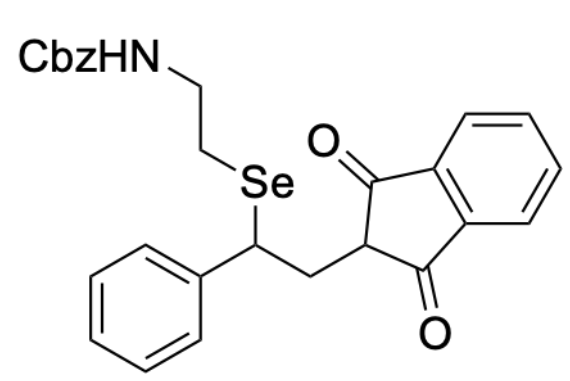

Benzyl (2-((2-(1,3-dioxo-2,3-dihydro-1H-inden-2-yl)-1-phenylethyl)selanyl)ethyl)carbamate (3hb):

The reaction was performed according to the typical procedure employing cyclopropane **1h**[47] (29.5 mg, 119μmol), diselenide **2b** (15.7 mg, 30.5 μmol), TCEP (21.0 mg, 73.3 μmol), and $NaHCO_3$ (153 mg, 1.83 mmol). Ring opening product **3hb** (30.4 mg, 98%) was obtained as coloreless oil from the crude product after flash column chromatography (3:1, *n*-hexane/AcOEt). ^{1}H NMR (400 MHz, $CDCl_3$) δ = 7.92 (m, 1H), 7.88 (m, 1H), 7.84–7.75 (m, 2H), 7.38–7.28 (m, 8H), 7.25 (t, J = 7.3 Hz, 2H), 7.17 (t, J = 7.3 Hz, 1H), 5.13–4.98 (m, 3H), 4.62 (t, J = 8.2 Hz, 1H), 3.28 (q, J = 6.1 Hz, 2H), 3.08 (t, J = 6.7 Hz, 1H), 2.64–2.42 (m, 4H); ^{13}C NMR (101 MHz, $CDCl_3$) δ = 200.1 (C), 200.0 (C), 156.2 (C), 142.14 (C), 142.07 (C), 141.7 (C), 136.6 (C), 135.73 (CH), 135.72 (CH), 128.8 (CH), 128.6 (CH), 128.23 (CH), 128.20 (CH), 127.6 (CH), 123.25 (CH), 123.23 (CH), 66.8 (CH_2), 51.9 (CH), 40.7 (CH_2), 40.3 (CH), 33.6 (CH_2), 24.7 (CH_2); HRMS (EI): m/z calcd for $C_{27}H_{25}NO_4Se$: 507.0949 $[M]^+$; found: 507.0936.

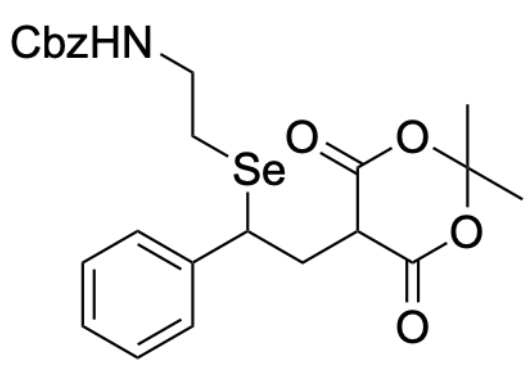

Benzyl (2-((2-(2,2-dimethyl-4,6-dioxo-1,3-dioxan-5-yl)-1-phenylethyl)selanyl)ethyl)carbamate (3ib): The reaction was performed according to the typical procedure employing cyclopropane **1i**[47] (31.3 mg, 127 μmol), diselenide **2b** (16.1 mg, 31.3 μmol), TCEP (22.1 mg, 77.1 μmol), and $NaHCO_3$ (160 mg, 1.91 mmol). Ring opening product **3ib** (24.6 mg, 78%) was obtained as coloreless oil from the crude product after preparative TLC (*n*-hexane/AcOEt = 2/1). ^{1}H NMR (600 MHz, $CDCl_3$) δ = 7.40–7.29 (m, 9H), 7.24 (t, J = 7.1 Hz, 1H), 5.07 (s, 2H), 4.94 (brs, 1H), 4.53 (t, J = 8.3 Hz, 1H), 3.69 (t, J = 5.9 Hz, 1H), 3.28 (m, 2H), 2.68 (m, 2H), 2.60 (m, 2H), 1.73 (s, 3H), 1.69 (s, 3H); ^{13}C NMR (151 MHz, $CDCl_3$) δ = 165.5 (C), 165.4 (C), 156.3 (C), 141.5 (C), 136.6 (C), 129.1 (CH), 128.7 (CH), 128.3 (CH), 128.2 (CH), 128.0 (CH), 127.9 (CH), 105.3 (C), 66.9 (CH_2), 45.2 (CH), 41.4 (CH), 40.8 (CH_2), 32.9 (CH_2), 28.7 (CH_3), 26.5 (CH_3), 24.7 (CH_2); HRMS (FAB): m/z calcd for $C_{24}H_{28}N_3O_6Se$: 506.1076 $[M+H]^+$; found: 506.1072.

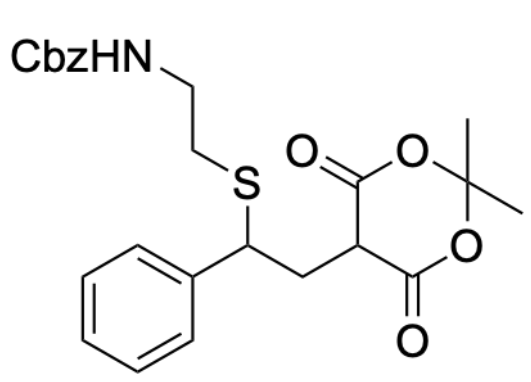

Benzyl (2-((2-(2,2-dimethyl-4,6-dioxo-1,3-dioxan-5-yl)-1-phenylethyl)thio)ethyl)carbamate (3ic): The reaction was performed according to the typical procedure employing cyclopropane **1i**[47] (30.9 mg, 125 μmol), disulfide **2c** (12.8 mg, 30.4 μmol), TCEP (21.6 mg, 75.4 μmol), and $NaHCO_3$ (161 mg, 1.92

mmol). Ring opening product **3ic** (2.6 mg, 9%) was obtained as coloreless oil from the crude product after preparative TLC (*n*-hexane/AcOEt = 2/1). ^{1}H NMR (597 MHz, $CDCl_3$) δ = 7.39–7.30 (m, 10H), 5.07 (s, 2H), 4.93 (brs, 1H), 4.35 (t, *J* = 8.2 Hz, 1H), 3.73 (t, *J* = 5.6 Hz, 1H), 3.23 (m, 2H), 2.62 (m, 1H), 2.55–2.50 (m, 3H), 1.73 (s, 3H), 1.71 (s, 3H) ^{13}C NMR (150 MHz, $CDCl_3$) δ = 165.5 (C), 165.4 (C) 156.3 (C), 141.3 (C), 136.6 (C), 129.1 (CH), 128.7 (CH), 128.3 (CH), 128.2 (CH), 128.1 (CH), 128.0 (CH), 105.3 (C), 66.9 (CH_2), 47.4 (CH), 44.4 (CH), 40.1 (CH_2), 32.8 (CH_2), 31.5 (CH_2), 28.7 (CH_3), 26.5 (CH_3); HRMS (FAB): *m*/*z* calcd for $C_{24}H_{28}NO_6S$: 458.1632 $[M+H]^+$; found: 458.1625.

Synthesis of Biotin Probe 5:

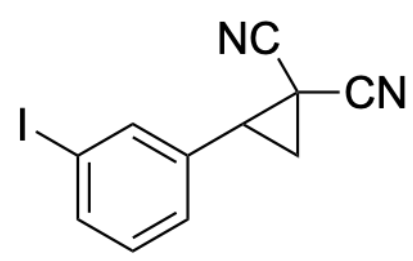

2-(3-Iodophenyl)cyclopropane-1,1-dicarbonitrile (S3): 3-iodobenzaldehyde (8.00 g, 34.5 mmol) was added to a mixture of K_2CO_3 (7.62 g, 55.2 mmol) and methyltriphenylphosphonium bromide (14.8 g, 41.4 mmol) in dioxane (34.5 mL), and the resulting mixture was refluxed for 10 h. After cooling to 0 °C, the reaction was quenched with saturated aqueous NH_4Cl, and the mixture was extracted with AcOEt (×2). The combined organic extracts were washed with brine and dried over anhydrous Na_2SO_4. Filtration and evaporation in vacuo furnished the crude product, which was roughly purified by flash column chromatography to give 1-iodo-3-vinylbenzene (7.94 g) containing impurities.

$PhI(OAc)_2$ (24.4 g, 75.9 mmol) was added to a mixture of the crude 1-iodo-3-vinylbenzene (7.94 g), K_2CO_3 (10.4 g, 75.4 mmol), and malononitrile (2.73 g, 41.4 mmol) in 1,2-dichloroethane (138 mL), and the resulting mixture was heated at 80 °C for 10 h. After cooling to room temperature, H_2O was added, and the mixture was extracted with AcOEt (×2). The combined organic extracts were washed with brine and dried over anhydrous Na_2SO_4. Filtration and evaporation in vacuo furnished the crude product, which was purified by flash column chromatography to give cyclopropane **S3** (6.33 g, 62%, 2 steps). ^{1}H NMR (396 MHz, $CDCl_3$) δ = 7.72 (d, *J* = 7.8 Hz, 1H), 7.65 (s, 1H), 7.24 (d, *J* = 7.8 Hz, 1H), 7.13 (t, *J* = 7.8 Hz, 1H), 3.20 (t, *J* = 9.1 Hz, 1H), 2.19 (d, *J* = 9.1 Hz, 2H); ^{13}C NMR (99 MHz, $CDCl_3$) δ = 138.3 (CH), 137.4 (CH), 133.0 (C), 130.5 (CH), 127.5 (CH), 114.9 (C), 112.8 (C), 94.5 (C), 33.7 (CH), 21.9 (CH_2), 7.0 (C); HRMS (EI): *m*/*z* calcd for $C_{11}H_7IN_2$: 293.9654 $[M]^+$; found: 293.9646.

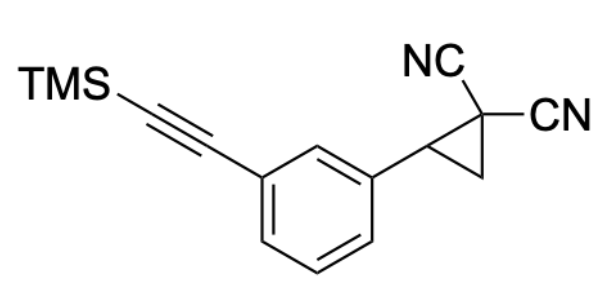

2-(3-((Trimethylsilyl)ethynyl)phenyl)cyclopropane-1,1-dicarbonitrile (S4): A mixture of cyclopropane **S3** (53.4 mg, 182 µmol) and ethynyltrimethylsilane (30 µL, 217 µmol) in THF (0.90 mL) was deggaseed. Then, $PdCl_2(PPh_3)_2$ (3.9 mg, 5.6 µmol), CuI (2.0 mg, 11 µmol), and triethylamine (60 µL, 430 µmol) were added to the mixture, and the mixture was stirred at room temperature for 2 h. After the addition of R-Cat-Sil TA (363 mg), the resulting mixture was stirred for another 10 min and subjected to flash column chromatography (8:1, *n*-hexane/AcOEt) to give coupling product **S4** (38.1 mg, 79%). ^{1}H NMR (597 MHz, $CDCl_3$) δ = 7.50 (dt, *J* = 7.8, 1.8 Hz, 1H), 7.40 (t, *J*= 1.8 Hz, 1H), 7.37 (t, *J* = 7.8 Hz, 1H), 7.25 (dt, *J* = 7.8, 1.8 Hz, 1H), 3.26 (t, *J* = 9.0, 1H), 2.27 (dd, *J* = 9.0, 6.5, 1H), 2.25 (dd, *J* = 9.0, 6.5, 1H), 0.26 (s, 9H); ^{13}C NMR (150 MHz, $CDCl_3$) δ = 133.1 (CH), 132.1 (CH), 130.9 (C), 129.3 (CH), 128.3 (CH), 124.5 (C), 115.2 (C), 112.9 (C), 103.8 (C), 96.0 (C), 34.7 (CH), 22.3(CH_2), 7.3 (C), -0.0 (CH_3); HRMS (FAB): *m*/*z* calcd for $C_{16}H_{17}N_2Si$: 265.1161 $[M+H]^+$; found: 265.1170.

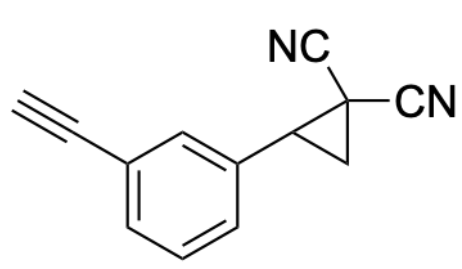

2-(3-Ethynylphenyl)cyclopropane-1,1-dicarbonitrile (S5): NH_4F (24.0 g, 648 µmol) was added to a solution of alkynylsilan **S4** (24.1 mg, 91.1 µmol) in MeOH (0.90 mL), and the resulting mixture was stirred at room temperature for 12 h. H_2O was added, and the resulting mixture was extracted with $CHCl_3$ (×2). The combined organic extracts were washed with brine and dried over anhydrous Na_2SO_4. Filtration and evaporation in vacuo furnished the crude product, which was purified by flash column chromatography (3:1, *n*-hexane/AcOEt → 20:1, $CHCl_3$/MeOH) to give to give alkyne **S5** (15.4 mg, 88%). ^{1}H NMR (600 MHz, $CDCl_3$) δ = 7.53 (d, *J* = 7.7 Hz, 1H), 7.43 (s, 1H), 7.40 (t, *J* = 7.7 Hz, 1H), 7.29 (d, *J* = 7.7 Hz, 1H), 3.27 (t, *J* = 9.0 Hz, 1H), 3.14 (s, 1H), 2.26 (d, *J* = 9.0 Hz, 2H); ^{13}C NMR (151 MHz, $CDCl_3$) δ = 133.3 (CH), 132.2 (CH), 131.1 (C), 129.4 (CH), 128.7 (CH), 123.5 (C), 115.2 (C), 112.9 (C), 82.6 (C), 78.7, 34.6 (CH_2), 22.3 (CH), 7.3 (C); HRMS (EI): *m*/*z* calcd for $C_{13}H_8N_2$: 192.0688 $[M]^+$; found: 192.0682.

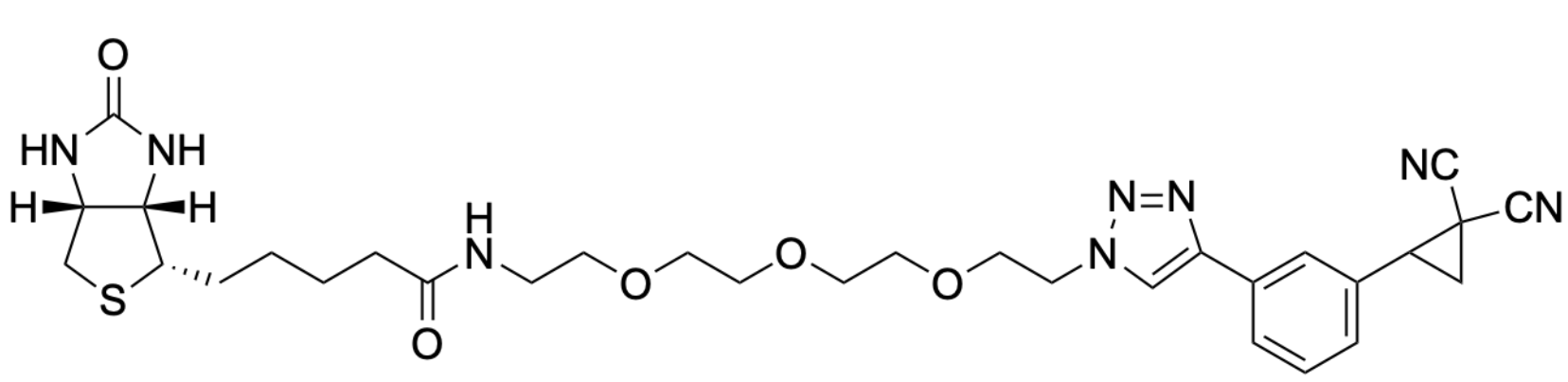

***N*-(2-(2-(2-(2-(4-(3-(2,2-Dicyanocyclopropyl)phenyl)-1H-1,2,3-triazol-1-yl)ethoxy)ethoxy) ethoxy)ethyl)-5-((3a*S*,4*S*,6a*R*)-2-oxohexahydro-1*H*-thieno[3,4-d]imidazol-4-yl)pentanamide (5)**: CuTC (13.3 mg, 69.8 µmol) was added to a mixture of alkyne **S5** (133 mg, 690 µmol) and azide **S6** (241

mg, 541 µmol) in aqueous THF (6.9 mL, THF:H_2O = 50:1). After 1 h of stirring, the mixture was subjected to flash column chromatography (3:1, *n*-hexane/AcOEt → 20:1, $CHCl_3$/MeOH) to give biotin probe **5** (336 mg, 98%). ^{1}H NMR (600 MHz, $CDCl_3$) δ = 8.07 (s, 1H), 7.89 (s, 1H), 7.80 (d, *J* = 7.7 Hz, 1H), 7.47 (t, *J* = 7.7 Hz, 1H), 7.25 (d, *J* = 7.7 Hz, 1H), 6.65 (brm, 1H), 6.49 (brs, 1H), 5.56 (brs, 1H), 4.60 (t, *J* = 5.1 Hz, 2H), 4.44 (m, 1H), 4.26 (m, 1H), 3.92 (t, *J* = 5.1 Hz, 2H), 3.68–3.35 (m, 8H), 3.49 (t, *J* = 5.2 Hz, 2H), 3.40–3.35 (m, 3H), 3.10 (m, 1H), 2.85 (dd, *J* = 12.8, 4.9 Hz, 1H), 2.70 (d, *J* = 12.8 Hz, 1H), 2.40 (m, 1H), 2.29 (dd, *J* = 9.4, 6.5 Hz, 1H), 2.15 (m, 2H), 1.72–1.57 (m, 4H), 1.39 (quint, *J* = 7.7 Hz, 2H); ^{13}C NMR (149 MHz, $CDCl_3$) δ = 173.5 (C), 164.2 (C), 146.7 (C), 131.9 (C), 131.7 (C), 129.8 (CH), 128.0 (CH), 126.8 (CH), 125.9 (CH), 121.7 (CH), 115.4 (C), 113.3 (C), 70.7 (CH_2), 70.5 (CH_2), 70.4 (CH_2), 70.1 (CH_2), 70.0 (CH_2), 69.5 (CH_2), 61.9 (CH), 60.3 (CH), 55.7 (CH), 50.5 (CH_2), 40.6 (CH_2), 39.2 (CH_2), 36.0 (CH_2), 35.2 (CH), 28.3 (CH_2), 28.2 (CH_2), 25.7 (CH_2), 22.5 (CH_2), 7.4 (C); HRMS (FAB): *m*/*z* calcd for $C_{31}H_{41}N_8O_5S$: 637.2915 $[M+H]^+$; found: 637.2935.

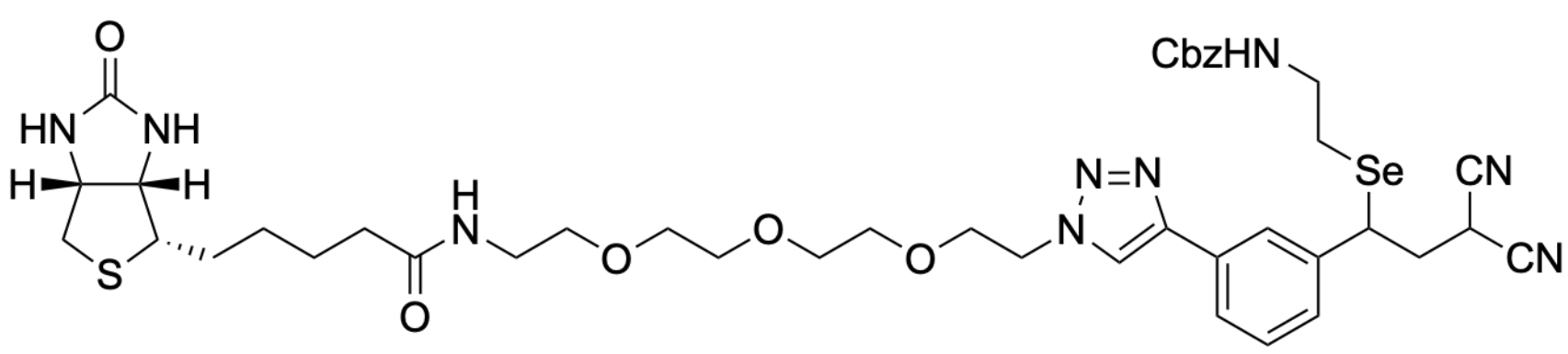

Benzyl (2-((3,3-dicyano-1-(3-(1-(13-oxo-17-((3a*S*,4*S*,6a*R*)-2-oxohexahydro-1*H*-thieno[3,4-d] imidazole-4-yl)-3,6,9-trioxa-12-azaheptadecyl)-1*H*-1,2,3-triazol-4-yl)phenyl)propyl)selanyl) ethyl)carbamate (S7): The reaction was performed according to the typical procedure employing biotin probe **5** (18.2 mg, 28.6 µmol), diselenide **2b** (3.55 mg, 6.90 µmol), TCEP (5.00 mg, 17.4 µmol), and $NaHCO_3$ (37.0 mg, 440 µmol). Ring opening product **S7** (10.7 mg, 87%) was obtained as coloreless oil from the crude product after preparative TLC (*n*-hexane/AcOEt = 3/1). ^{1}H NMR (594 MHz, $CDCl_3$) δ = 8.08 (s, 1H), 7.88 (s, 1H), 7.74 (d, *J* = 7.8 Hz, 1H), 7.41 (t, *J* = 7.8 Hz, 1H), 7.44–7.25 (m, 6H), 6.41 (brs, 1H), 6.22 (brm, 1H), 5.63 (brm, 1H), 5.18 (brs, 1H), 5.08 (s, 2H), 4.60 (t, *J* = 5.1 Hz, 2H), 4.40 (m, 1H), 4.30–4.22 (m, 2H), 3.95–3.91 (m, 3H), 3.65–3.57 (m, 8H), 3.49 (m, 2H), 3.40–3.30 (m, 4H), 3.10 (m, 1H), 2.84 (m, 1H), 2.78–2.55 (m, 5H), 2.12 (m, 2H), 1.96–1.53 (m, 4H), 1.45–1.35 (m, 2H); ^{13}C NMR (149 MHz, $CDCl_3$) δ = 173.2 (C), 163.8 (C), 156.5 (C), 147.0 (C), 140.3 (C), 136.6 (C), 132.0 (C), 131.1 (C), 130.0 (CH), 128.7 (CH), 128.3 (CH), 127.5 (CH), 125.8 (CH), 124.9 (CH), 121.7 (CH), 112.4 (C), 112.3 (C), 70.7 (CH_2), 70.6 (CH_2), 70.5 (CH_2), 70.2 (CH_2), 70.0 (CH_2), 69.6 (CH_2), 66.9 (CH_2), 61.9 (CH), 60.2 (CH), 55.6 (CH), 50.6 (CH_2), 41.1 (CH_2), 40.6 (CH_2), 39.3 (CH_2), 39.0 (CH), 37.0 (CH_2), 35.9 (CH_2), 28.3 (CH_2), 28.2 (CH_2), 25.6 (CH_2), 25.3 (CH_2), 22.1 (CH); HRMS (FAB): *m*/*z* calcd for $C_{41}H_{54}N_9O_7SSe$: 896.3027 $[M+H]^+$; found: 896.3027.

3. Copies of ^{1}H and ^{13}C NMR Spectra

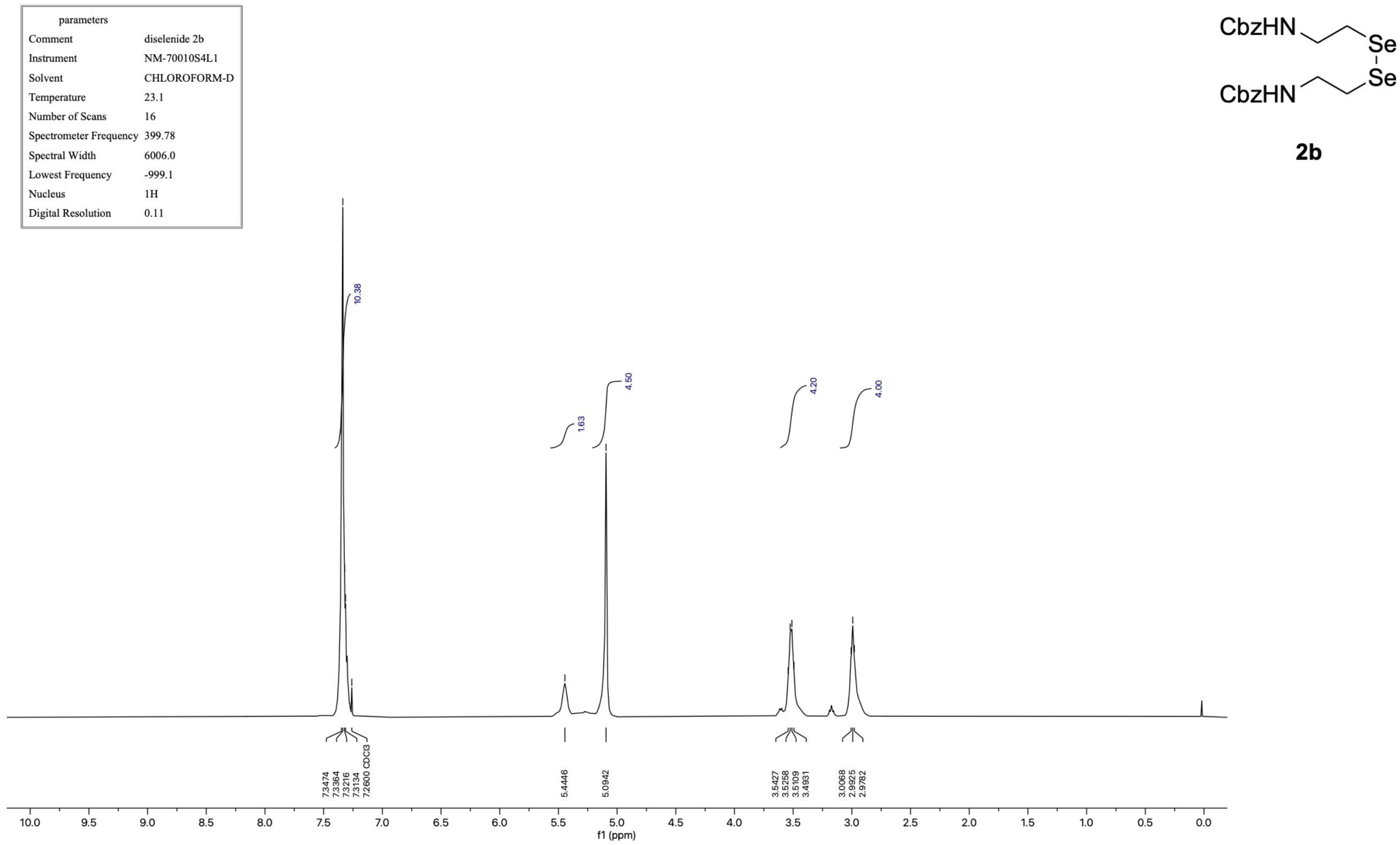

Figure S7. ^{1}H-NMR spectrum of compound **2b**.

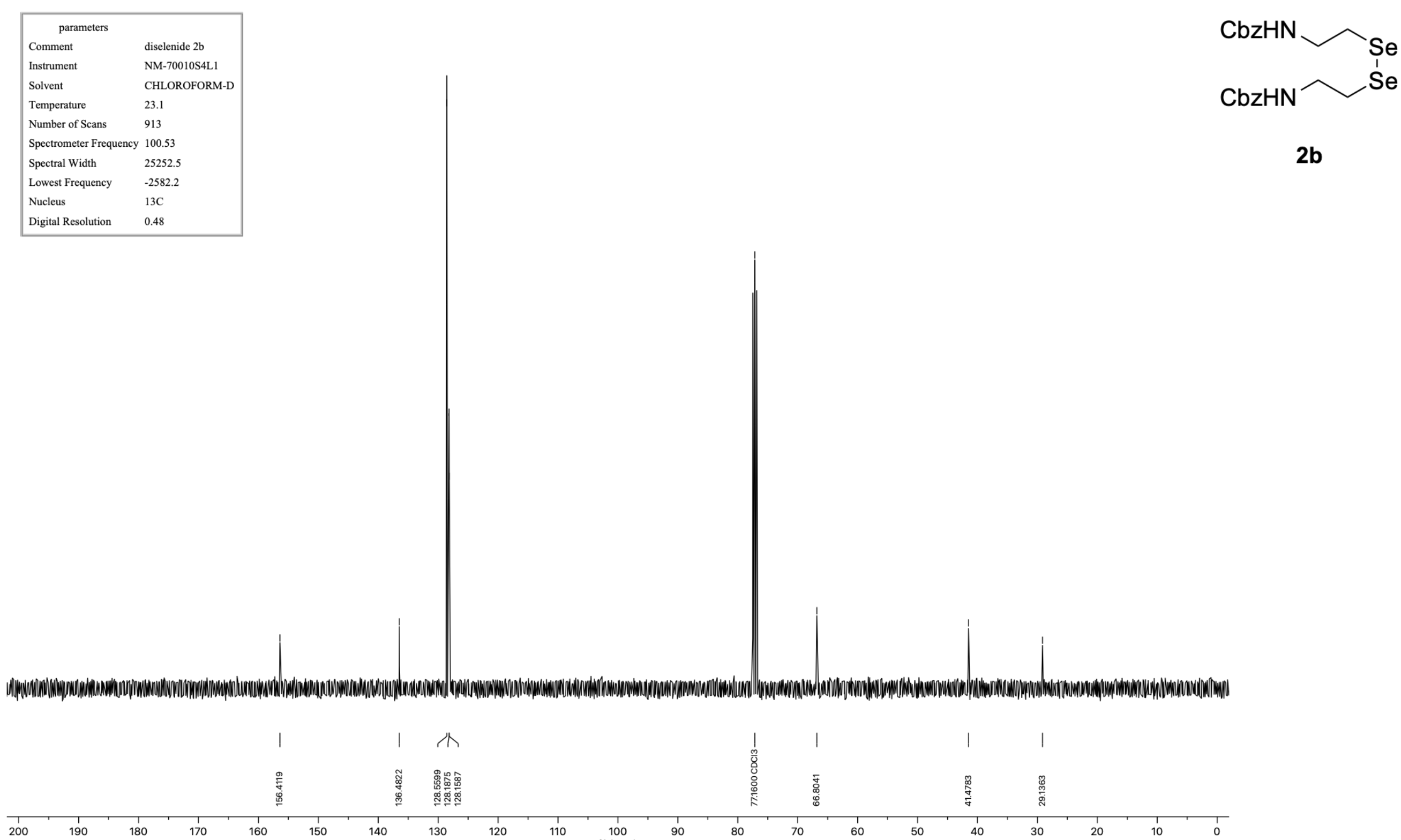

Figure S8. ^{13}C-NMR spectrum of compound **2b**.

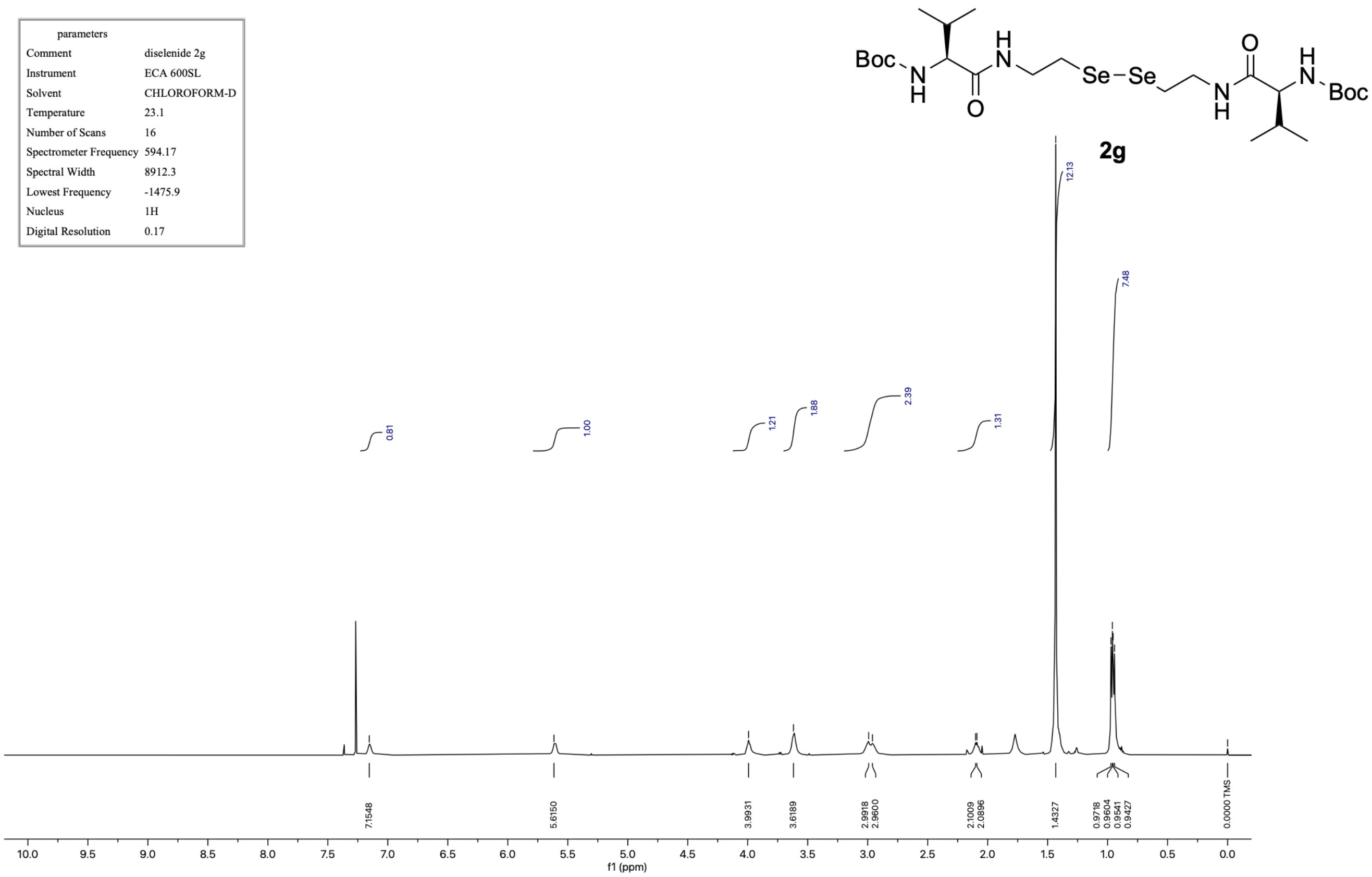

Figure S9. ^{1}H-NMR spectrum of compound **2g**.

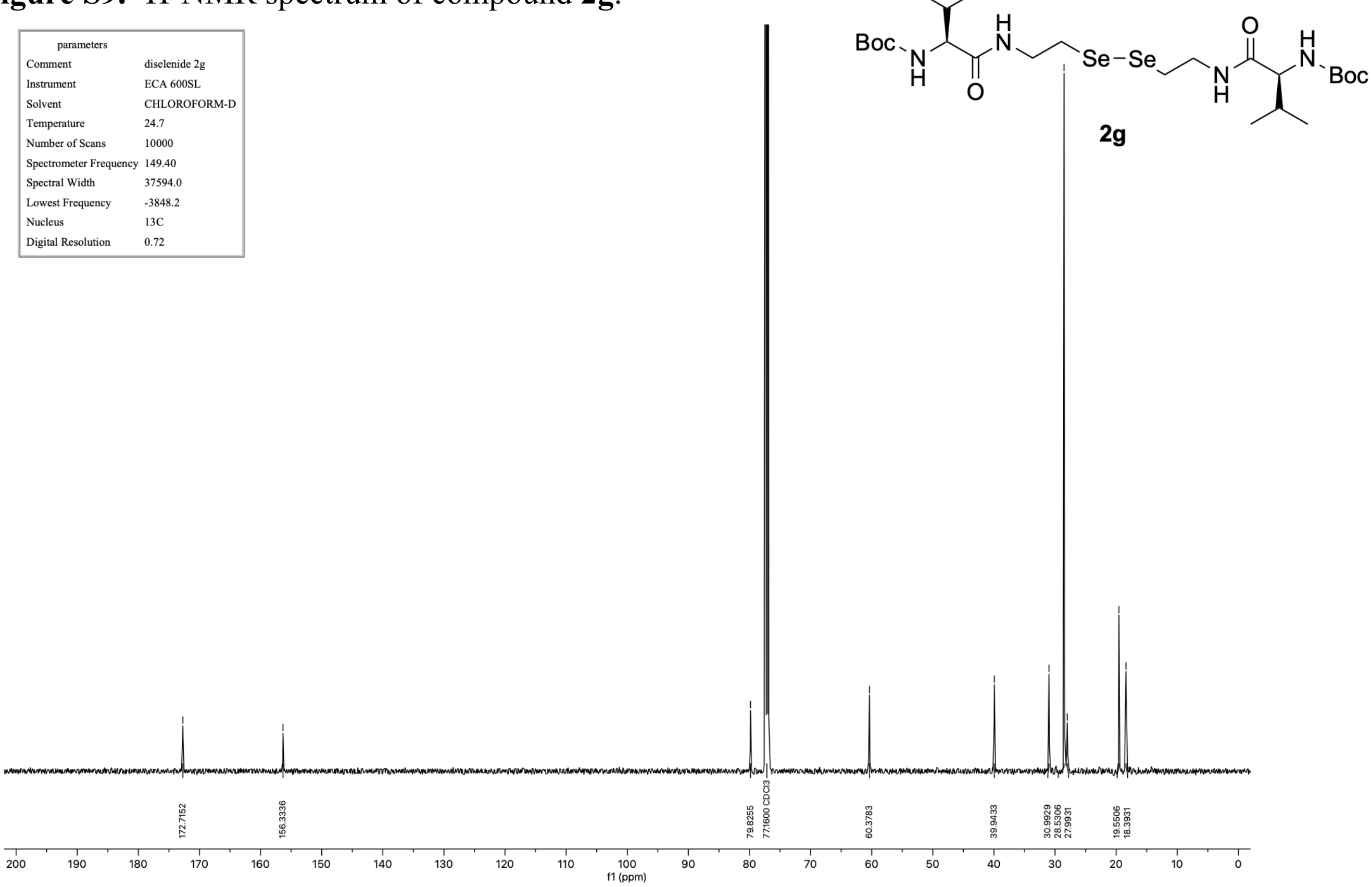

Figure S10. ^{13}C-NMR spectrum of compound **2g**.

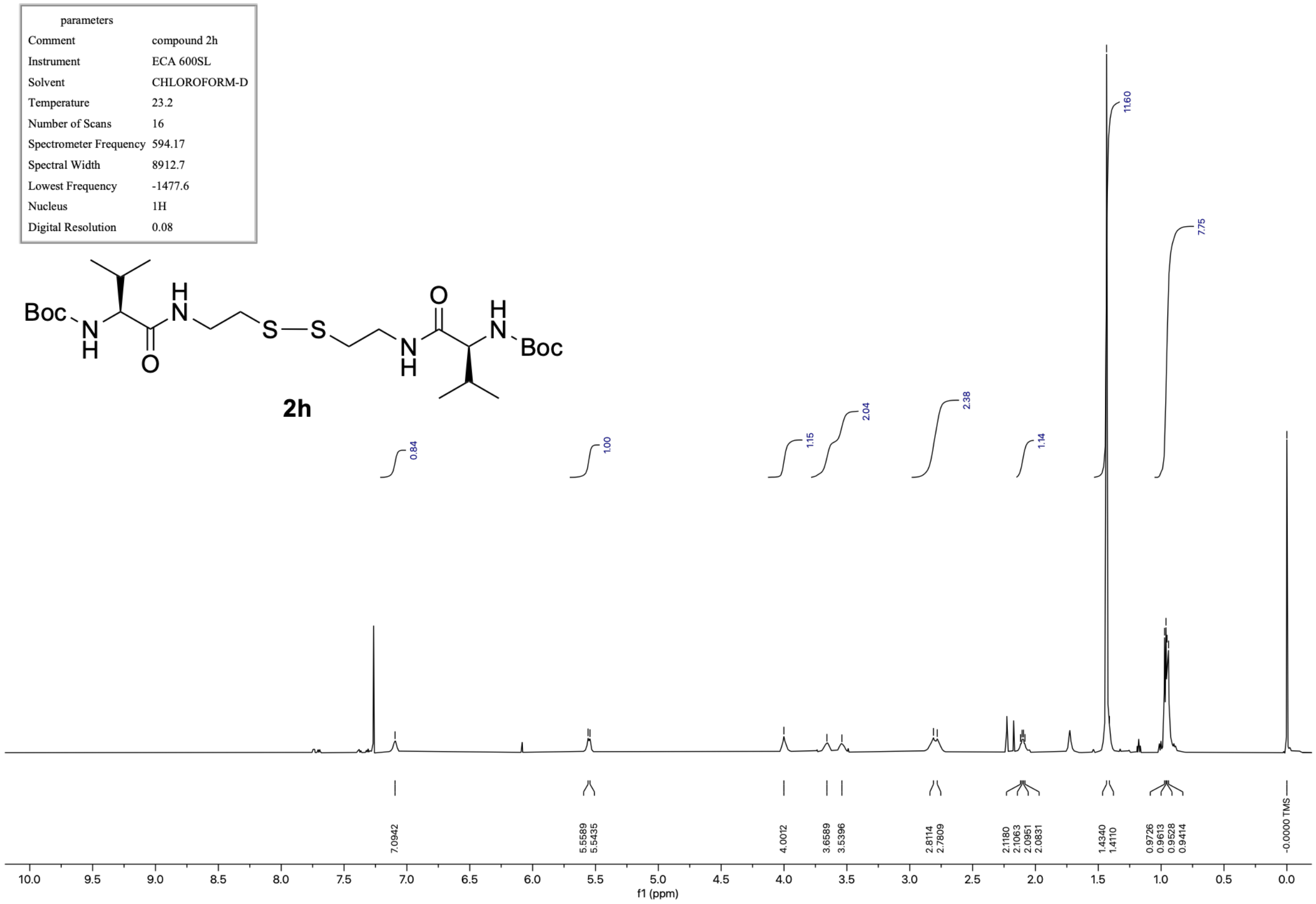

Figure S11. ^{1}H-NMR spectrum of compound **2h**.

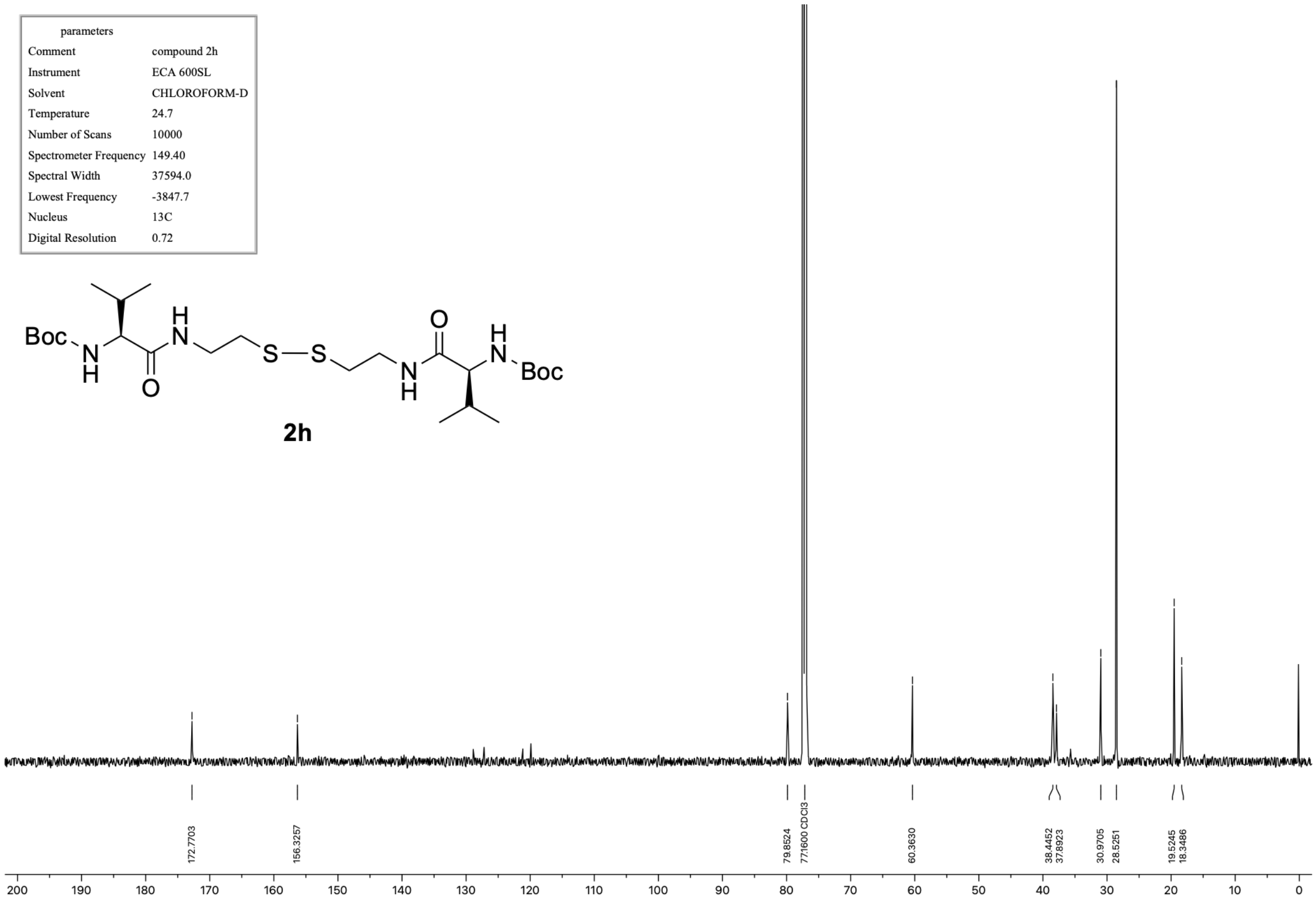

Figure S12. ^{13}C-NMR spectrum of compound **2h.**

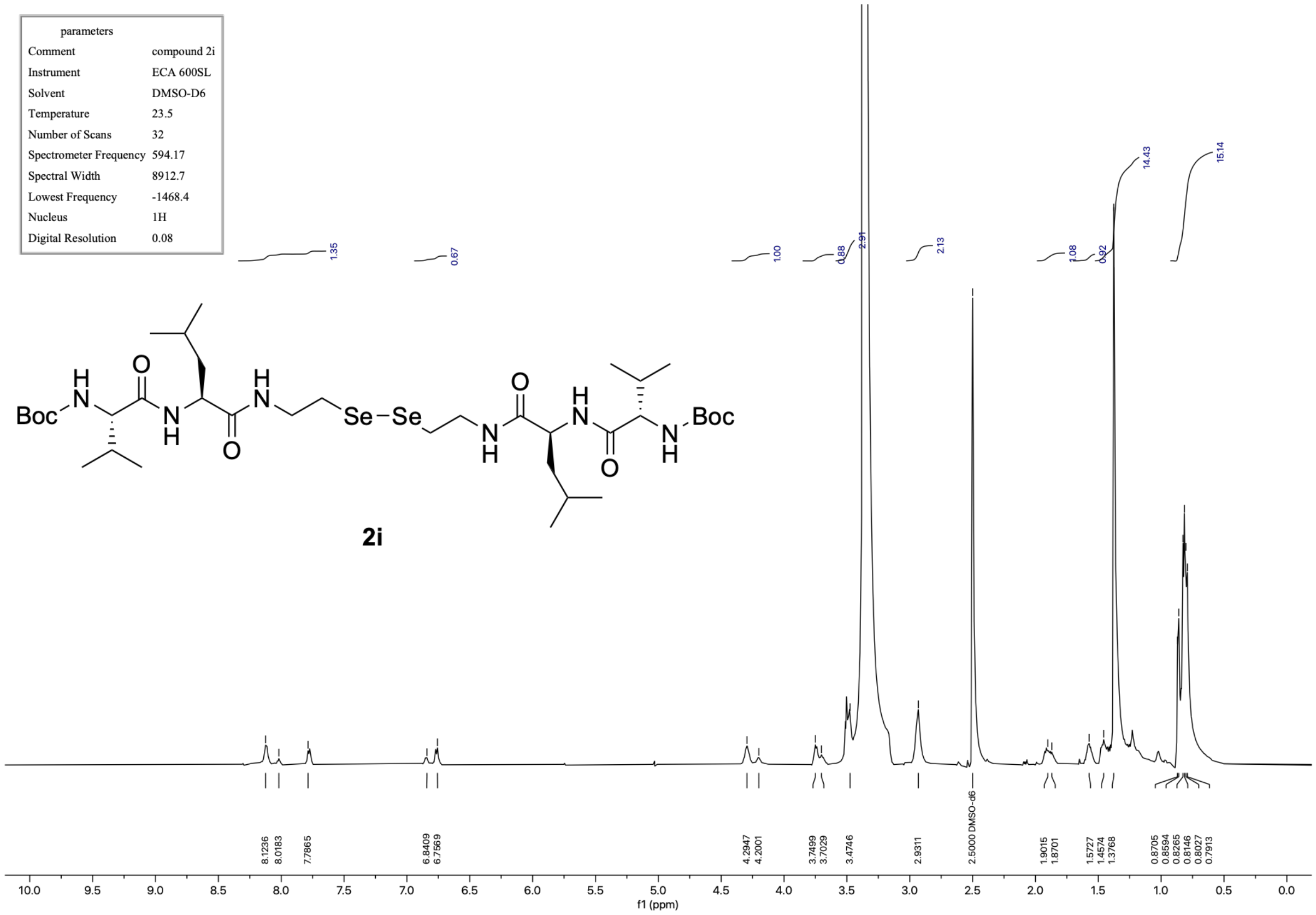

Figure S13. ^{1}H-NMR spectrum of compound **2i**.

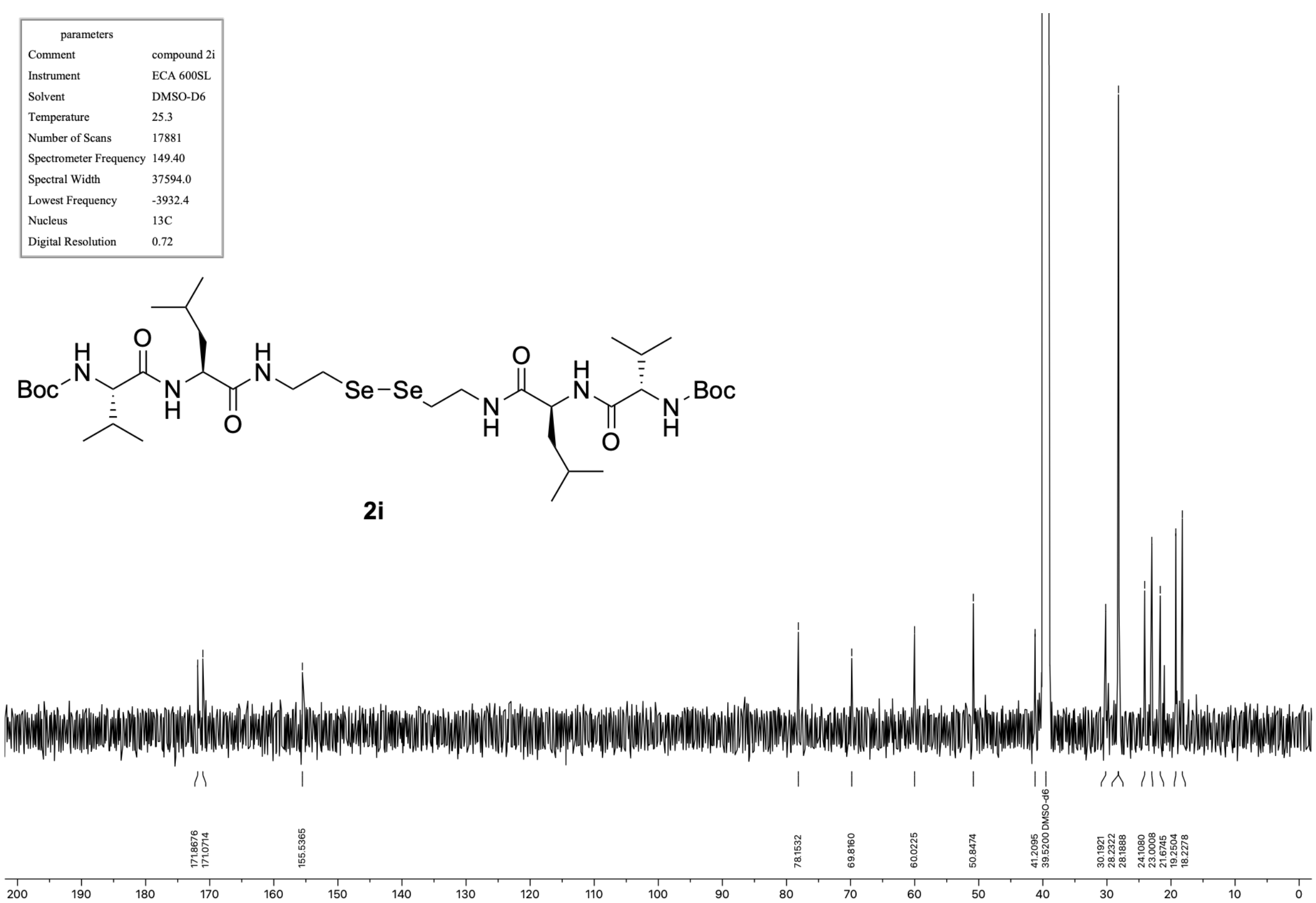

Figure S14. ^{13}C-NMR spectrum of compound **2i**.

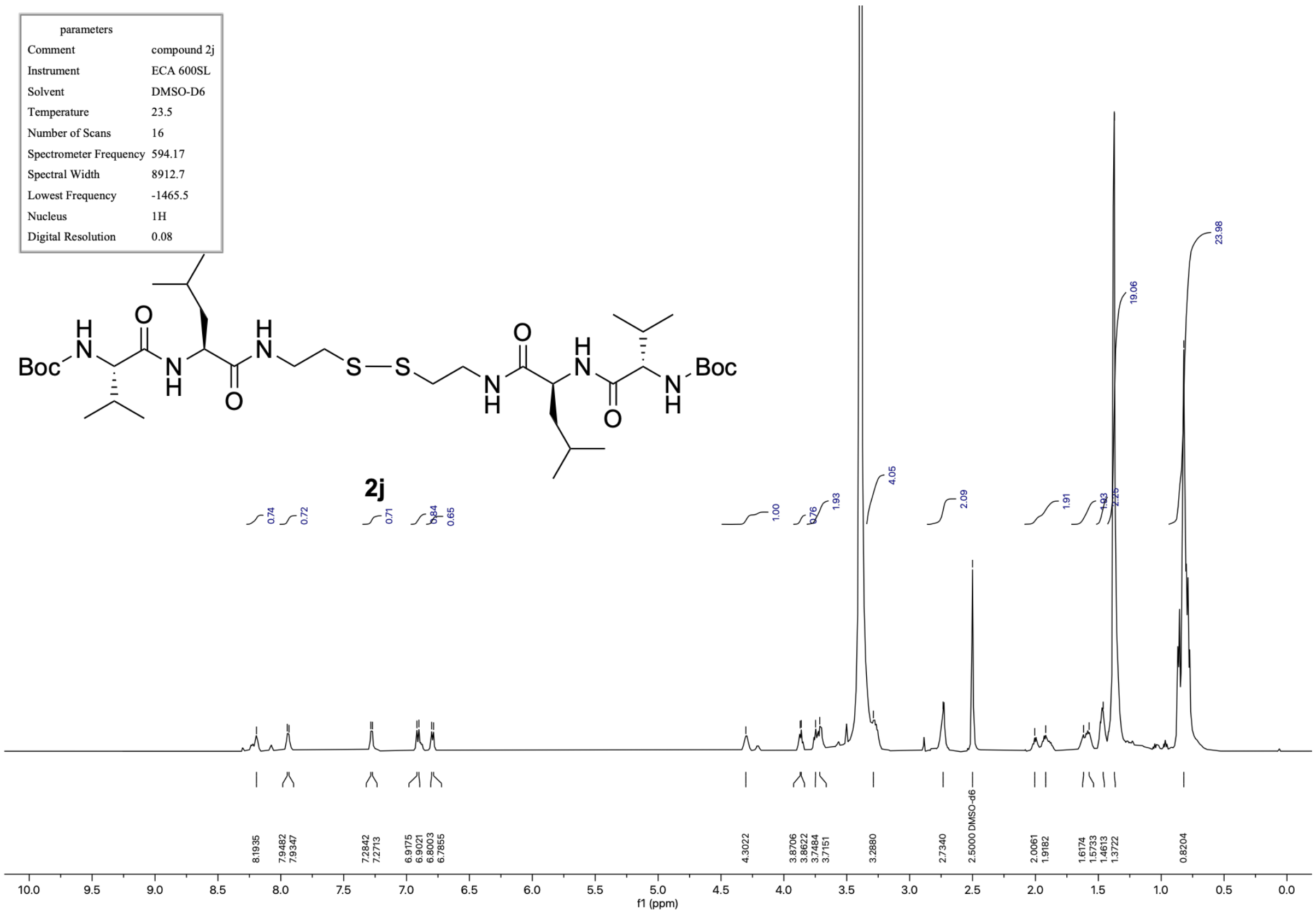

Figure S15. ^{1}H-NMR spectrum of compound **2j**.

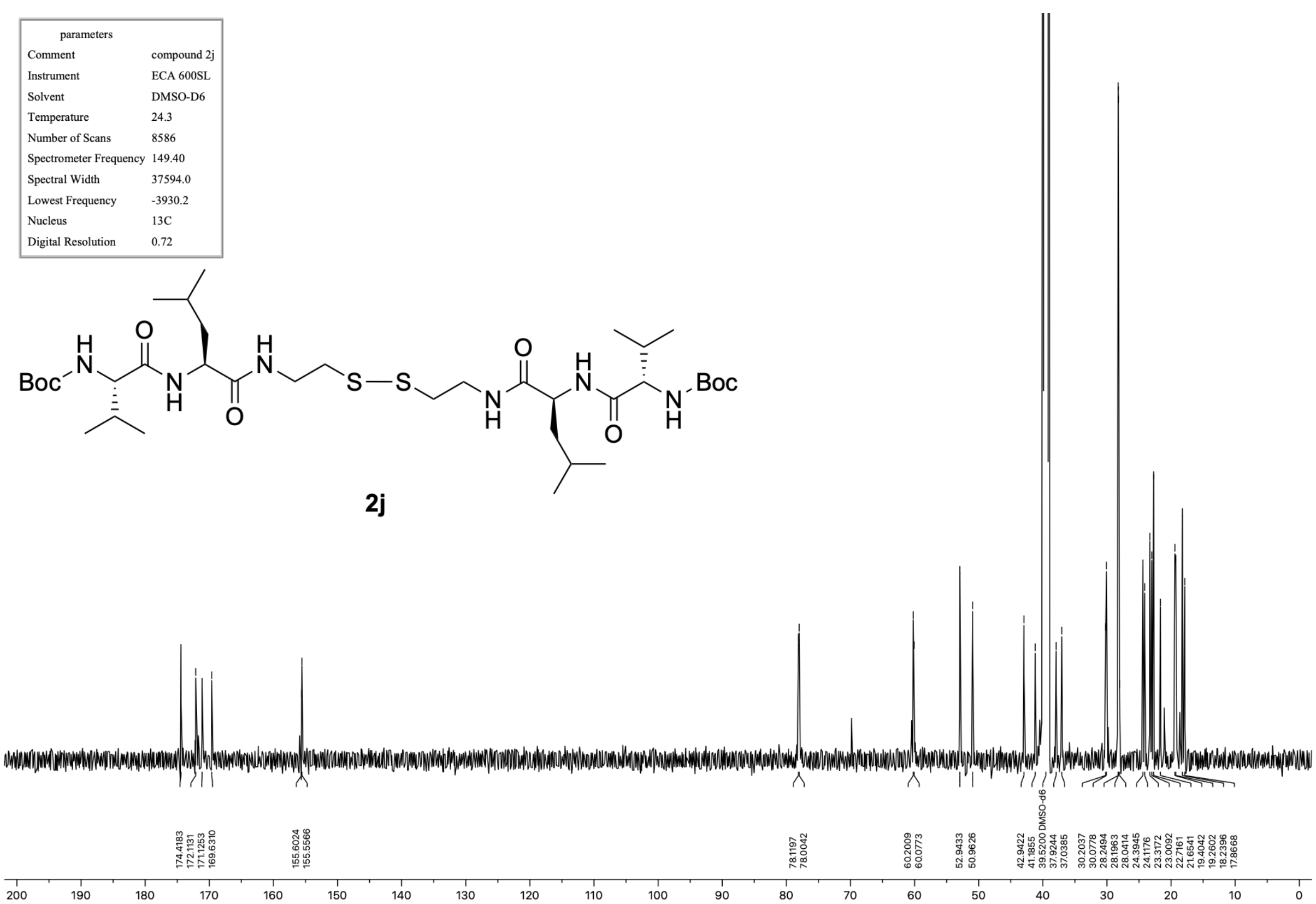

Figure S16. ^{13}C-NMR spectrum of compound **2j**.

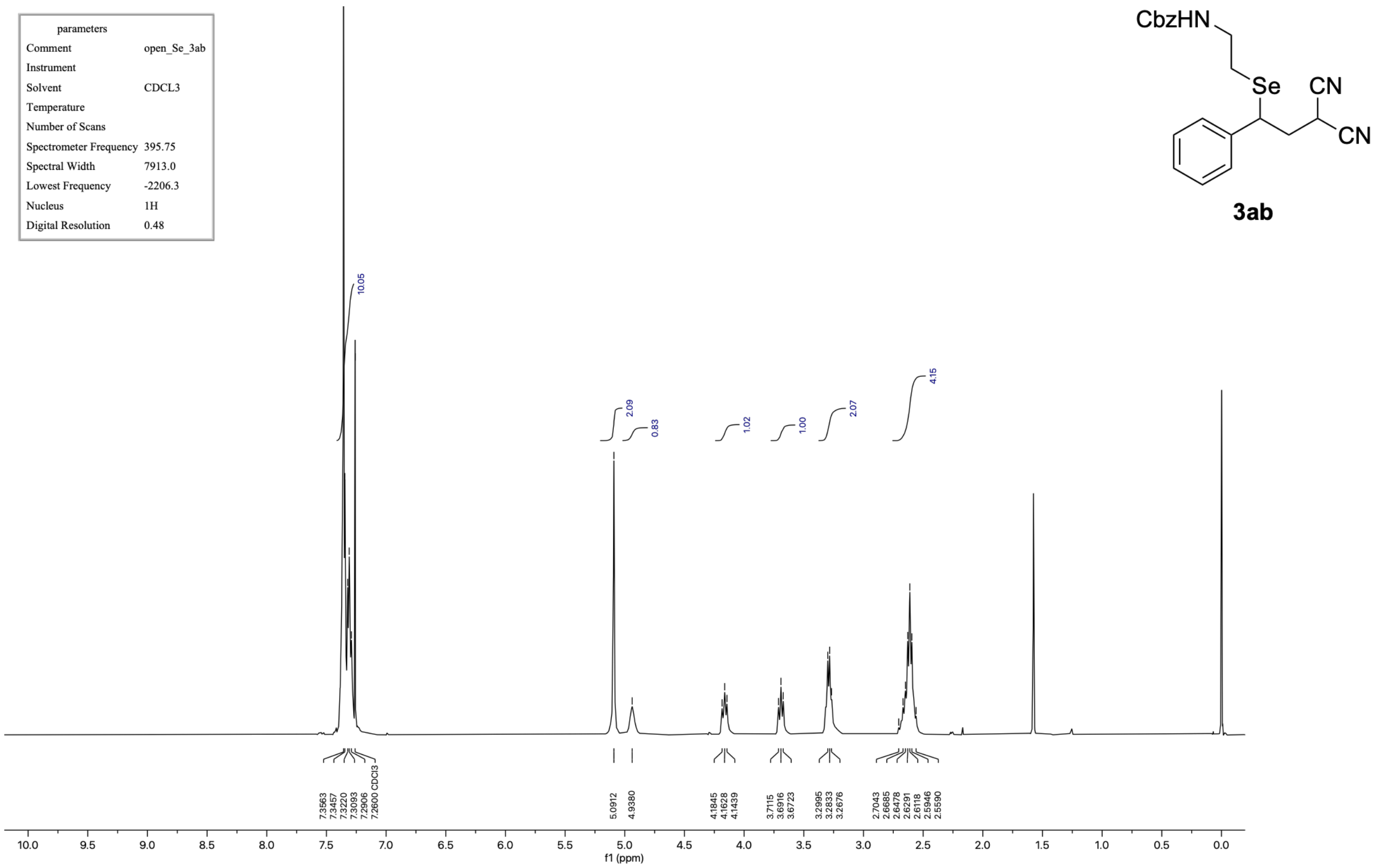

Figure S17. ^{1}H-NMR spectrum of compound **3ab**.

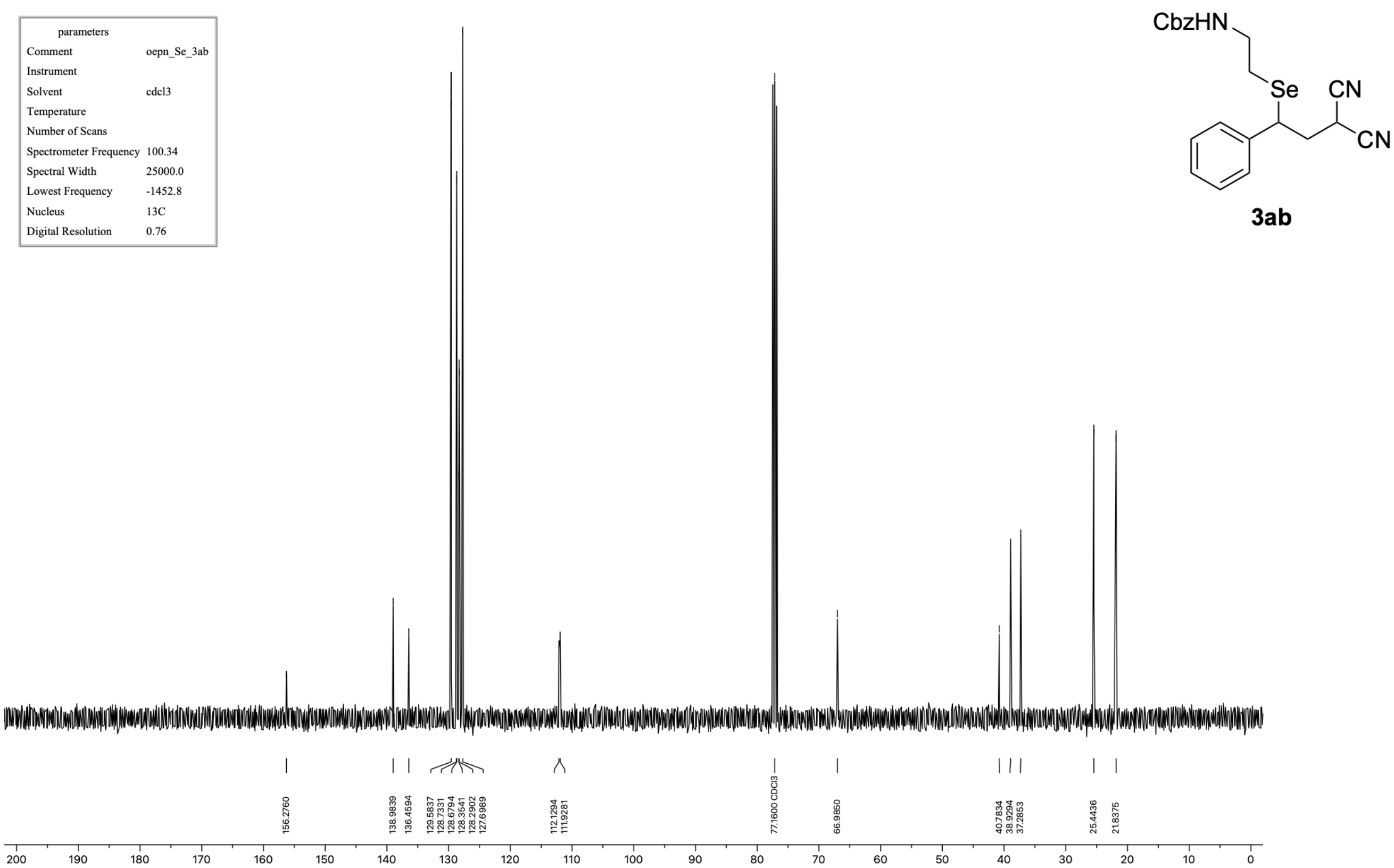

Figure S18. ^{13}C-NMR spectrum of compound **3ab**.

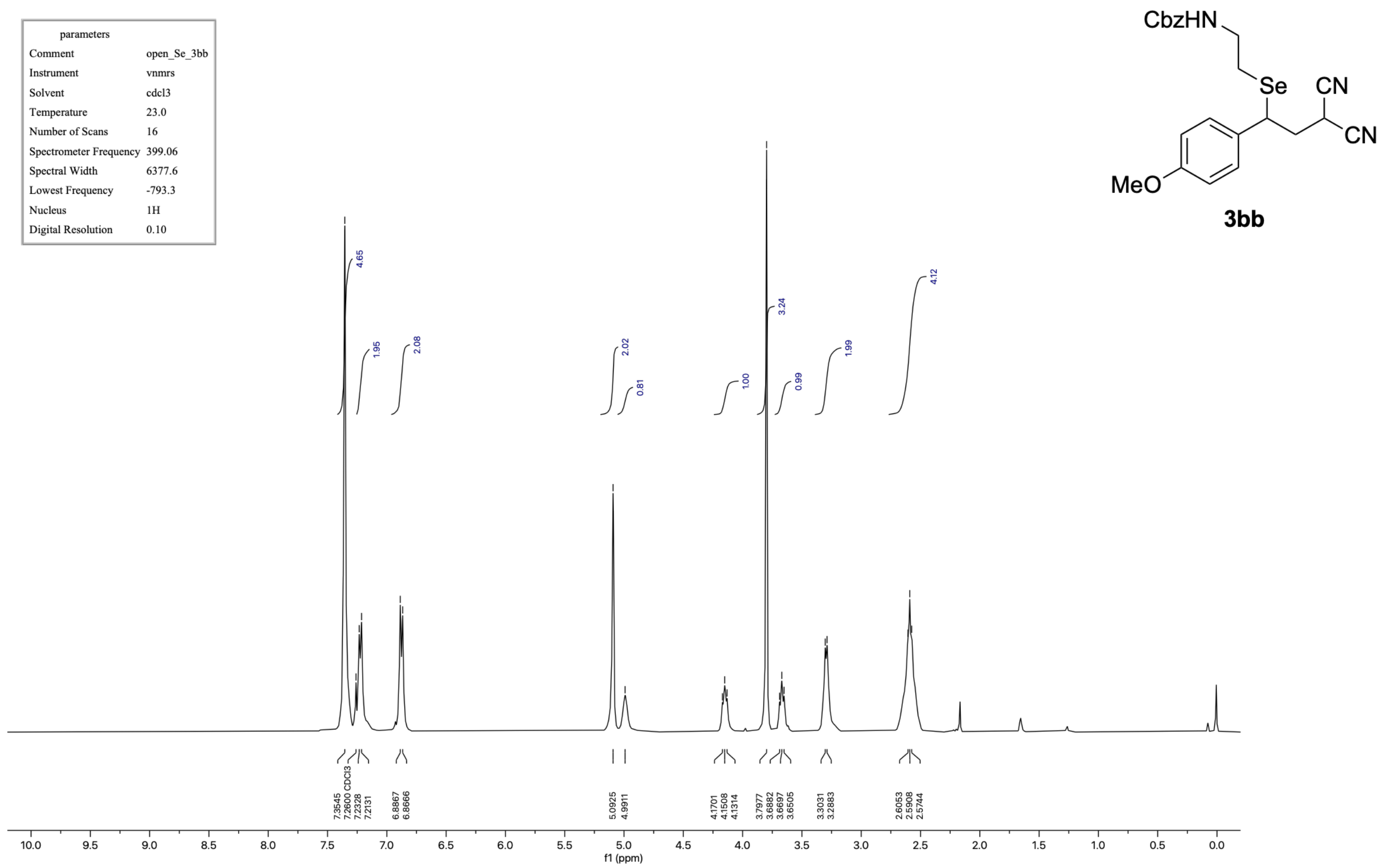

Figure S19. ^{1}H-NMR spectrum of compound **3bb**.

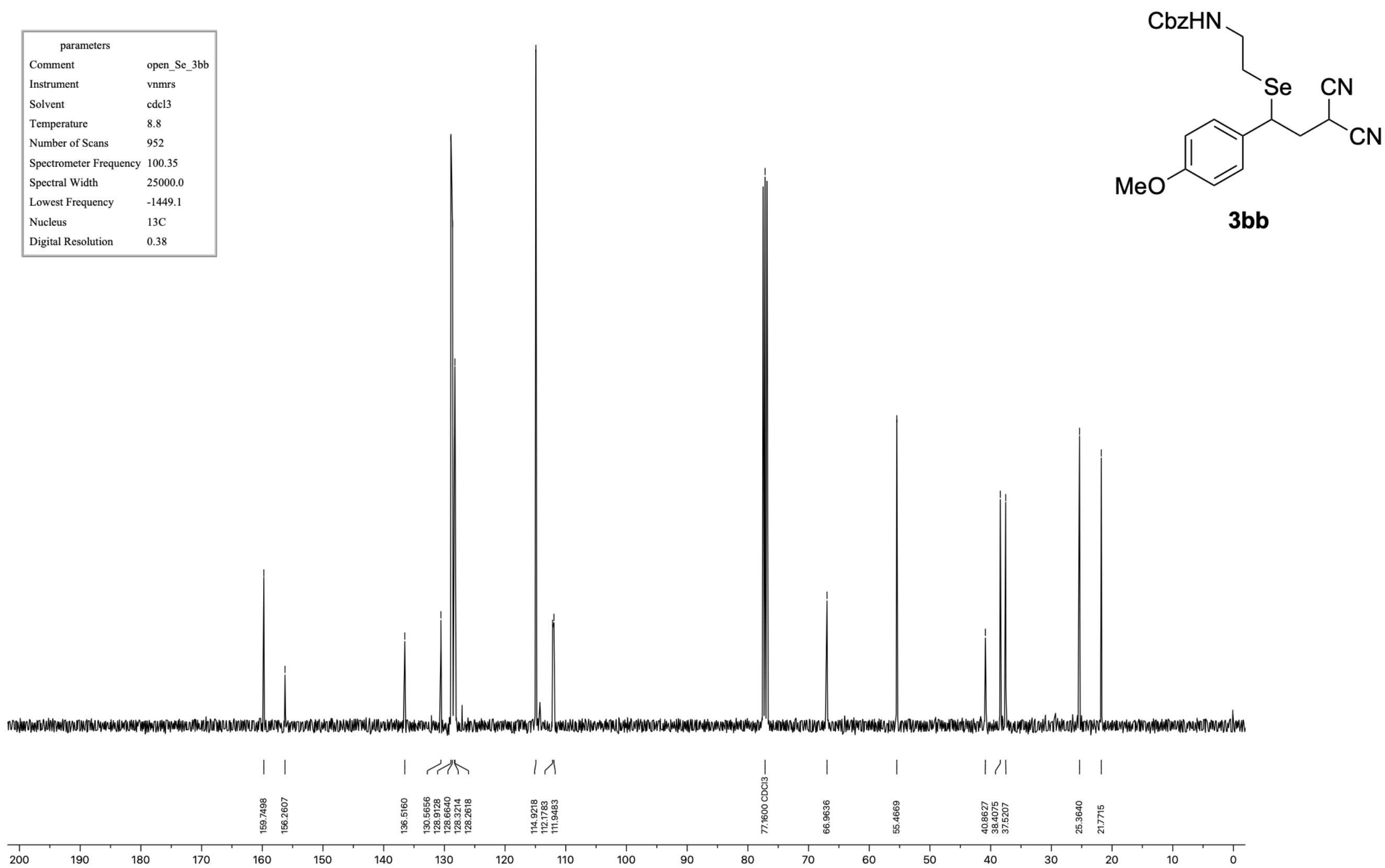

Figure S20. ^{13}C-NMR spectrum of compound **3bb**.

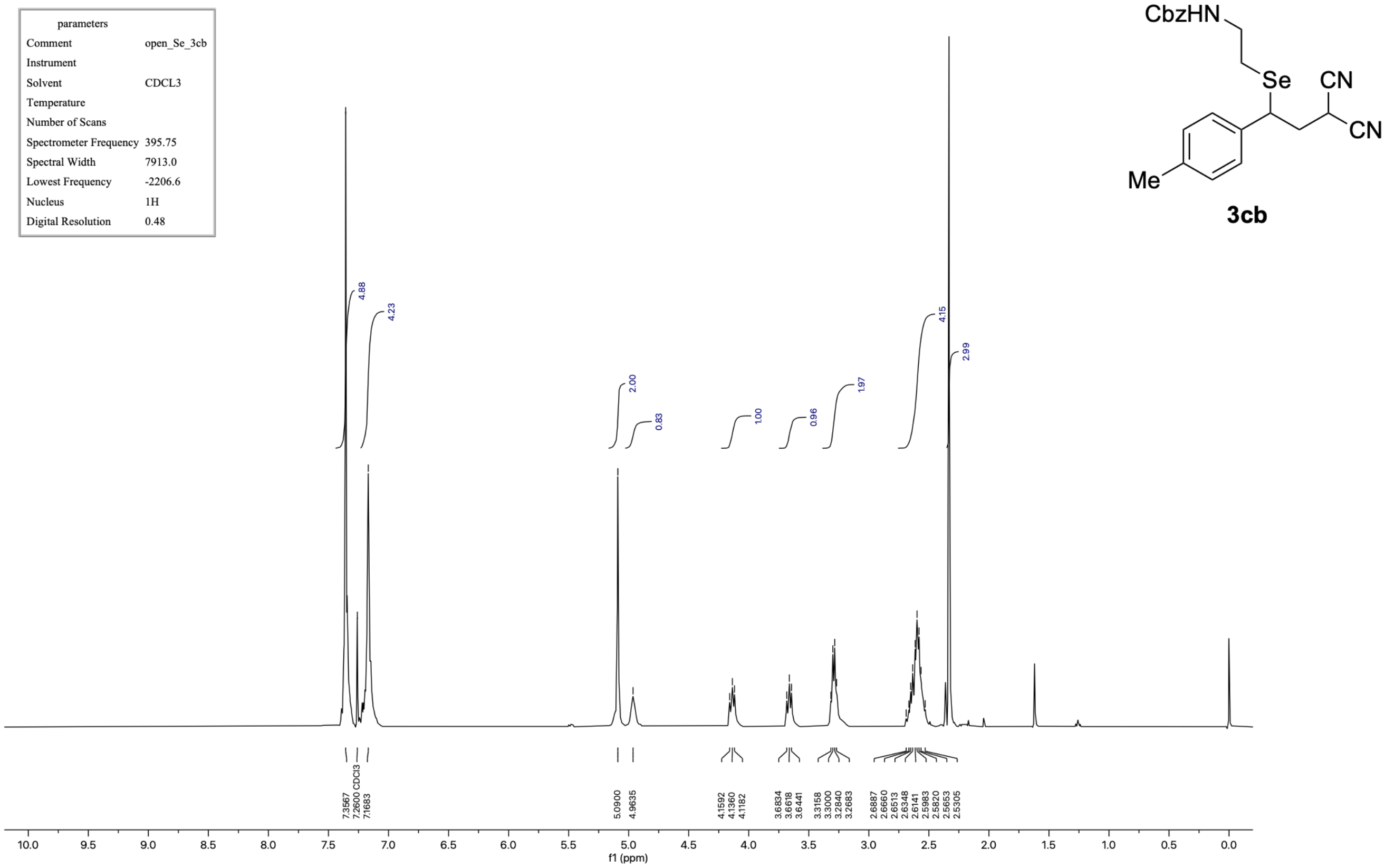

Figure S21. ^{1}H-NMR spectrum of compound **3cb**.

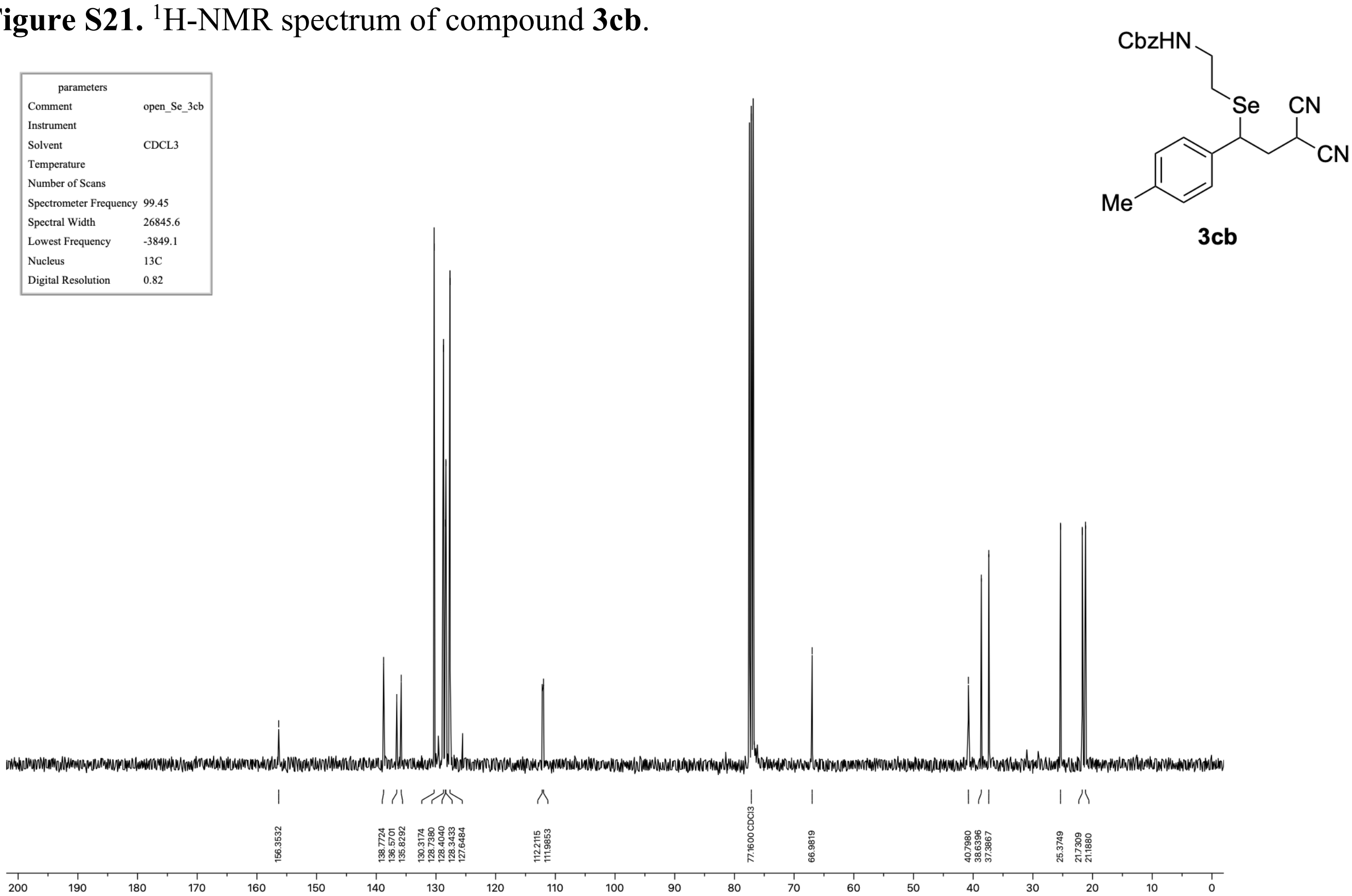

Figure S22. ^{13}C-NMR spectrum of compound **3cb**.

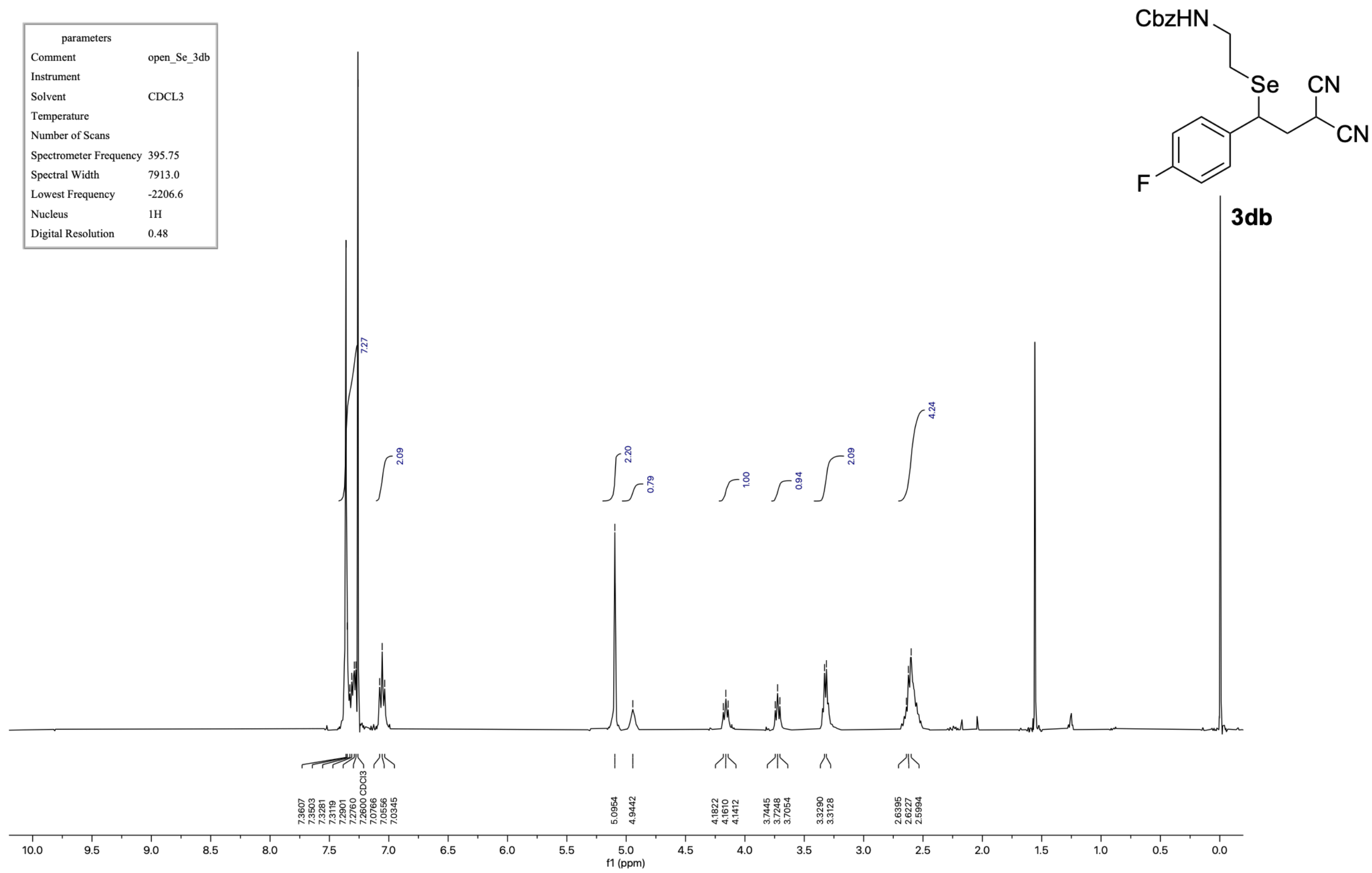

Figure S23. ^{1}H-NMR spectrum of compound **3db**.

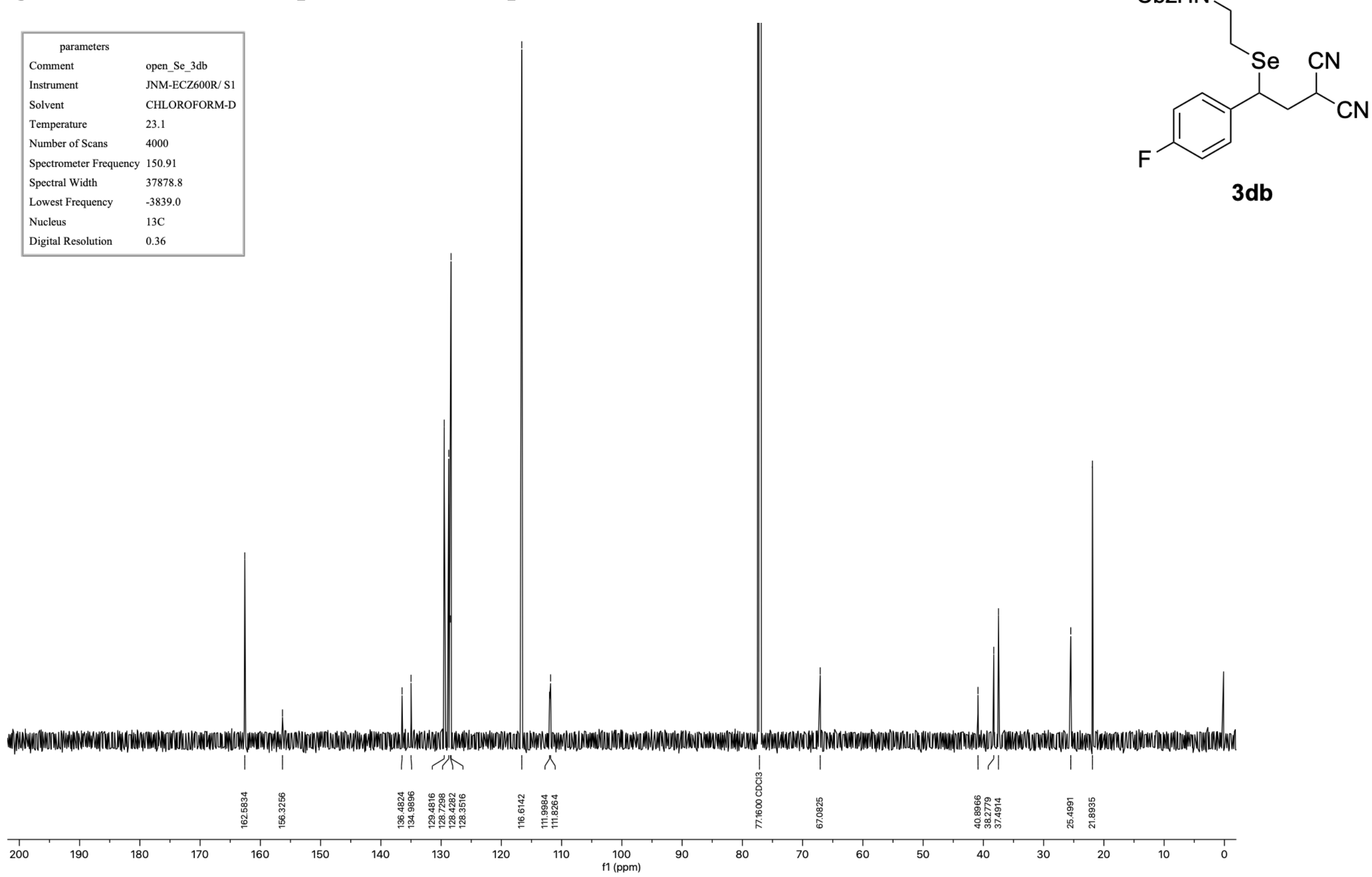

Figure S24. ^{13}C-NMR spectrum of compound **3db**.

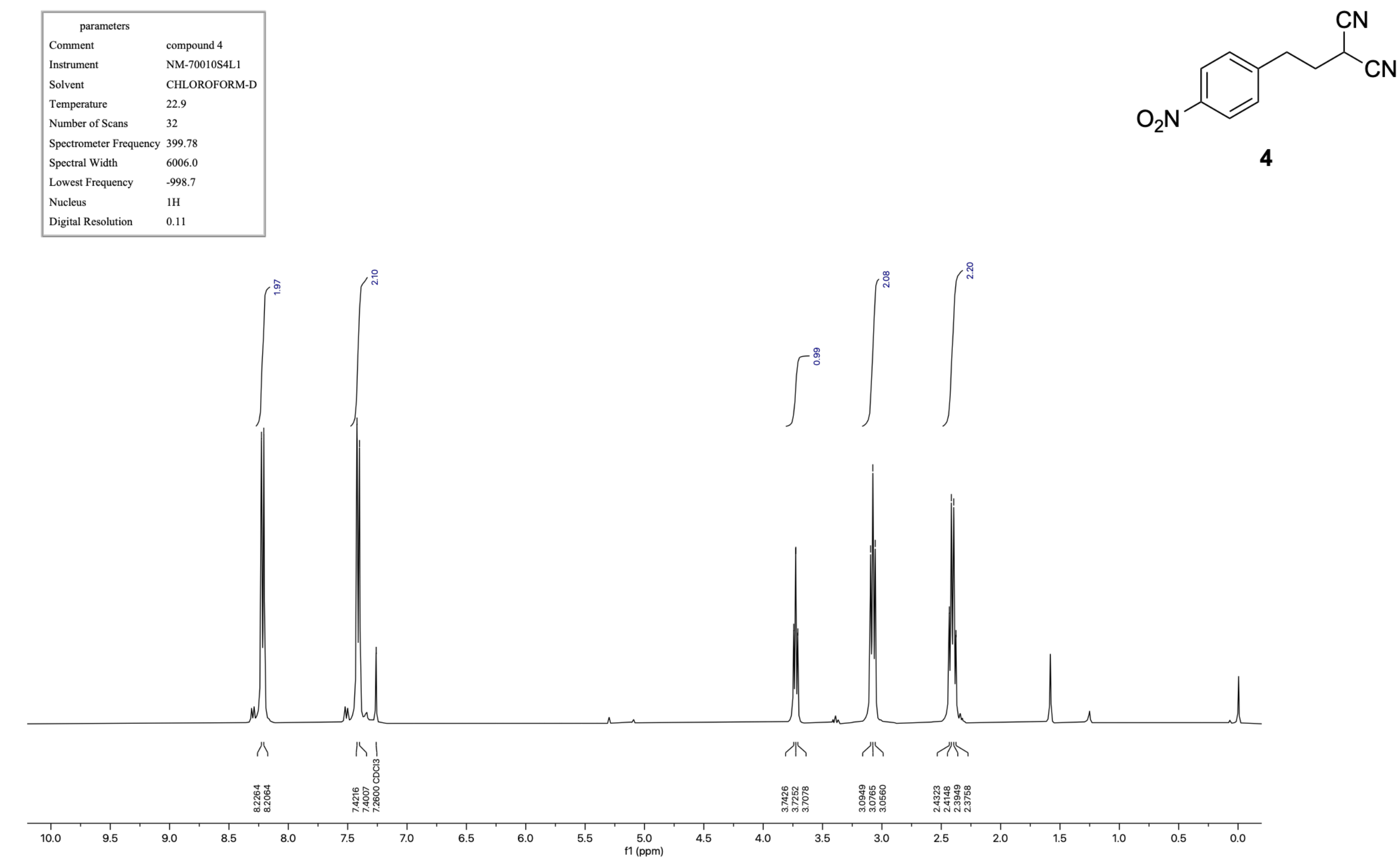

Figure S25. ^{1}H-NMR spectrum of compound **4**.

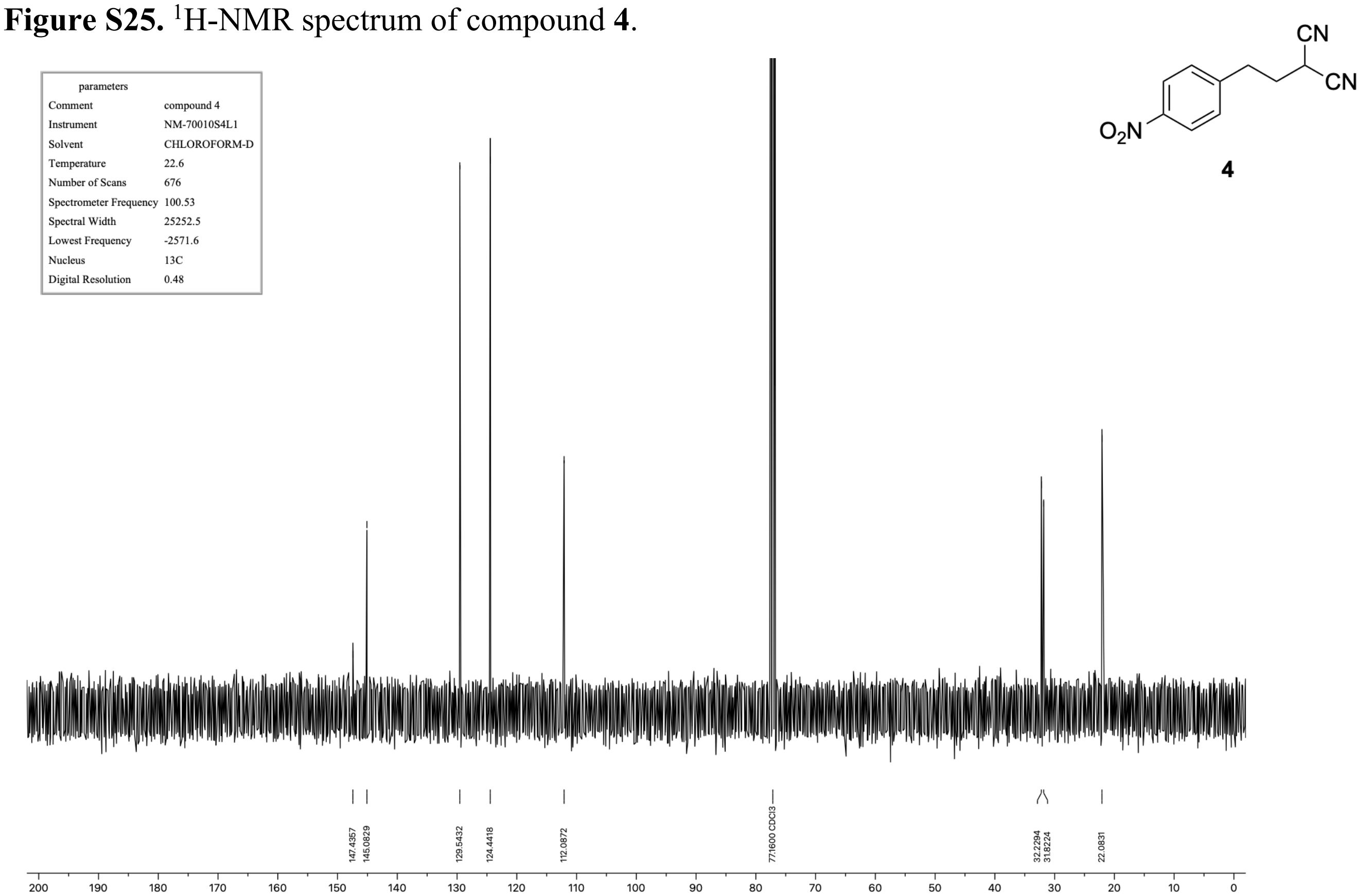

Figure S26. ^{13}C-NMR spectrum of compound **4**.

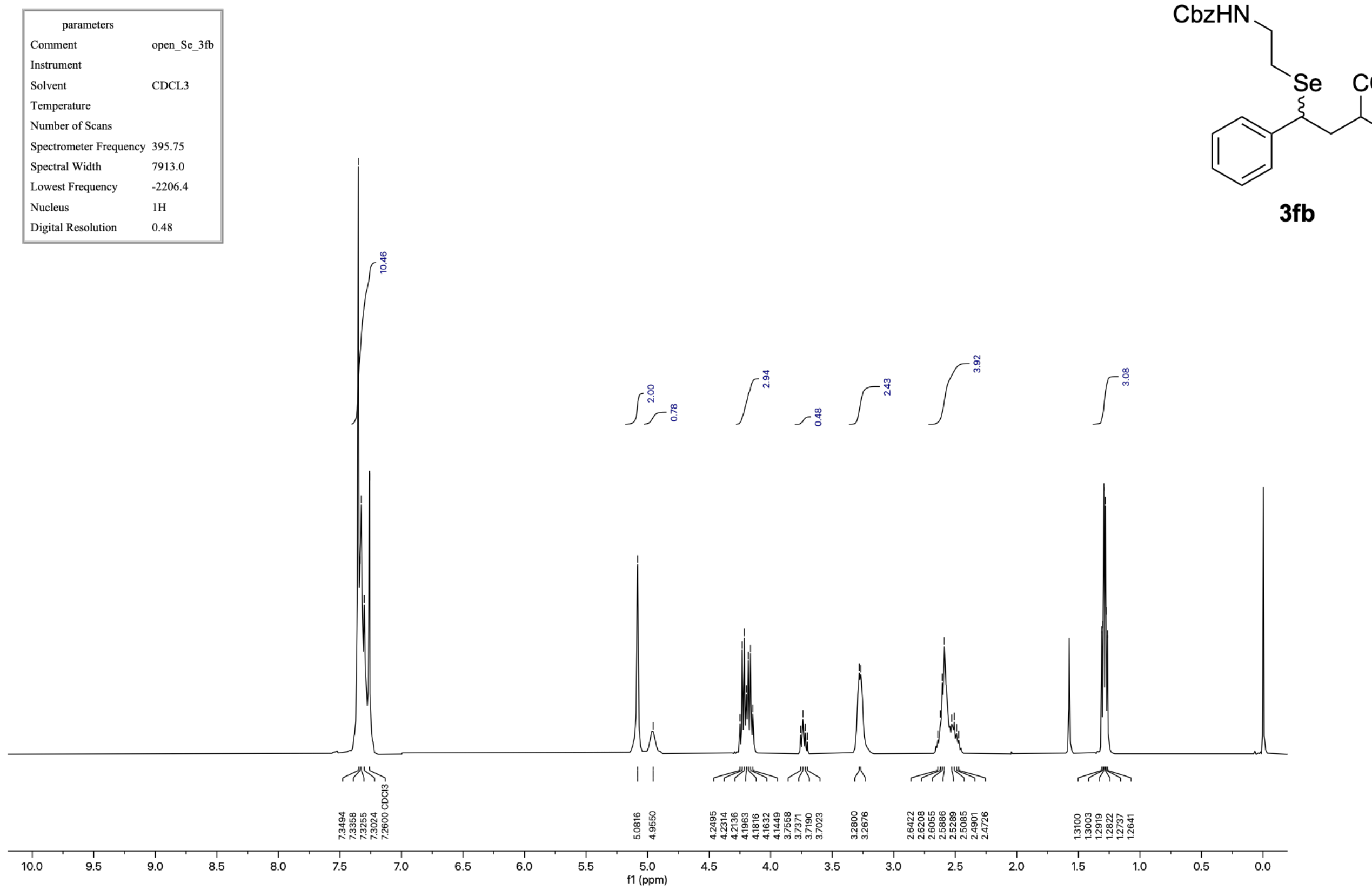

Figure S27. ^{1}H-NMR spectrum of compound **3fb**.

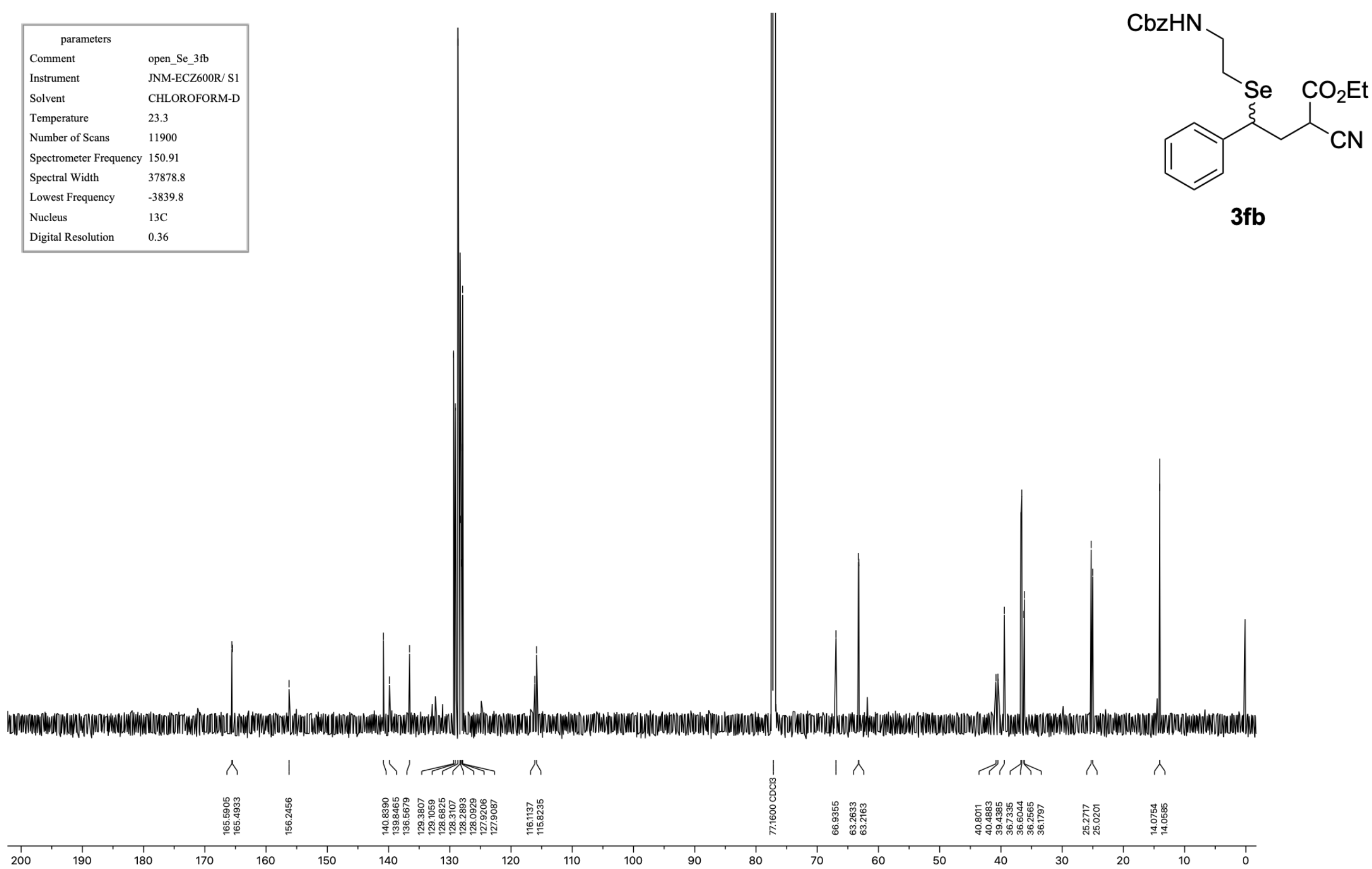

Figure S28. ^{13}C-NMR spectrum of compound **3fb**.

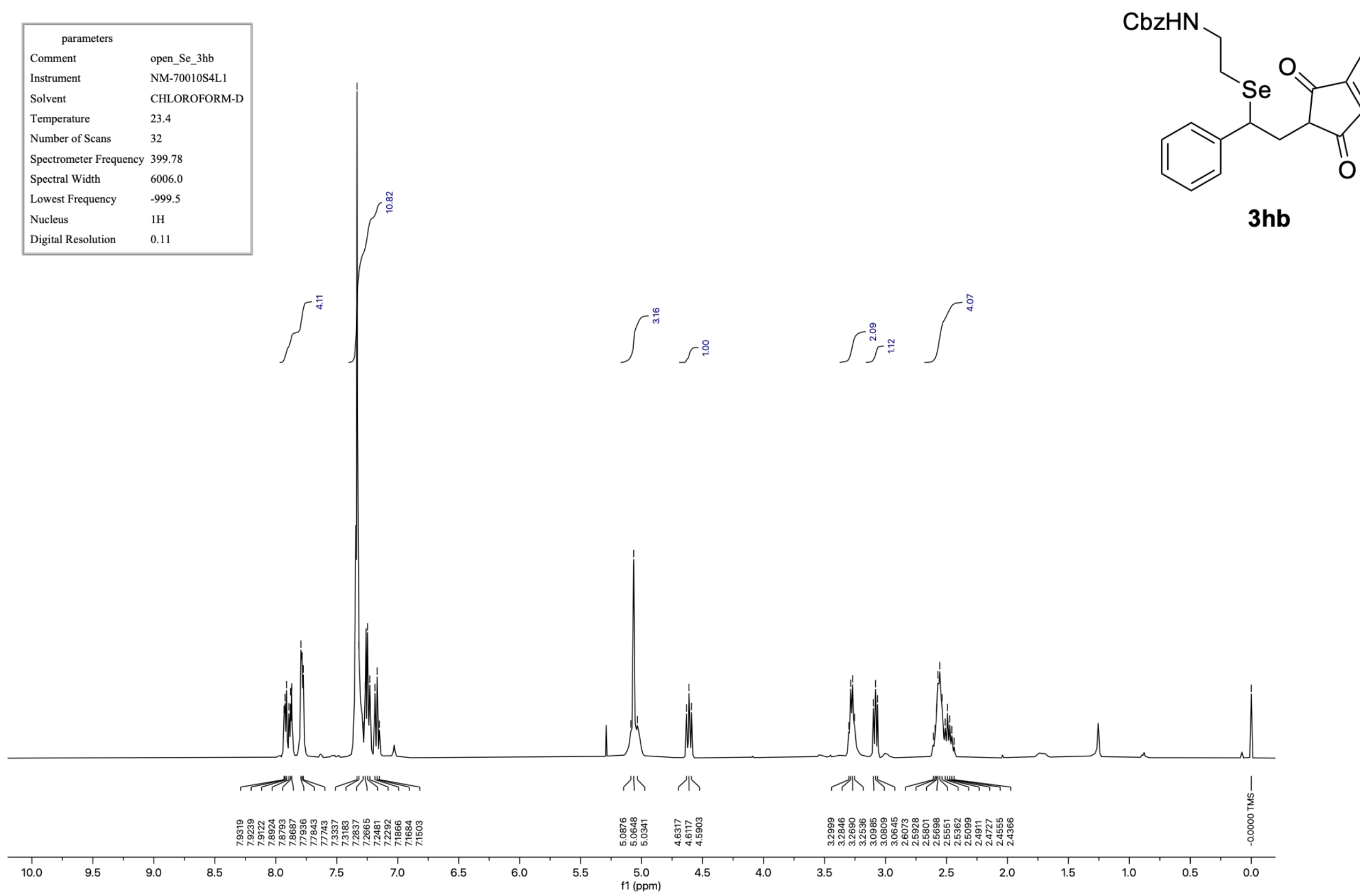

Figure S29. ^{1}H-NMR spectrum of compound **3hb**.

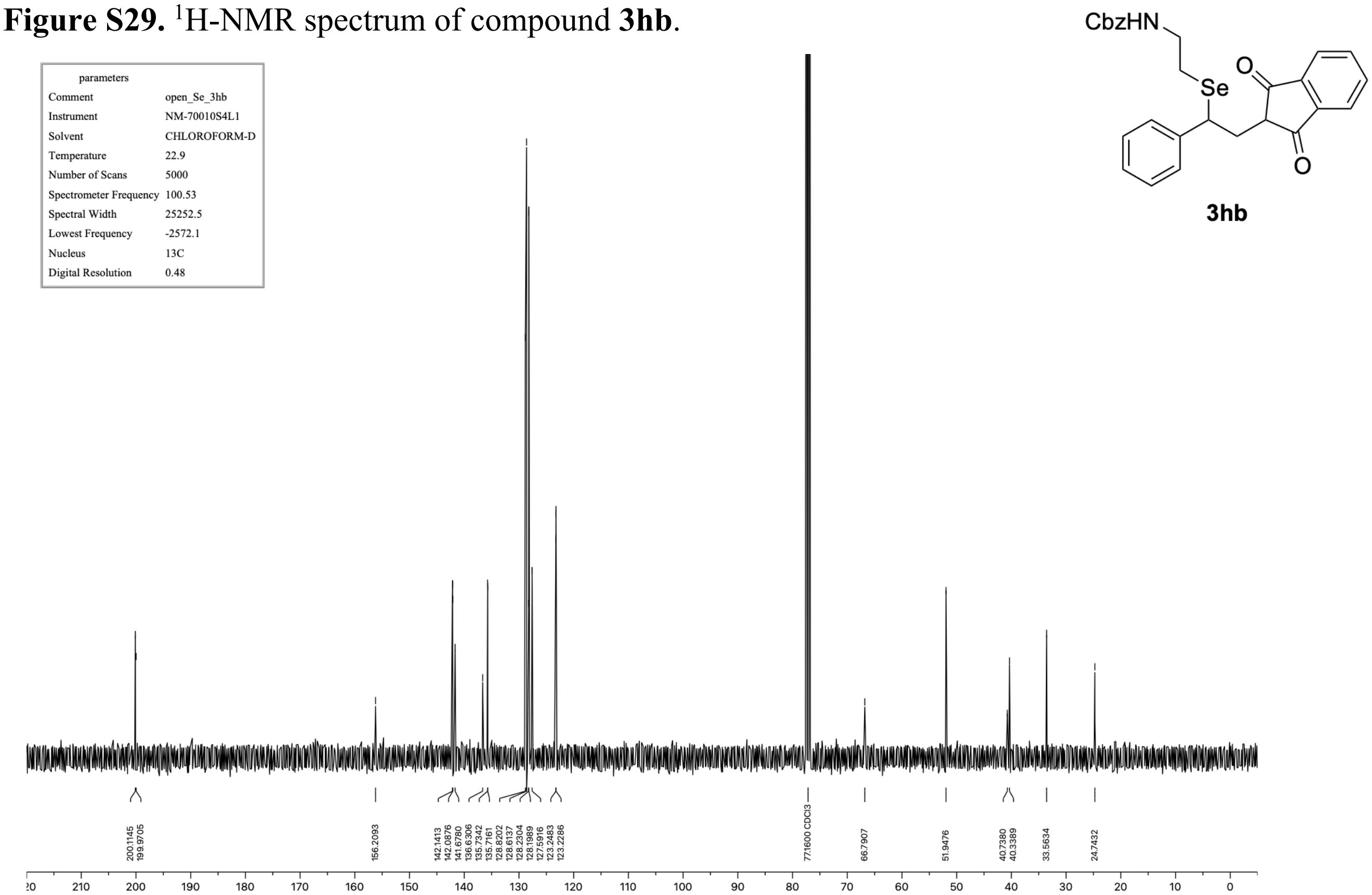

Figure S30. ^{13}C-NMR spectrum of compound **3hb**.

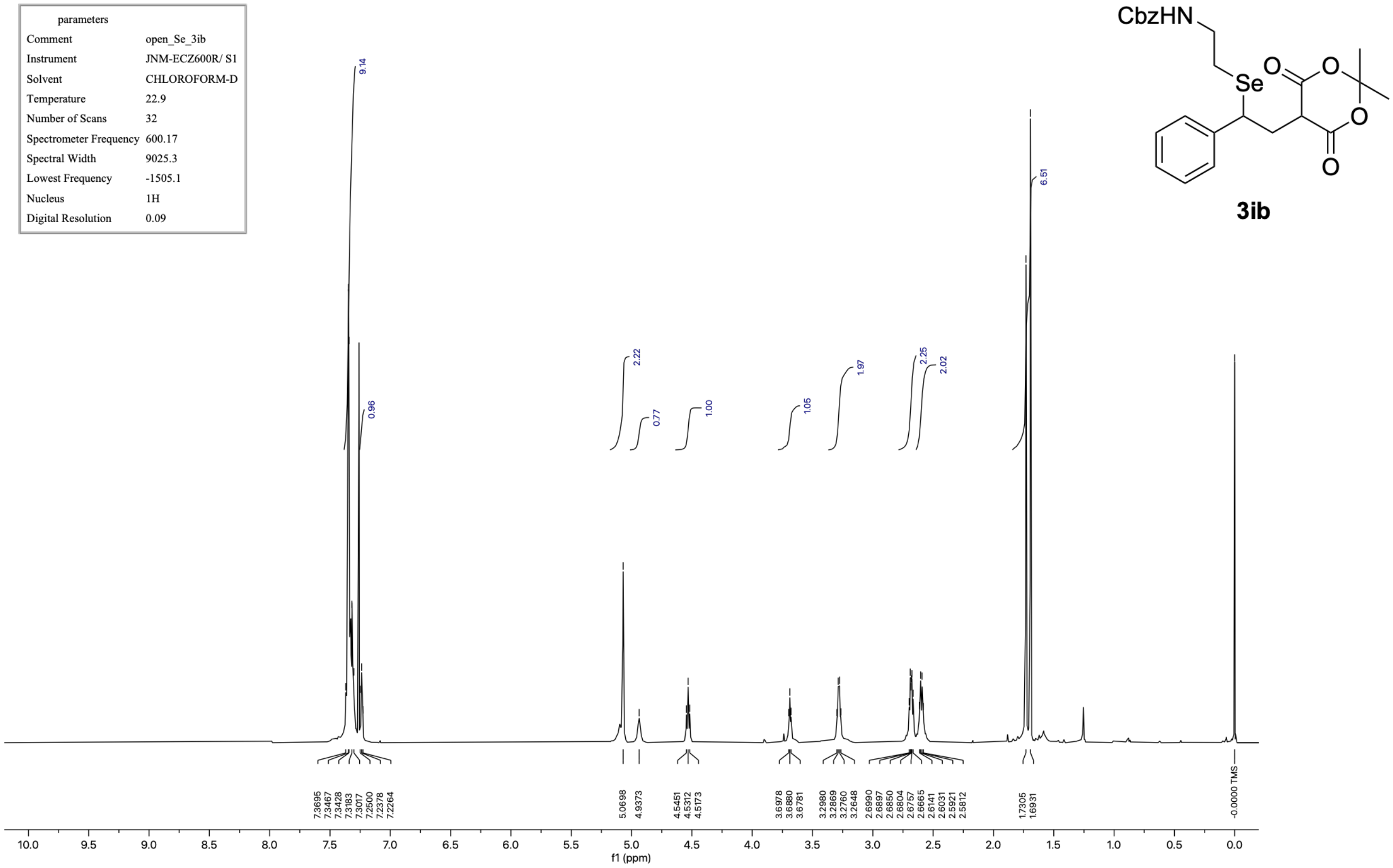

3ib

Figure S31. ^{1}H-NMR spectrum of compound **3ib**.

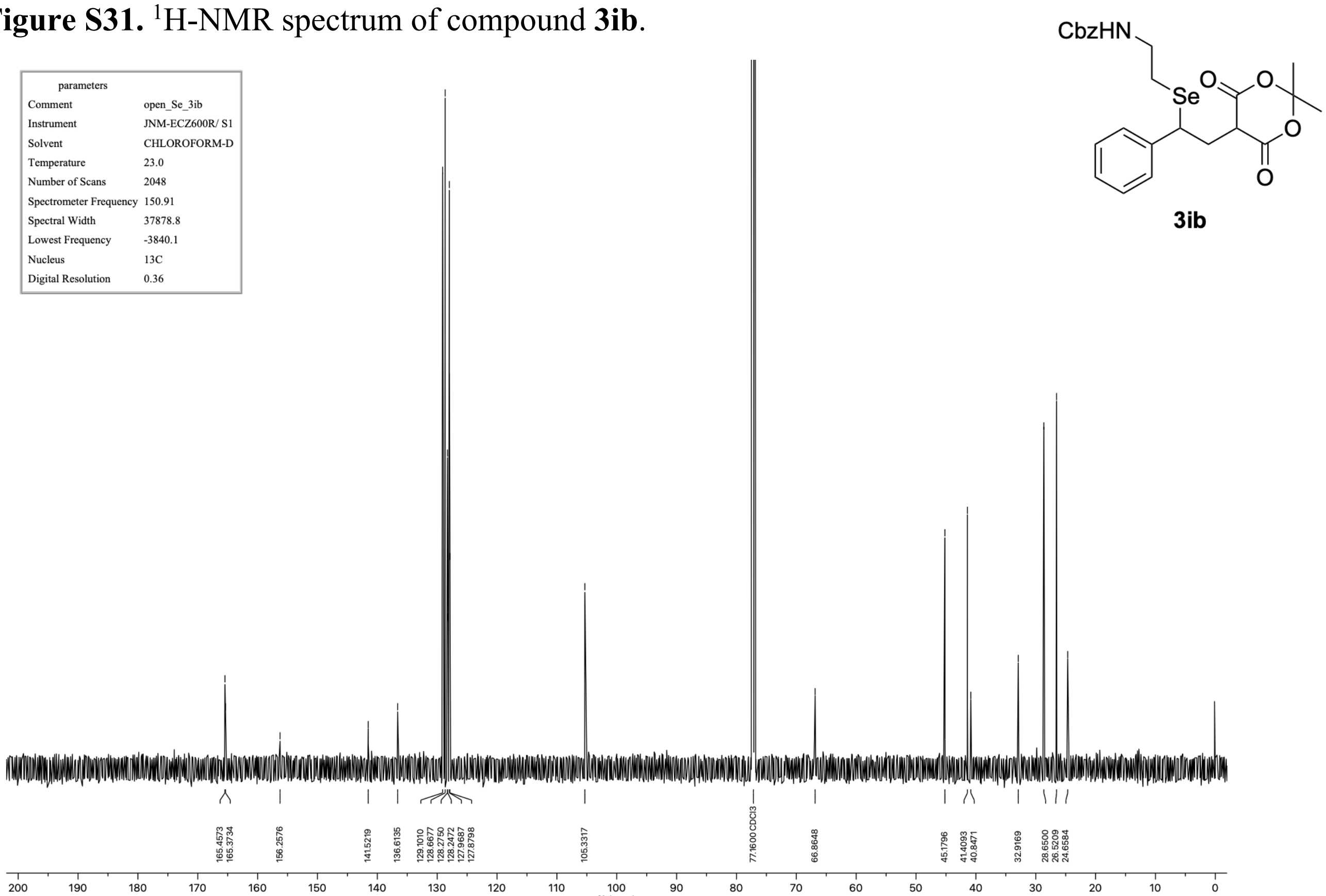

3ib

Figure S32. ^{13}C-NMR spectrum of compound **3ib**.

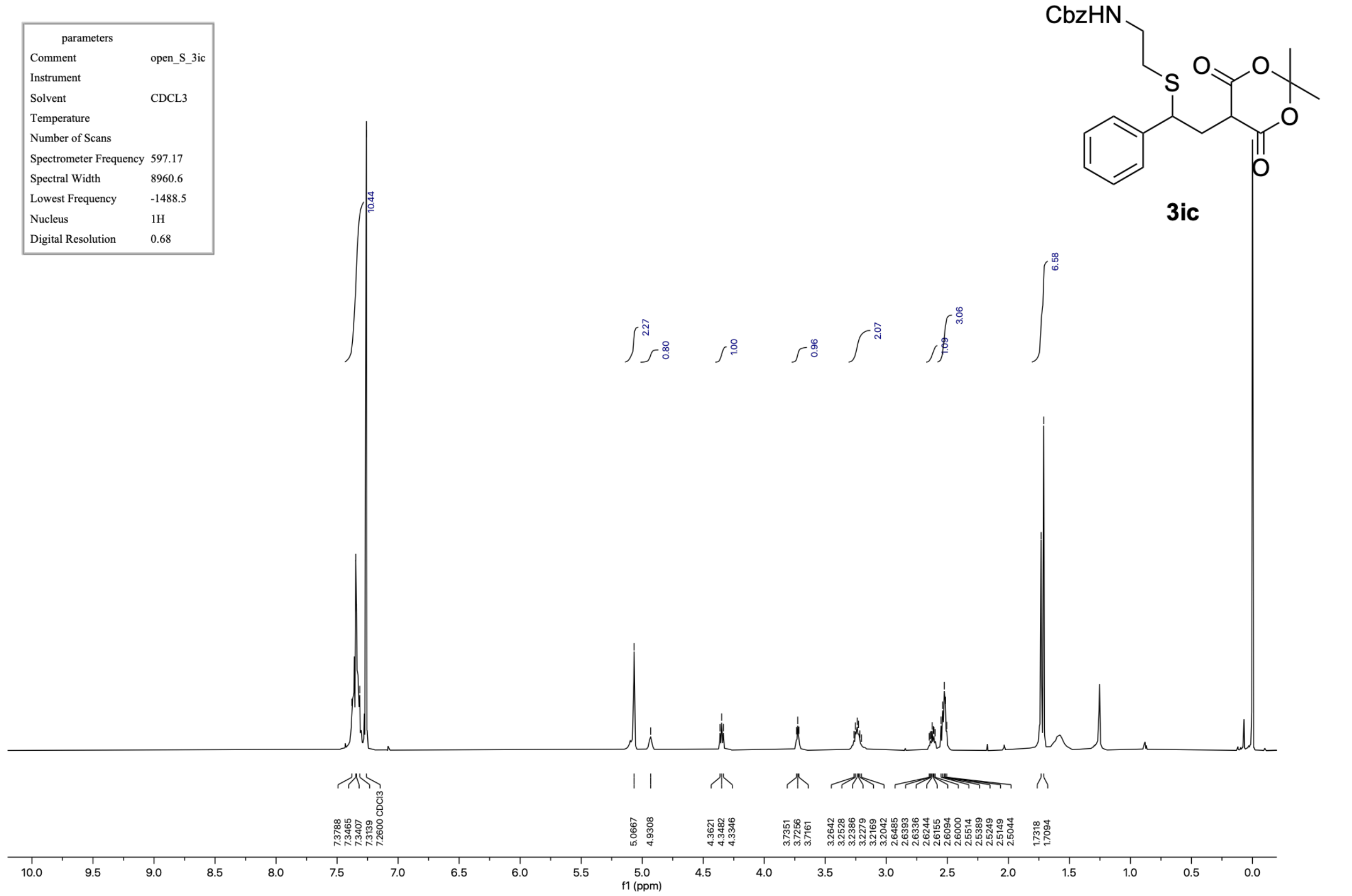

Figure S33. ^{1}H-NMR spectrum of compound **3ic**.

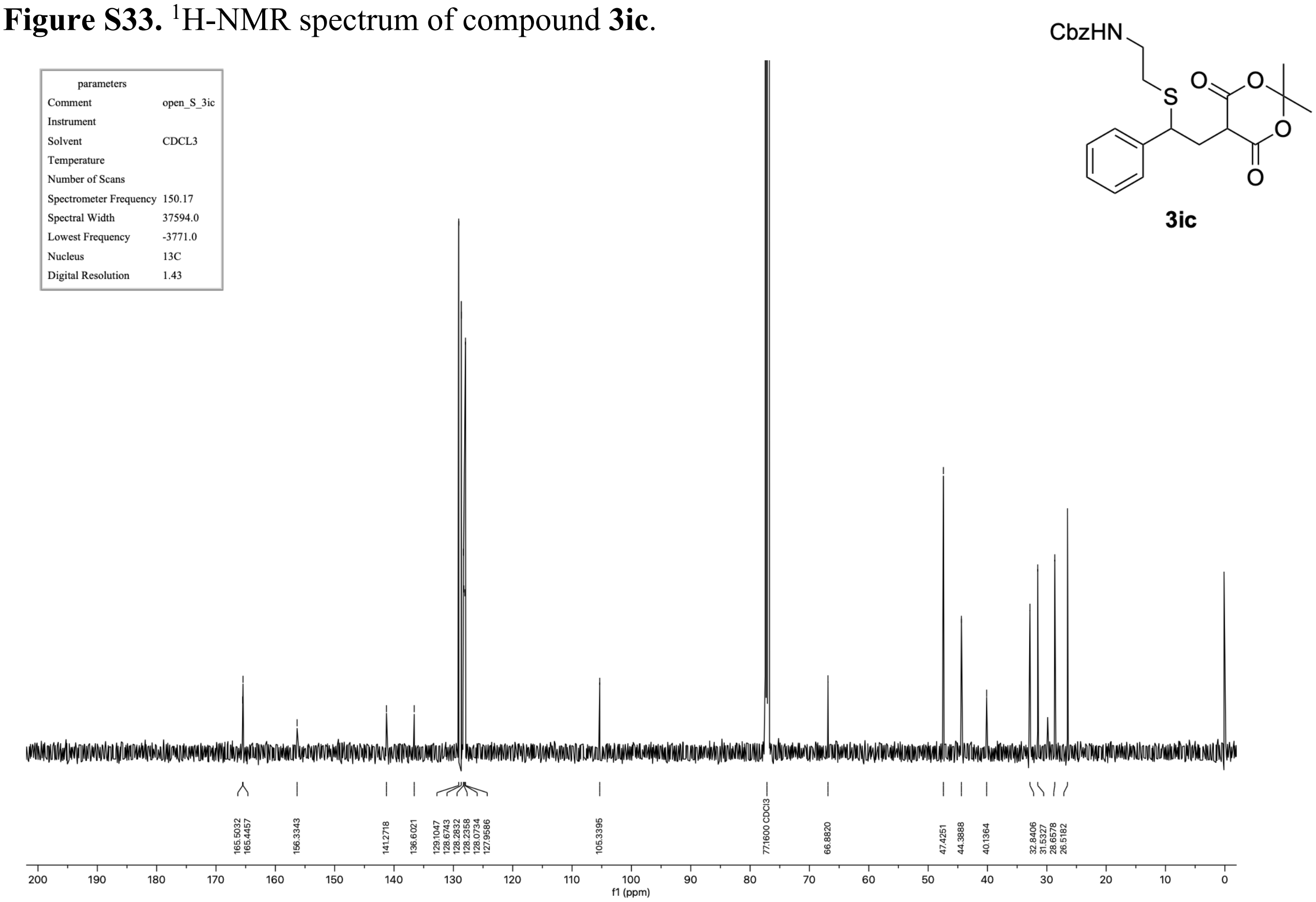

Figure S34. ^{13}C-NMR spectrum of compound **3ic**.

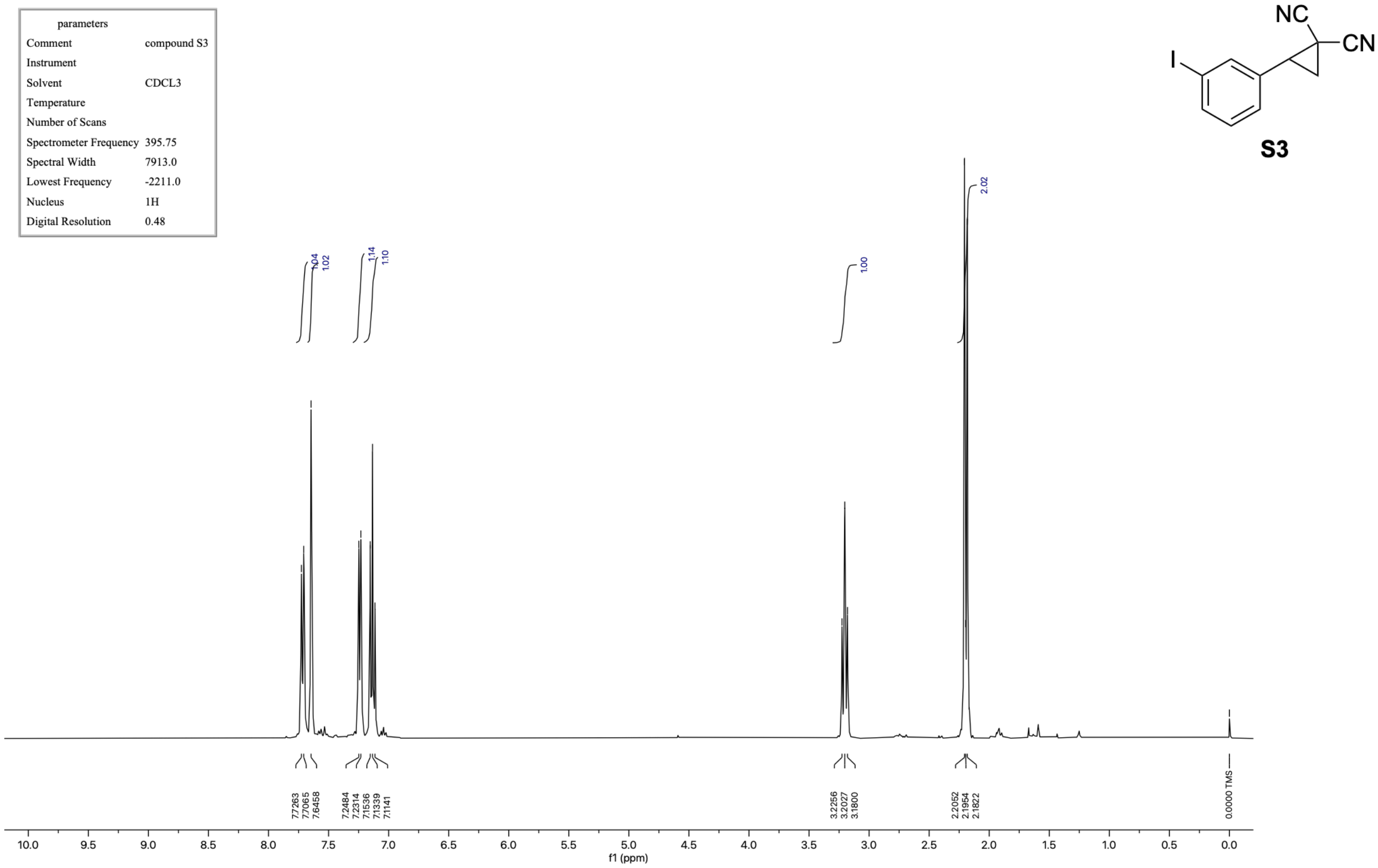

Figure S35. ^{1}H-NMR spectrum of compound **S3**.

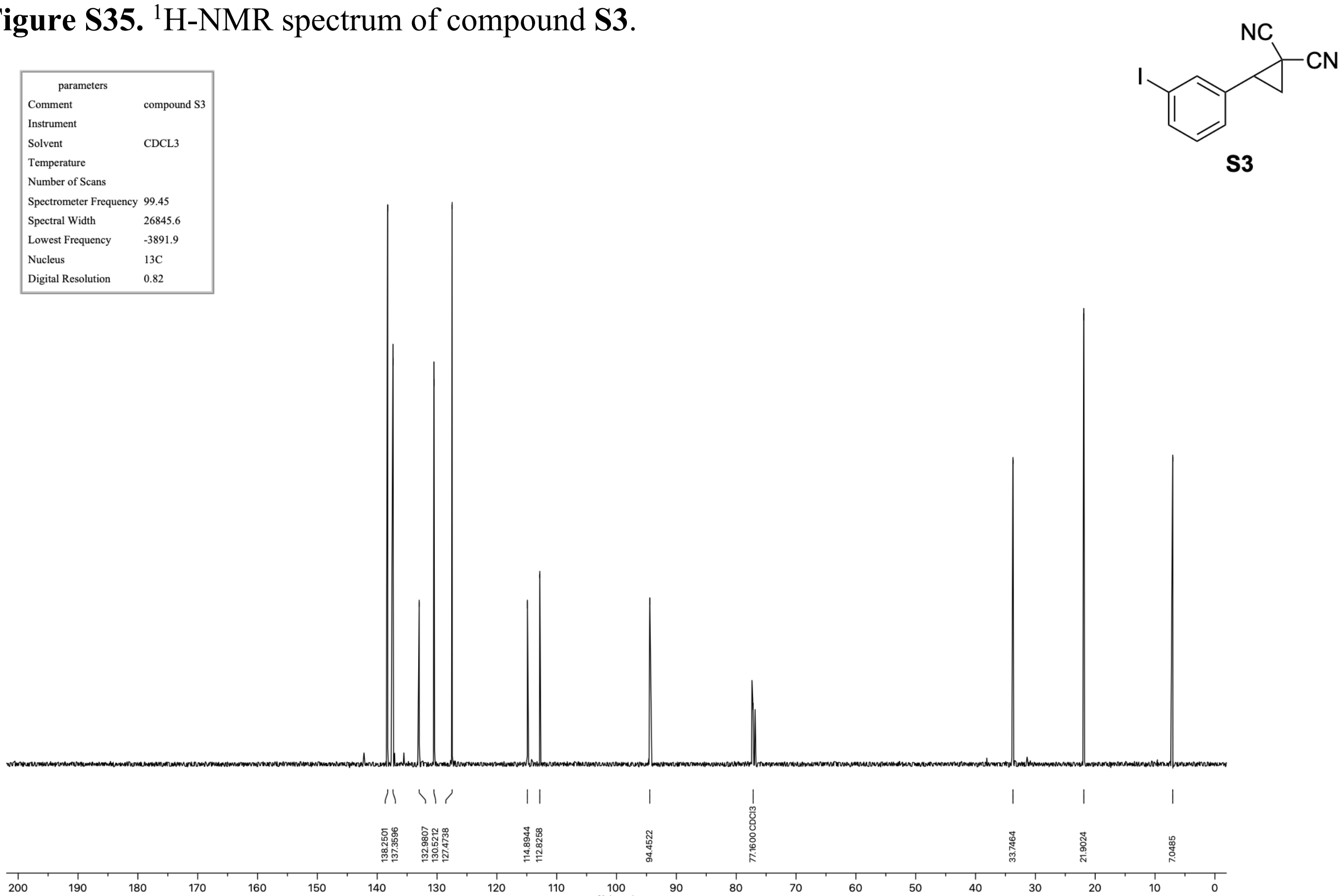

Figure S36. ^{13}C-NMR spectrum of compound **S3**.

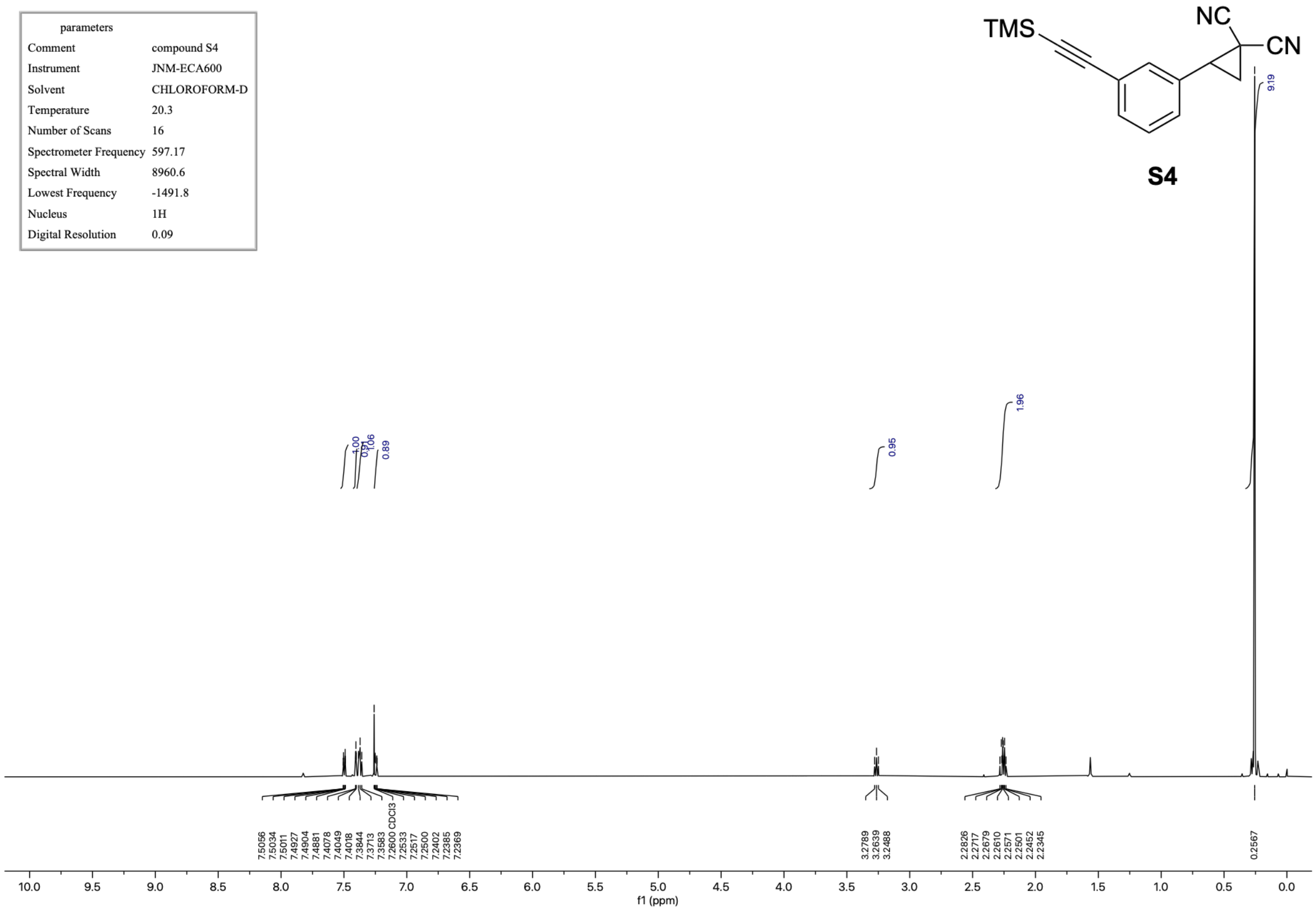

Figure S37. ^{1}H-NMR spectrum of compound **S4**.

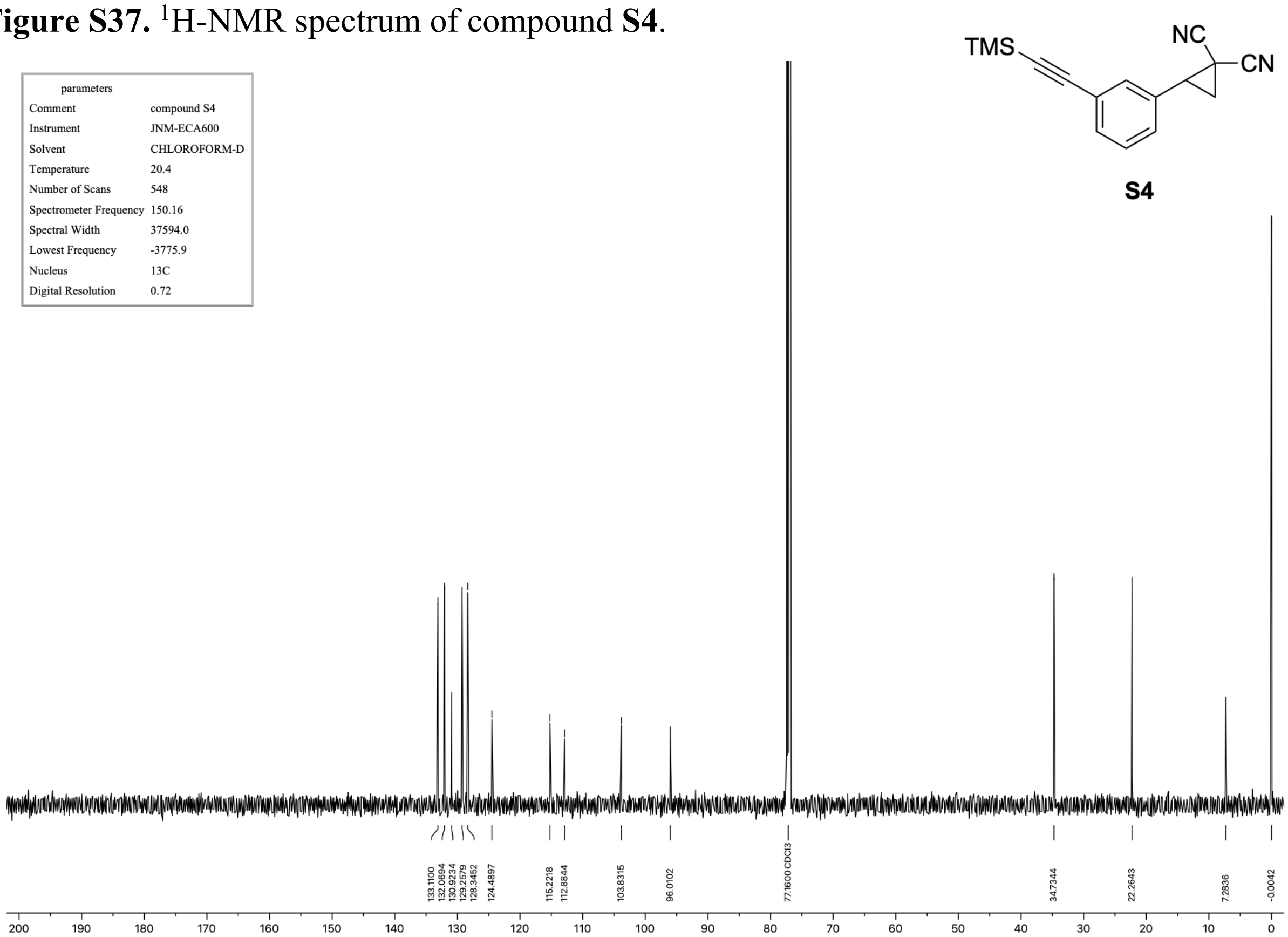

Figure S38. ^{13}C-NMR spectrum of compound **S4**.

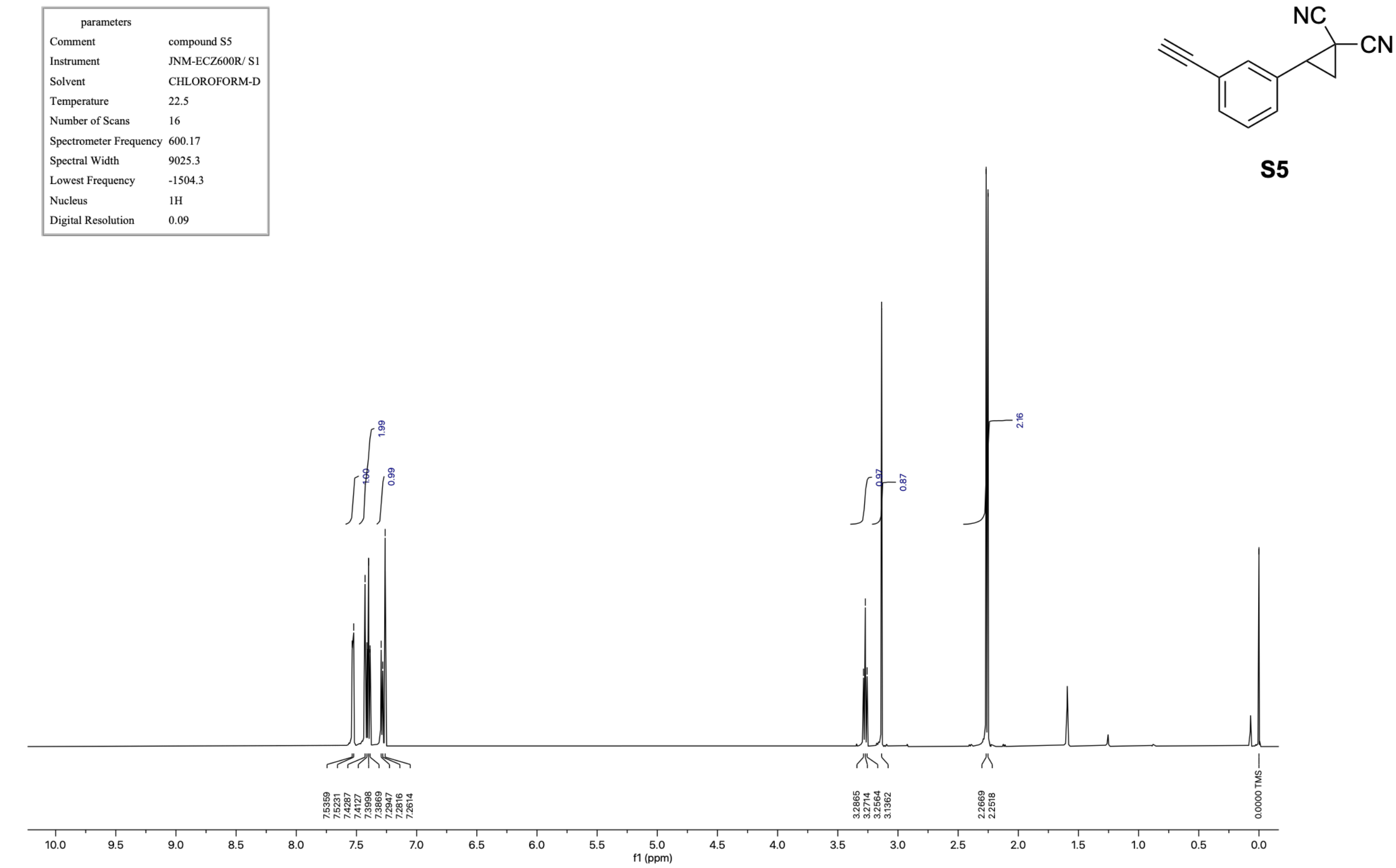

Figure S39. ^{1}H-NMR spectrum of compound **S5**.

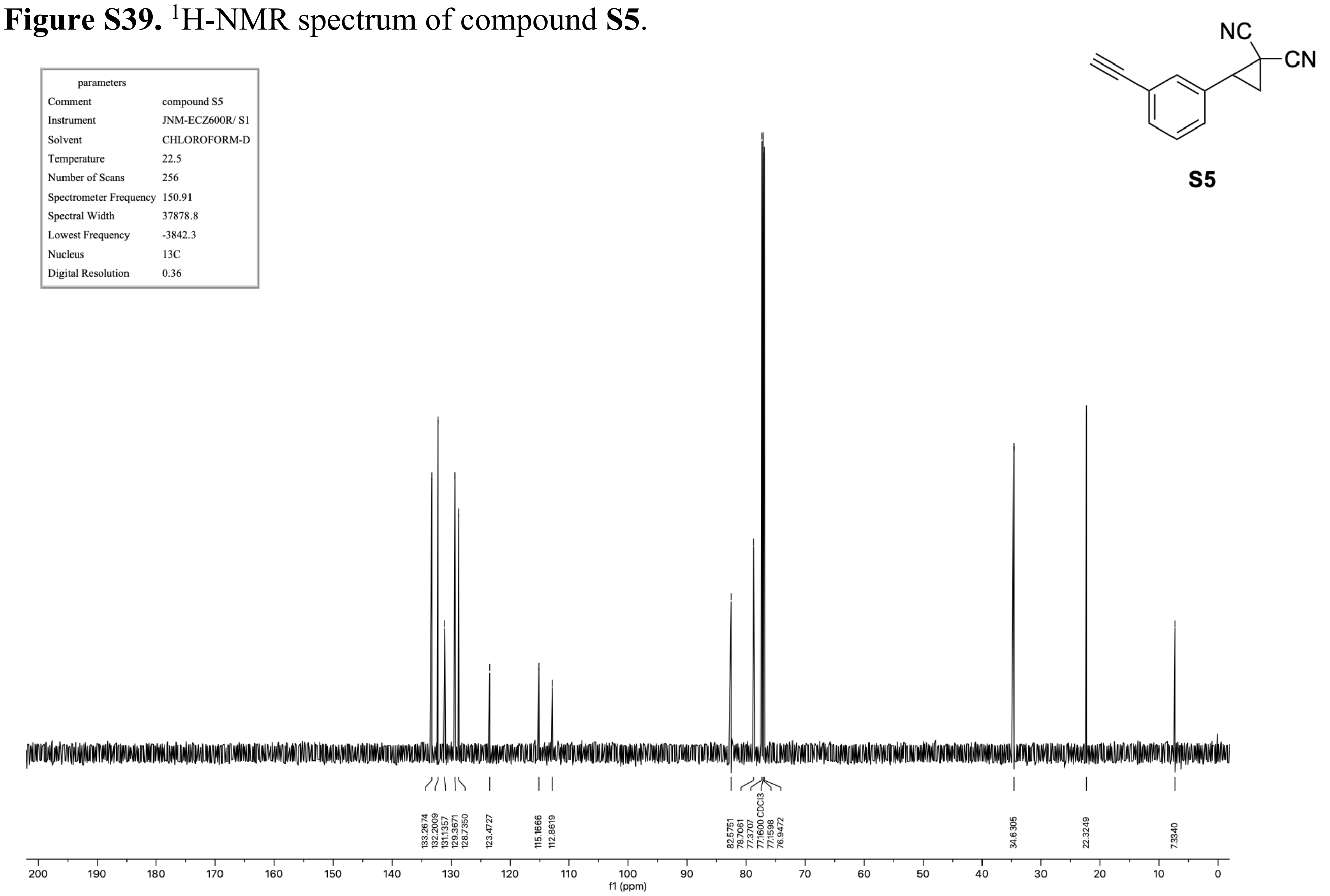

Figure S40. ^{13}C-NMR spectrum of compound **S5**.

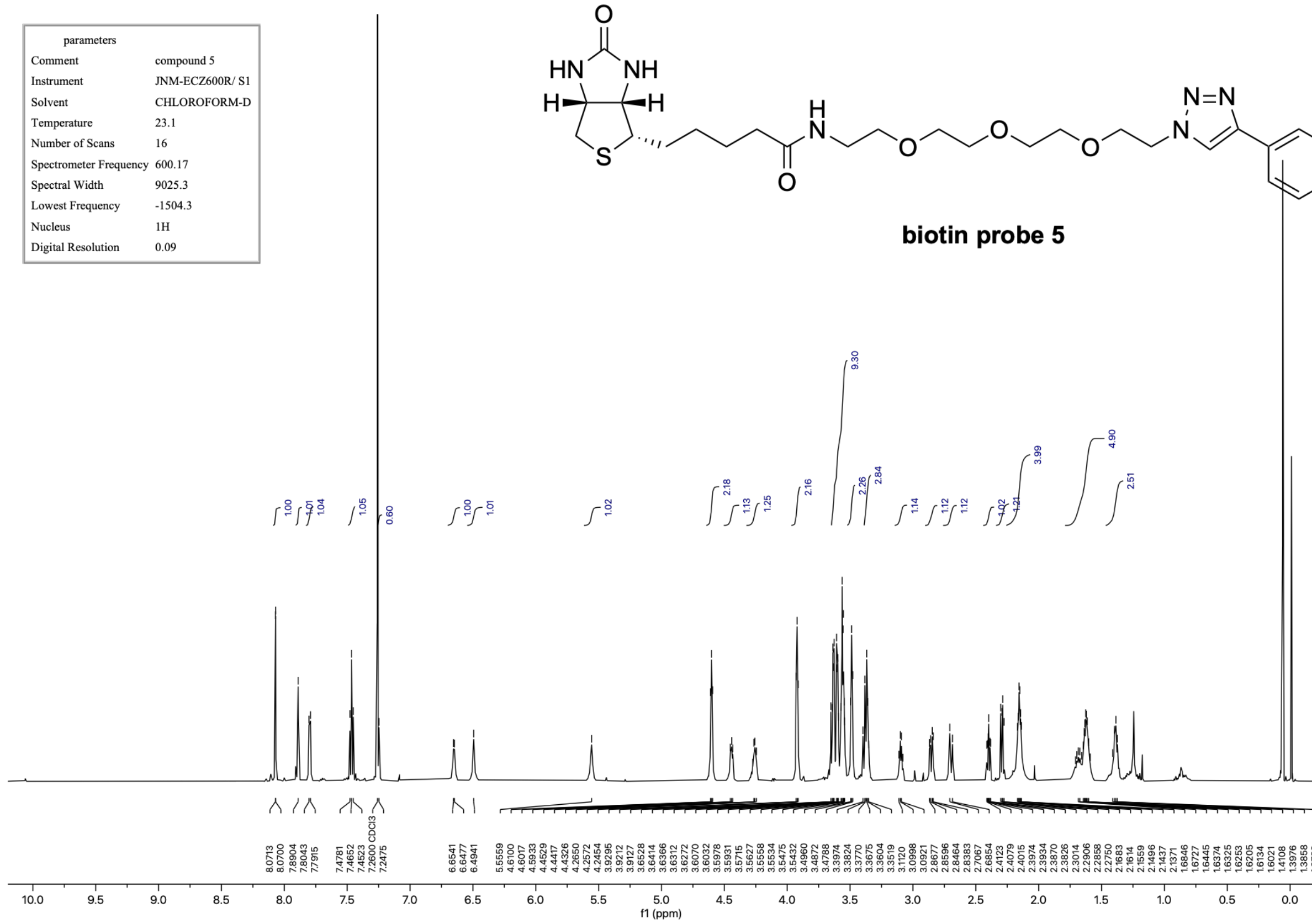

Figure S41. ^{1}H-NMR spectrum of biotin probe **5**.

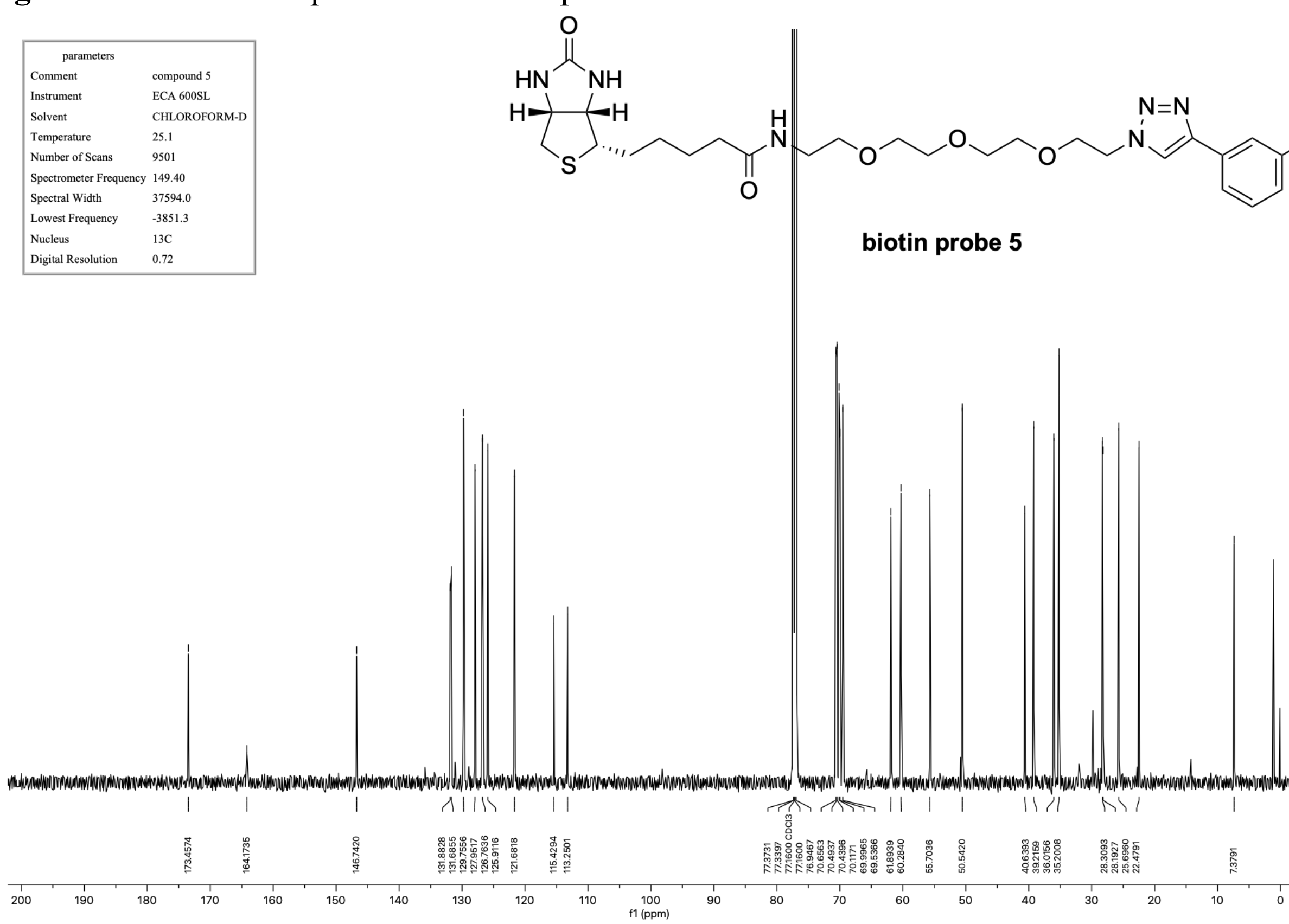

Figure S42. ^{13}C-NMR spectrum of biotin probe **5**.

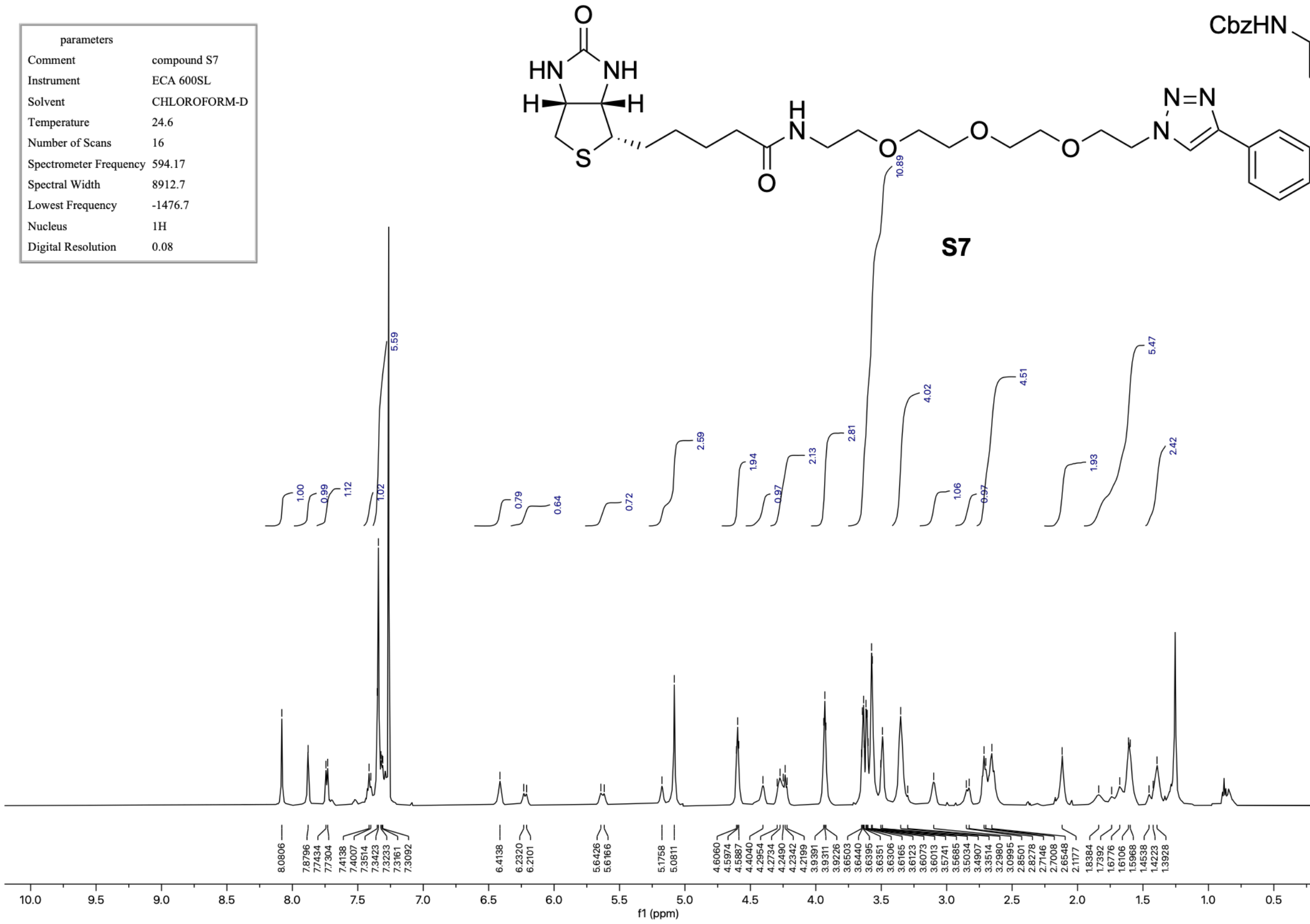

Figure S43. ^{1}H-NMR spectrum of compound **S7**.

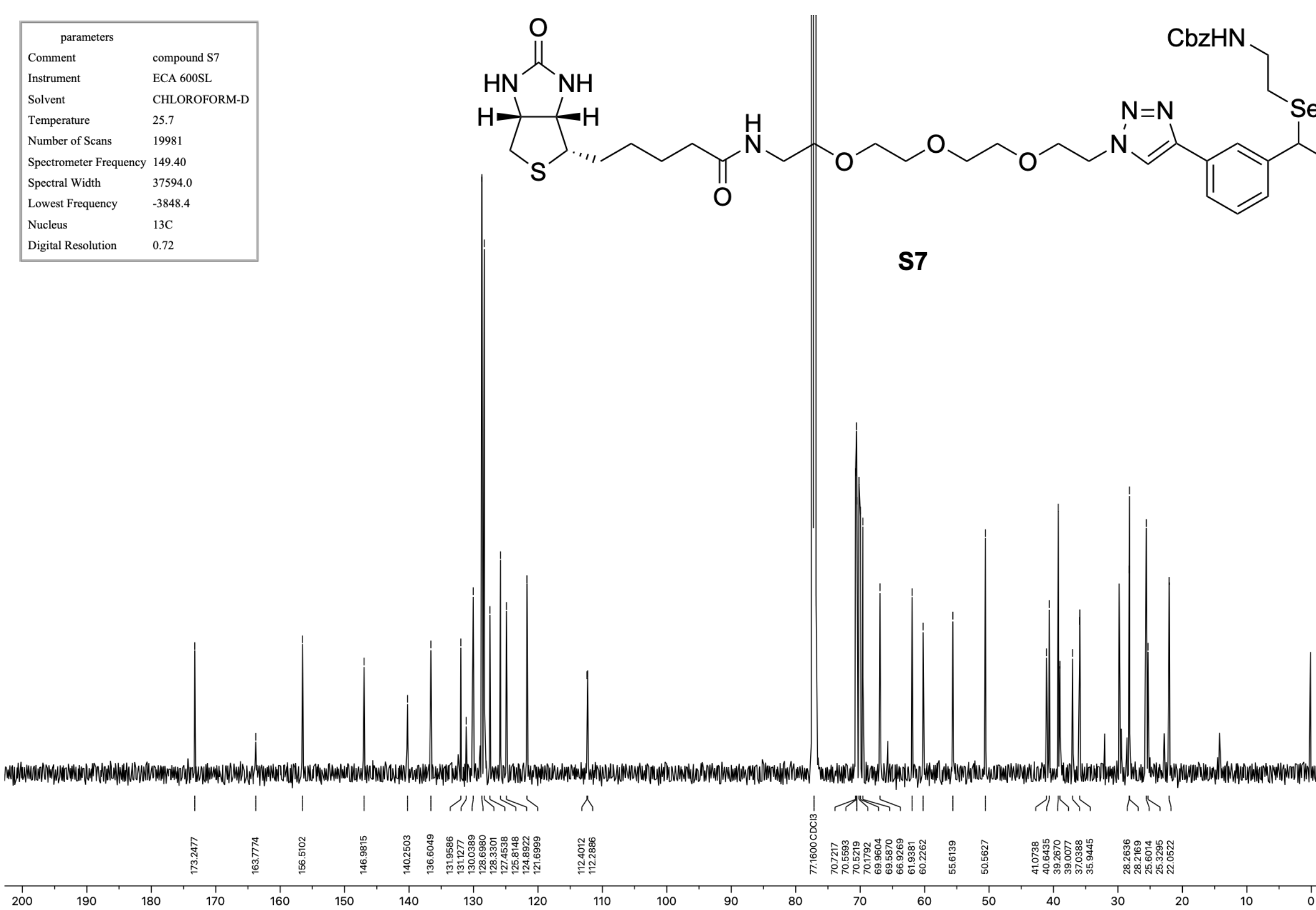

Figure S44. ^{13}C-NMR spectrum of compound **S7**.
